# Medium and long-term risks of specific cardiovascular diseases in survivors of 20 adult cancers: a population-based cohort study using multiple linked UK electronic health records databases

**DOI:** 10.1016/S0140-6736(19)31674-5

**Published:** 2019-09-21

**Authors:** Helen Strongman, Sarah Gadd, Anthony Matthews, Kathryn E Mansfield, Susannah Stanway, Alexander R Lyon, Isabel dos-Santos-Silva, Liam Smeeth, Krishnan Bhaskaran

**Affiliations:** a Department of Non-Communicable Diseases Epidemiology, London School of Hygiene & Tropical Medicine, London, UK; b School of Geography, University of Leeds, Leeds, UK; c Institute of Environmental Medicine, Karolinska Institute, Stockholm, Sweden; d Breast Unit, Royal Marsden Hospital, London, UK; e Royal Brompton Hospital and National Heart and Lung Institute, Imperial College London, London, UK; f Health Data Research UK, London, UK

## Abstract

**Background:**

The past few decades have seen substantial improvements in cancer survival, but concerns exist about long-term cardiovascular disease risk in survivors. Evidence is scarce on the risks of specific cardiovascular diseases in survivors of a wide range of cancers to inform prevention and management. In this study, we used large-scale electronic health records data from multiple linked UK databases to address these evidence gaps.

**Methods:**

For this population-based cohort study, we used linked primary care, hospital, and cancer registry data from the UK Clinical Practice Research Datalink to identify cohorts of survivors of the 20 most common cancers who were 18 years or older and alive 12 months after diagnosis and controls without history of cancer, matched for age, sex, and general practice. We compared risks for a range of cardiovascular disease outcomes using crude and adjusted Cox models. We fitted interactions to investigate effect modification, and flexible parametric survival models to estimate absolute excess risks over time.

**Findings:**

Between Jan 1, 1990, and Dec 31, 2015, 126 120 individuals with a diagnosis of a cancer of interest still being followed up at least 1 year later were identified and matched to 630 144 controls. After exclusions, 108 215 cancer survivors and 523 541 controls were included in the main analyses. Venous thromboembolism risk was elevated in survivors of 18 of 20 site-specific cancers compared with that of controls; adjusted hazard ratios (HRs) ranged from 1·72 (95% CI 1·57–1·89) in patients after prostate cancer to 9·72 (5·50–17·18) after pancreatic cancer. HRs decreased over time, but remained elevated more than 5 years after diagnosis. We observed increased risks of heart failure or cardiomyopathy in patients after ten of 20 cancers, including haematological (adjusted HR 1·94, 1·66–2·25, with non-Hodgkin lymphoma; 1·77, 1·50–2·09, with leukaemia; and 3·29, 2·59–4·18, with multiple myeloma), oesophageal (1·96, 1·46–2·64), lung (1·82, 1·52–2·17) kidney (1·73, 1·38–2·17) and ovarian (1·59, 1·19–2·12). Elevated risks of arrhythmia, pericarditis, coronary artery disease, stroke, and valvular heart disease were also observed for multiple cancers, including haematological malignancies. HRs for heart failure or cardiomyopathy and venous thromboembolism were greater in patients without previous cardiovascular disease and in younger patients. However, absolute excess risks were generally greater with increasing age. Increased risks of these outcomes seemed most pronounced in patients who had received chemotherapy.

**Interpretation:**

Survivors of most site-specific cancers had increased medium-term to long-term risk for one or more cardiovascular diseases compared with that for the general population, with substantial variations between cancer sites.

**Funding:**

Wellcome Trust and Royal Society.

## Introduction

Improvements in cancer survival in the past few decades have resulted in a large and growing population of long-term cancer survivors; about half of patients diagnosed with cancer in high-income settings are now expected to survive for 10 years or longer.^1^ However, significant concerns exist that there might be increased medium-term to long-term risks of cardiovascular diseases after cancer diagnosis, driven by cardiotoxic treatment effects, mechanisms directly related to cancer biology, and shared risk factors.^2^, ^3^, ^4^, ^5^, ^6^, ^7^, ^8^

Although randomised trials have shown short-term to medium-term adverse cardiovascular effects of some specific cancer treatments, such studies cannot quantify the broader differences in risk between cancer survivors and individuals with no history of cancer.^2^ Several observational studies^9^ comparing survivors of adolescent and young adult-onset cancer with age-matched controls with no history of cancer or controls from a general population found substantially increased risks of cardiovascular disease during the time after survival. However, results varied by age, suggesting that estimates from these studies might not generalise to adult-onset cancer survivors. Long-term follow-up after cancer is increasingly available through linked electronic health record databases. To date, most studies in adults have focused on a composite cardiovascular disease outcome, suggesting that people with a history of some, but not all, cancers are at increased risk of cardiovascular disease.^10^ However, evidence exists of heterogeneous associations between different cancers and different cardiovascular disease outcomes.^11^ Few individual studies have examined risks of multiple specific outcomes of cardiovascular disease in survivors of a wide range of site-specific cancers to show the broader patterns of risk within a single methodologically coherent framework. Additionally, few studies were able to include data on key shared risk factors for cancer and cardiovascular disease, such as smoking and body-mass index (BMI), to help delineate the drivers of cardiovascular disease risk in cancer survivors.

**Research in context**

**Evidence before this study**

We searched PubMed and OVID MEDLINE for epidemiological studies, reviews, and guidelines published in English from Jan 1, 2008, to Dec 31, 2018, using search terms for cardiovascular outcomes and cancer (details of search terms are in the appendix [pp 45–46]). We identified articles that provided estimates comparing risks of any of the specific cardiovascular disease outcomes included in our study between adult survivors of one or more site-specific cancers and controls without a history of cancer. 21 studies were included; eight focused on cardiovascular risks in patients after breast cancer and seven included multiple cancer sites, but six of these were restricted to teenage-onset and young adult-onset cancers (appendix pp 47–51). Relative risk estimates from previous studies were extracted and are displayed alongside estimates from our study in the appendix (pp 52–59). The most commonly studied outcomes were coronary artery disease and stroke: 95% CIs for most estimates crossed the null but were too wide to exclude modest associations. Some evidence was found of increased risks of both outcomes in patients after non-Hodgkin lymphoma, whereas stroke risk was markedly elevated for survivors of CNS cancer in all three relevant studies. Increased risks of heart failure or cardiomyopathy were observed in patients after several types of cancers, notably haematological malignancies. Elevated risks of venous thromboembolism were seen in most studies of this outcome, though few estimates were available per cancer site. Little previous evidence was available for other outcomes.

**Added value of this study**

Survivors of most site-specific cancers had increased risk of one or more cardiovascular disease outcomes, but patterns of risk varied by cancer site and by outcome. We found a substantially increased risk of venous thromboembolism for most cancers and increased risks of heart failure or cardiomyopathy in survivors of half of cancers investigated. Risks of other cardiovascular disease outcomes, including coronary artery disease and stroke, were elevated in survivors of several specific cancers, including haematological malignancies. Associations between cancer and cardiovascular risk did not appear to be explained by shared risk factors, but exploratory analyses highlighted chemotherapy as an important driver of risk.

**Implications of all the available evidence**

Our study is one of the largest to date to compare risks for a wide range of cardiovascular disease outcomes between adult survivors of multiple site-specific cancers and controls with no history of cancer, with a consistent methodological approach that allowed us to reveal detailed patterns of risk. Our findings show that more tailored strategies to minimise and manage cardiovascular risk are needed for people who survive cancer.

To address these evidence gaps, we aimed to use large-scale electronic health records data from multiple linked UK databases to quantify the absolute and relative risks of a comprehensive range of cardiovascular diseases in survivors of the 20 most common site-specific adult cancers, covering more than 90% of all cancer diagnoses,^1^ compared with cancer-free controls from the general population. We also investigated the extent to which relative risk differences are driven by shared risk factors, demographic characteristics, and use of chemotherapy and radiotherapy.

## Methods

### Study design and participants

In this population-based cohort study, we identified 20 matched cohorts to compare risks of cardiovascular disease outcomes in survivors of the 20 most common site-specific cancers^1^ with those of cancer-free controls, using Clinical Practice Research Datalink primary care data (CPRD GOLD) linked to national data on hospital admissions from the Hospital Episode Statistics Admitted Patient Care (HES APC) database, cancer registrations from the National Cancer Registration and Analysis Service (NCRAS), death registrations—including cause of death information—from the Office of National Statistics mortality database, and postcode-based index of Multiple Deprivation data.^12^, ^13^ CPRD GOLD comprises prospectively collected primary care records from general practices in the UK that use Vision software and have chosen to participate; this database includes approximately 7% of the UK population and captures coded diagnoses and care events (using Read coding),^14^ prescriptions issued in primary care, and numerical test results (eg, blood pressure readings). Secondary care diagnoses are typically reported back to the general practitioner (GP) and recorded in the primary care record if they are considered to affect the ongoing care of the patient. Linked HES APC and NCRAS data added information coded according to the International Classification of Diseases, version 10 (ICD-10), on hospital diagnoses and cancer registrations, improving capture of clinical events. Linked data sources covered England only between Jan 1, 1990, and Dec 31, 2015; therefore our primary analyses were restricted to this setting and time period.

**Figure 1:**
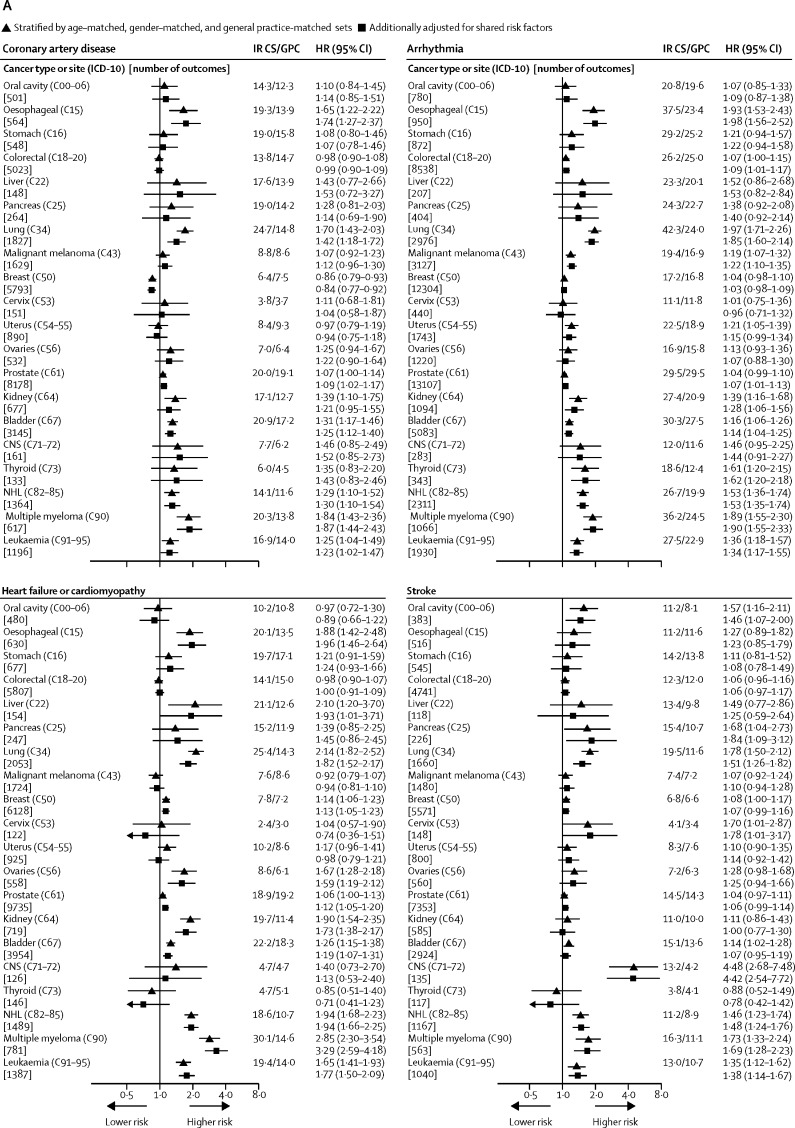

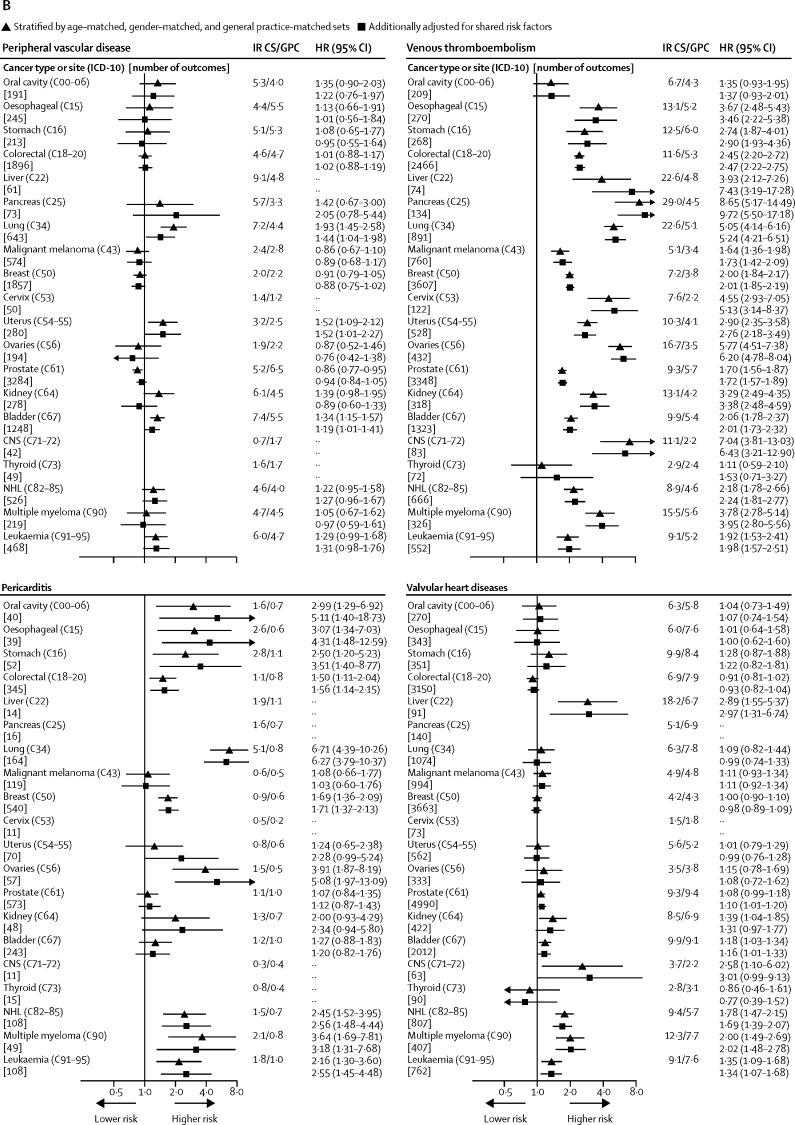
Absolute and relative risks of cardiovascular disease outcomes in cancer survivors compared with those of general population controls Cancer types or sites are ordered according to corresponding codes from the International Classification of Diseases, version 10 (ICD-10). Cancer sites without data had too few events for estimation. Arrowheads represent a CI limit lower than 0·5 or higher than 12. HR=hazard ratio. IR=incidence per 1000 patient-years. CS=cancer survivors. GPC=general population controls. NHL=Non-Hodgkin lymphoma.

We used CPRD GOLD, HES APC, and NCRAS to identify patients aged 18 years or older with an incident primary site-specific cancer of interest (list of cancers is presented in figure 1). Incident cancer was defined as the first code for cancer at the site of interest in any of the linked databases, at least 1 year after the start of research-quality follow-up (research-quality follow-up is determined by CPRD on the basis of a set of basic practice and patient-level data quality checks).^12^ Patients with no information on smoking, BMI, or index of Multiple Deprivation (an area-based proxy for socioeconomic status) were excluded from the analysis. Patients were also excluded if they had less than 1 year of follow-up after their incident cancer diagnosis.

This study was approved by the London School of Hygiene & Tropical Medicine Ethics Committee (12042) and the Independent Scientific Advisory Committee for the Medicines and Healthcare products Regulatory Agency database research (16_274). The study protocol is available in the appendix (pp 63–76). CPRD supplies anonymised data for public health research; therefore individual patient consent was not required for this study.^13^

### Procedures

Cancer survivors entered the study 1 year after diagnosis (index date) and were matched on age (±3 years), sex, and general practice with up to five controls with no history of cancer and at least 24 months of continuous preceding follow-up on the index date of the matched cancer survivor (mirroring the requirement on cancer survivors to have 1 year of follow-up before and after cancer diagnosis to enter the study on the index date). If a control went on to receive an incident cancer diagnosis during follow-up, they were no longer available as a control at that time but could then contribute as a site-specific cancer survivor (if the cancer was at one of the 20 sites of interest) with their own set of matched controls.

The outcomes of the study were fatal or non-fatal coronary artery disease (angina, myocardial infarction, revascularisation procedures, and sudden cardiac arrest), stroke (haemorrhagic and ischaemic stroke), arrhythmia, venous thromboembolism (deep vein thrombosis and pulmonary embolism), heart failure and cardiomyopathy combined, pericarditis, valvular heart disease, and peripheral vascular disease. Component outcomes within coronary artery disease, venous thromboembolism, and heart failure or cardiomyopathy were also analysed separately. We identified outcomes using Read codes within CPRD GOLD, ICD-10 codes within HES APC, and ICD-9–10 codes within the ONS mortality database.

Demographics and shared risk factors, including smoking, BMI, and comorbidities, were identified at the cancer diagnosis date for the cancer survivor group and at 1 year before the index date for the matched cohort. Full covariate definitions are provided in the appendix (pp 4–6). Code lists for all study variables are available online.

### Statistical analysis

For all analyses of each outcome, patients were followed up from the index date (ie, the first anniversary of diagnosis for the cancer survivor in each matched set) until the earliest occurrence of: death, end of research-quality follow-up, end of study period, or first occurrence of an outcome. Patients with a record of an outcome of interest before the index date were excluded from the analysis of that outcome. We calculated crude incidences for each cardiovascular disease outcome in cancer survivors and matched controls, with separate models for each cancer site. To relate site-specific cancer history to risk of each cardiovascular disease outcome, we then fitted Cox proportional hazards models with time since index date as the timescale, initially accounting only for matching factors (age, sex, and general practice) through stratification by matched set and then additionally adjusting for shared risk factors and demographics listed in table 1. Severe mental illness, rheumatoid arthritis, and systemic lupus erythematosus were considered but were not included in the final models because they were rare within the study population. We made additional adjustments in analyses of specific cancers, namely for hysterectomy and previous hormone replacement therapy (in analyses of all female-specific cancers), chronic liver disease (liver cancer analysis), immunosuppression (non-Hodgkin lymphoma analysis), and sclerosis and chronic obstructive pulmonary disease (lung cancer analysis). A directed acyclic graph in the appendix (p 7) sets out the postulated causal relationships between covariates and thus, the causal framework assumed by our models.^15^

**Table 1:** Characteristics of cancer survivors and matched general population controls (all cancer sites)

		**Cancer survivors**	**Controls**
Total number of patients		108 215	523 541
Time from cancer diagnosis or baseline to end of follow-up			
	Mean (SD)	5·7 (4·1)	6·4 (4·2)
	Median (IQR)	4·5 (2·5–7·9)	5·4 (3·1–8·8)
	Range	1·0–26·5	1·0–26·6
Total person-years included* (million years)		0·507	2·839
Age (years)†			
	Mean (SD)	66·1 (13·3)	66·0 (13·2)
	Median (IQR)	67·0 (58·0–76·0)	67·0 (58·0–76·0)
Age (years)‡			
	18–59	31 057 (28·7%)	150 257 (28·7%)
	60–79	60 347 (55·8%)	293 080 (56·0%)
	≥80	16 811 (15·5%)	80 204 (15·3%)
Sex†‡			
	Men	51 541 (47·6%)	245 760 (46·9%)
	Women	56 674 (52·4%)	277 781 (53·1%)
Index of Multiple Deprivation quintile (patient area)‡§¶			
	1 (least deprived)	27 755 (25·6%)	133 023 (25·4%)
	2	25 607 (23·7%)	123 322 (23·6%)
	3	22 926 (21·2%)	111 594 (21·3%)
	4	17 902 (16·5%)	87 408 (16·7%)
	5 (most deprived)	14 025 (13·0%)	68 194 (13·0%)
Year of cancer diagnosis†‡			
	1989–98	11 277 (10·4%)	53 298 (10·2%)
	1999–2003	24 487 (22·6%)	117 541 (22·5%)
	2004–08	37 369 (34·5%)	181 127 (34·6%)
	2009–14	35 082 (32·4%)	171 575 (32·8%)
Smoking status‡¶			
	Non-smoker	47 682 (44·1%)	245 275 (46·8%)
	Current smoker	19 433 (18·0%)	89 509 (17·1%)
	Ex-smoker	41 100 (38·0%)	188 757 (36·1%)
Heavy drinker¶		3806 (3·5%)	22 457 (4·3%)
Body-mass index‡¶			
	Underweight	1850 (1·7%)	8491 (1·6%)
	Healthy weight	40 393 (37·3%)	196 789 (37·6%)
	Overweight	42 060 (38·9%)	203 457 (38·9%)
	Obese	23 912 (22·1%)	114 804 (21·9%)
Diabetes¶		11 862 (11·0%)	55 091 (10·5%)
Hypertension‡¶		26 246 (24·3%)	116 313 (22·2%)
Previous cardiovascular disease‡¶‖		32 826 (30·3%)	151 436 (28·9%)
Cardiovascular treatments¶			
	Statins	25 947 (24·0%)	123 947 (23·7%)
	β blockers	17 316 (16·0%)	83 748 (16·0%)
	Angiotensin converting enzyme inhibitors	19 782 (18·3%)	93 542 (17·9%)
	Angiotensin II receptor blockers	7618 (7·0%)	34 425 (6·6%)
	Non-steroidal anti-inflammatory drugs	11 940 (11·0%)	52 875 (10·1%)
Previous migraine¶		7125 (6·6%)	34 313 (6·6%)
Chronic kidney disease¶		16 977 (15·7%)	71 655 (13·7%)

Data are n (%) and represent status at cancer diagnosis date for survivors, with the same date used for matched controls (baseline), unless specified otherwise. Number of patients by cancer type or site (with International Classification of Diseases codes, version 10): 1584 oral cavity (C00–06), 1794 oesophageal (C15), 1507 stomach (C16), 14 216 colorectal (C18–20), 554 liver (C22), 864 pancreas (C25), 5369 lung (C34), 7098 malignant melanoma (C43), 25 633 breast (C50), 1209 cervix (C53), 3440 uterus (C54–55), 2710 ovaries (C56), 20 709 prostate (C61), 2197 kidney (C64), 7712 bladder (C67), 906 CNS (>C71–72), 1028 thyroid (C73), 4423 non-Hodgkin lymphoma (C82–85), 1843 multiple myeloma (C90), 3419 leukaemia (C91–95).

* Index date to end of follow-up.

† Matching variable.

‡ Interactions investigated.

§ Index of multiple deprivation is an area-based proxy for socioeconomic status.

¶ Shared risk factor; additional variables for specific cancers include hysterectomy and hormone replacement therapy (female-specific cancers), chronic liver disease (liver cancer), immunosuppression (non-Hodgkin lymphoma), and systemic sclerosis and chronic obstructive pulmonary disease (lung cancer).

‖ 34% of cancer survivors and 31% of controls had a history of previous cardiovascular disease at the index date (1 year after diagnosis or baseline).

We investigated the modification of the effect of cancer survival on cardiovascular disease risk by the following variables by fitting interaction terms and doing likelihood ratio tests for interaction: age group, sex, smoking status, BMI, other pre-existing cardiovascular disease at index date, hypertension at index, Index of Multiple Deprivation, and calendar period. Proportional hazards were checked by fitting interactions with time since index date. Definitions of interaction terms are provided in the appendix (pp 4–6). These analyses of effect modification were restricted to the nine most common cancers (colorectal, lung, malignant melanoma, breast, uterus, prostate, bladder, non-Hodgkin lymphoma, and leukaemia) and the outcomes of coronary artery disease, stroke, arrhythmia, heart failure or cardiomyopathy, and venous thromboembolism, because power was too low for other combinations. For the six of nine cancers for which at least 75% of tumours were recorded in NCRAS, we also explored the role of cancer treatment by subdividing cancer survivors according to recorded receipt of chemotherapy and radiotherapy in the period between diagnosis and index date (as recorded by cancer registry, which aims to record the first 6 months of treatments) and estimating separate hazard ratios (HRs) for each group. Absolute cardiovascular disease incidences were summarised and compared with expected incidences by first fitting flexible parametric survival models for each cancer–outcome combination, with exposure (cancer survivor *vs* control) included as a time-dependent effect and adjustment for all covariates included in our Cox models, and then predicting incidence over time at both levels of exposure, with all other covariates fixed at the mean values in the cancer survivor group.^16^ Finally, we calculated cumulative incidence of cardiovascular disease at 5 years from diagnosis in cancer survivor and control groups, allowing for the presence of competing risks of non-cardiovascular disease death.^17^

We did sensitivity analyses based on our primary Cox models. First, we repeated the analysis using only unlinked data from CPRD GOLD, which allowed a larger UK-wide population to be drawn (because only a proportion of CPRD has linkage available). This extended the analysis to the whole of the UK and to the period January, 1987, to June, 2017, but at the expense of a probably higher misclassification of cancer diagnoses and cardiovascular disease outcomes. Second, we repeated the analysis excluding people without at least two blood pressure measures before the index date, rather than classifying these individuals as normotensive (appendix pp 4–6). Lastly, we repeated the analysis using a more granular breakdown when adjusting for alcohol, in case of residual confounding in our initial classification (appendix pp 4–6). Statistical analyses were done in Stata MP, version 15.

### Role of the funding source

The funder of the study had no role in study design, data collection, data analysis, data interpretation, or writing of the report. The corresponding author had full access to all the data in the study and had final responsibility for the decision to submit for publication.

## Results

**Figure 2:**
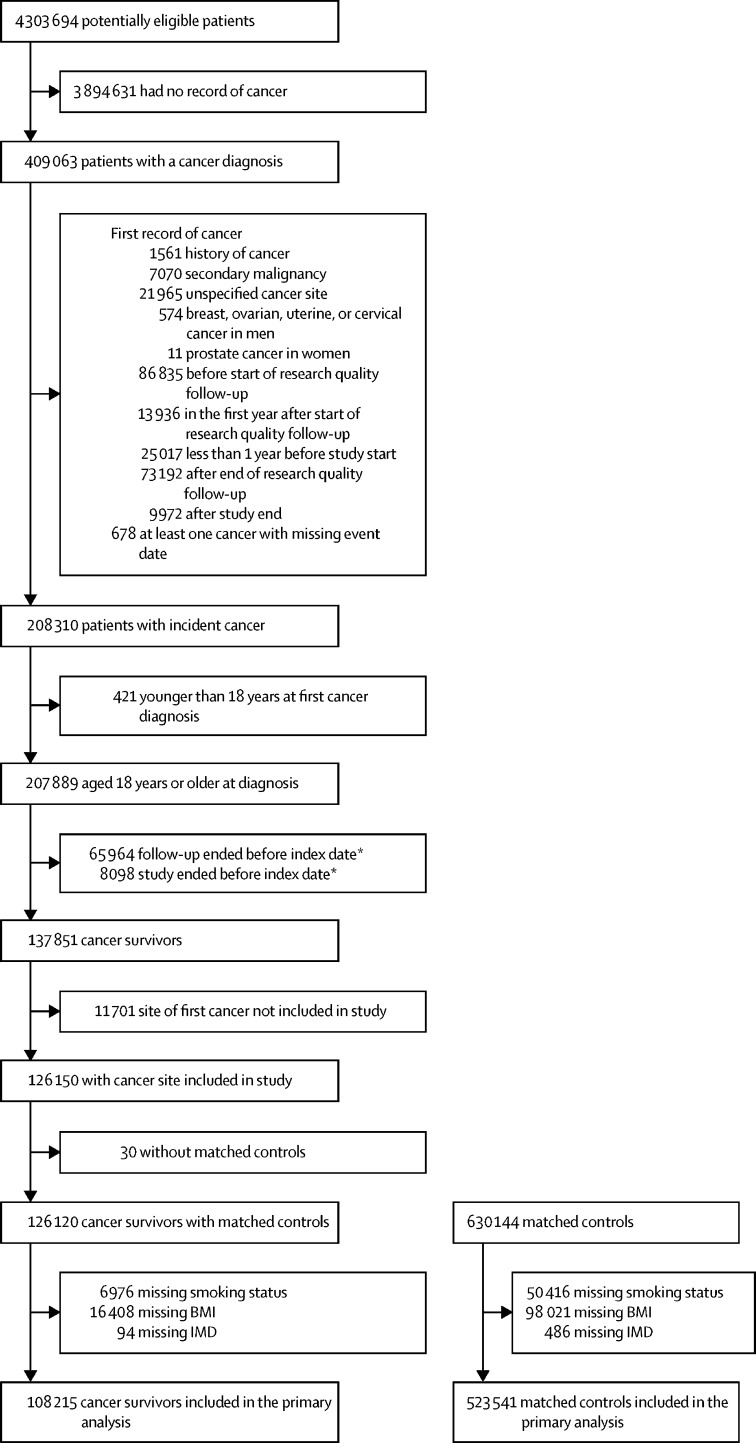
Study cohort profile for primary analysis Where multiple exclusion reasons are listed, some patients met more than one listed criteria. BMI=body-mass index. IMD=Index of multiple deprivation. *Index date was 1 year after date of diagnosis.

Between Jan 1, 1990, and Dec 31, 2015, 126 120 individuals with an incident diagnosis of a site-specific cancer of interest, still being followed up at least 1 year later were identified and matched to 630 144 controls with no history of cancer. 17 905 (14·2%) cancer survivors and 106 603 (16·9%) controls were then excluded because of missing smoking or BMI data, leaving 631 756 individuals in the final analysis cohort (figure 2, table 1, appendix p 8). 115 886 (18·3%) individuals were still under follow-up at least 10 years after diagnosis (16 428 [15·2%] of 108 215 cancer survivors and 99 458 [19·0%] of 523 541 controls). Baseline smoking, hypertension, previous history of cardiovascular disease, and chronic kidney disease were marginally more prevalent in cancer survivors than in matched controls, whereas prevalence of overweight and obesity was similar between groups and prevalence of heavy drinking was lower in cancer survivors than in controls. Baseline characteristics of the matched cohorts specific for individual cancer sites are presented in the appendix (pp 9–28).

We calculated incidences and cause-specific HRs adjusted for age and sex alone and then additionally for shared risk factors (figure 1). Adjustment for shared risk factors had little effect on HRs. In adjusted models, we observed strong evidence (p<0·01) of increased risk of incident venous thromboembolism in survivors of 18 of 20 cancers compared with that of general population controls, as well as increased risks of heart failure or cardiomyopathy (in ten of 20 cancers), arrhythmia (eight of 20), pericarditis (eight of 15 cancers with enough data for estimation), coronary artery disease (five of 20 cancers), stroke (five of 20), and valvular heart disease (three of 18 cancers with enough data; figure 1). Survivors of haematological malignancies had increased risks across all of these outcomes, whereas the pattern of risks was more varied for survivors of other types of cancer. For peripheral vascular disease, we found no strong evidence of association and the observed HRs were in both directions. Results for component outcomes of venous thromboembolism, heart failure or cardiomyopathy, and coronary artery disease were broadly similar to those of the grouped outcomes, but with lower statistical power (appendix pp 29–30).

**Figure 3:**
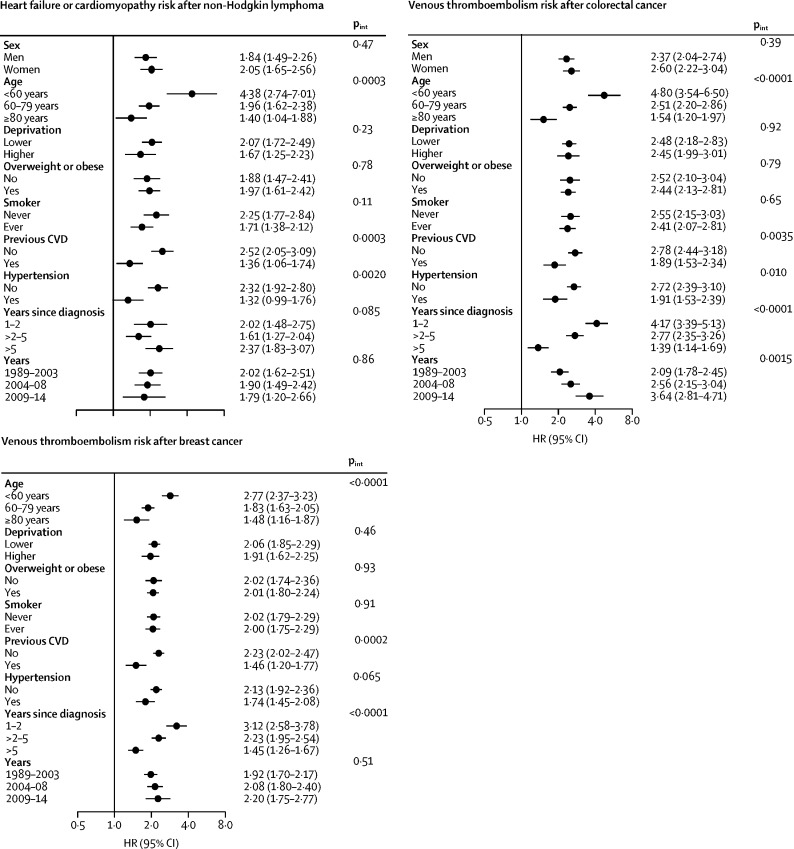
Relative risk of cardiovascular disease outcomes in cancer survivors compared with that of the general population, with effect modification (selected models) p_int_=p for interaction (likelihood ratio test). CVD=cardiovascular disease. HR=hazard ratio.

For the coronary artery disease and stroke outcomes, we found little statistical evidence of effect modification by any of the factors assessed (appendix pp 31–32). We found strong evidence that the increased risk of heart failure after non-Hodgkin lymphoma was more pronounced at younger ages and in individuals with no previous cardiovascular disease or hypertension than in older people or in individuals with a history of those conditions (figure 3); similar patterns by age were seen for breast and lung cancer (appendix p 33). We found evidence of a more pronounced increased risk of arrhythmia after colorectal cancer and non-Hodgkin lymphoma in overweight or obese individuals compared with that of patients with lower weight (appendix p 34). Lastly, we found strong evidence that younger people and those with no history of cardiovascular disease had the greatest increased risk of venous thromboembolism after breast and colorectal cancer. These risks attenuated with time, but remained significantly elevated more than 5 years after diagnosis (figure 3); similar patterns were seen for several other cancer sites, although with more statistical uncertainty (appendix p 35). In a post-hoc analysis of cancers associated with increased risk of venous thromboembolism more than 5 years after diagnosis, we introduced another time category split at 10 years: risk of venous thromboembolism remained elevated 10 years after diagnosis for colorectal cancer (HR 1·61, 95% CI 1·10–2·35; p=0·014), non-Hodgkin lymphoma (3·94, 1·65–9·43; p=0·0020), and malignant melanoma (2·00, 1·14–3·49; p=0·015), but not for breast, bladder, or prostate cancers; there were too few events 10 years after diagnosis of lung cancer and leukaemia for estimation.

**Figure 4:**
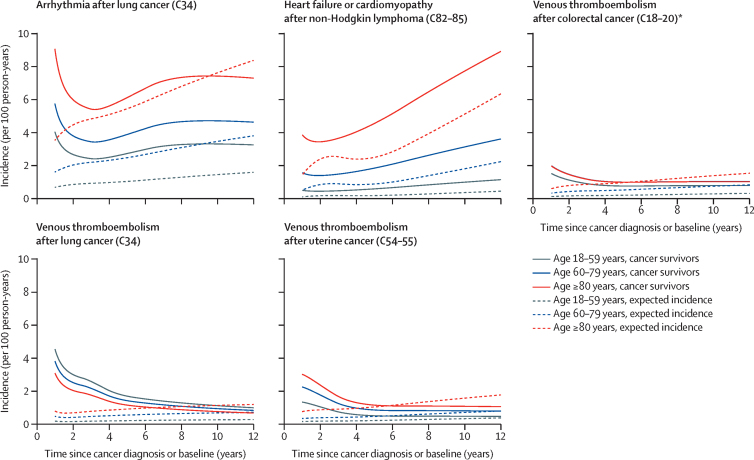
Absolute incidence of cardiovascular disease outcomes compared with expected incidence by age group and time since diagnosis Incidence in cancer survivors and expected incidence calculated by fitting flexible parametric survival models with exposure (cancer survivor *vs* control), covariates, and interaction between exposure and time since diagnosis and by predicting incidence for exposed or unexposed at the mean value of covariates in cancer survivors. Cancer sites defined by corresponding codes from the International Classification of Diseases, version 10. *The group of cancer survivors aged 60–79 years had incidence similar to that of the group aged ≥80 years, resulting in a seemingly absent blue line in the graph.

Absolute venous thromboembolism incidence was highest closer to the time of cancer diagnosis and reduced over time, as did incidence of arrhythmia after lung cancer, whereas heart failure or cardiomyopathy incidence increased with time after diagnosis (figure 4, appendix pp 36–38). Absolute excess risks were generally higher in the oldest age group than in younger groups: for example, in survivors of non-Hodgkin lymphoma 5 years after diagnosis, patients younger than 60 years had absolute excess risk of heart failure or cardiomyopathy of 0·4% per year (95% CI 0·2–0·6), whereas patients aged 80 years or older had excess risk of 2·1% per year (0·8–3·3); however, the opposite pattern was seen in survivors of lung cancer (table 2). 5-year cumulative incidences in the presence of the competing risk of non-cardiovascular disease death, stratified by age, are presented in the appendix (p 39).

**Table 2:** Absolute incidence of cardiovascular disease outcomes at specific timepoints after cancer diagnosis compared with expected incidence, by age group

	**18–59 years**			**60–79 years**			**≥80 years**		
	Observed incidence	Expected incidence	Excess (95% CI)	Observed incidence	Expected incidence	Excess (95% CI)	Observed incidence	Expected incidence	Excess (95% CI)
**Arrhythmia incidence at 5 years**									
Colorectal (C18–20)	1·2	1·0	0·2 (−0·0 to 0·5)	2·5	2·3	0·2 (−0·0 to 0·5)	4·2	4·6	−0·4 (−1·1 to 0·3)
Lung (C34)	2·7	1·0	1·7 (0·8 to 2·6)	3·9	2·5	1·4 (0·6 to 2·2)	6·1	5·4	0·7 (−1·4 to 2·7)
Melanoma (C43)	1·0	0·8	0·1 (−0·1 to 0·3)	2·1	2·0	0·1 (−0·3 to 0·4)	3·9	4·0	−0·2 (−1·2 to 0·8)
Breast (C50)	1·1	1·0	0·1 (−0·0 to 0·2)	1·9	1·8	0·1 (−0·1 to 0·2)	3·8	3·7	0·1 (−0·4 to 0·6)
Uterus (C54–55)	1·4	1·2	0·2 (−0·2 to 0·6)	2·0	2·1	−0·0 (−0·5 to 0·4)	5·1	4·1	1·0 (−0·9 to 2·9)
Prostate (C61)	1·1	1·1	−0·0 (−0·3 to 0·2)	2·6	2·5	0·1 (−0·1 to 0·3)	5·5	5·0	0·5 (−0·2 to 1·1)
Bladder (C67)	1·1	1·1	0·0 (−0·3 to 0·4)	2·8	2·5	0·3 (−0·1 to 0·7)	5·1	4·9	0·3 (−0·7 to 1·2)
NHL (C82–85)	1·3	0·8	0·5 (0·1 to 0·9)	2·9	2·1	0·8 (0·2 to 1·4)	5·7	4·4	1·4 (−0·3 to 3·0)
Leukaemia (C91–95)	1·1	1·0	0·2 (−0·2 to 0·6)	2·6	2·4	0·2 (−0·4 to 0·8)	3·9	5·0	−1·1 (−2·4 to 0·2)
**Coronary artery disease incidence at 5 years**									
Colorectal (C18–20)	0·7	0·6	0·1 (−0·1 to 0·2)	1·3	1·4	−0·2 (−0·4 to 0·0)	2·2	2·3	−0·1 (−0·5 to 0·4)
Lung (C34)	1·3	0·7	0·6 (0·0 to 1·2)	2·0	1·7	0·3 (−0·3 to 0·9)	2·9	2·7	0·2 (−1·2 to 1·5)
Melanoma (C43)	0·3	0·3	0·0 (−0·1 to 0·1)	1·2	0·9	0·3 (0·0 to 0·6)	2·1	1·9	0·2 (−0·6 to 1·0)
Breast (C50)	0·3	0·3	−0·0 (−0·1 to 0·0)	0·8	0·9	−0·1 (−0·2 to 0·0)	1·3	1·7	−0·4 (−0·7 to −0·1)
Uterus (C54–55)	0·4	0·4	−0·0 (−0·3 to 0·2)	0·7	1·0	−0·4 (−0·6 to −0·1)	2·3	1·9	0·4 (−0·8 to 1·6)
Prostate (C61)	0·9	1·0	−0·1 (−0·4 to 0·1)	1·8	1·8	0·1 (−0·1 to 0·2)	2·6	2·7	−0·1 (−0·5 to 0·3)
Bladder (C67)	1·1	0·7	0·4 (0·1 to 0·7)	2·0	1·7	0·4 (0·0 to 0·7)	2·7	2·6	0·2 (−0·5 to 0·8)
NHL (C82–85)	0·8	0·4	0·3 (0·1 to 0·6)	1·8	1·3	0·5 (0·1 to 1·0)	2·2	2·0	0·2 (−0·7 to 1·2)
Leukaemia (C91–95)	0·9	0·6	0·3 (−0·1 to 0·7)	1·8	1·5	0·4 (−0·2 to 0·9)	3·1	2·0	1·1 (−0·1 to 2·2)
**Heart failure or cardiomyopathy incidence at 5 years**									
Colorectal (C18–20)	0·3	0·3	0·0 (−0·1 to 0·1)	1·0	1·1	−0·0 (−0·2 to 0·1)	2·6	3·0	−0·4 (−0·9 to 0·1)
Lung (C34)	1·1	0·3	0·8 (0·3 to 1·3)	2·2	1·3	0·8 (0·3 to 1·4)	4·1	4·1	0·1 (−1·5 to 1·6)
Melanoma (C43)	0·1	0·1	−0·0 (−0·1 to 0·0)	0·6	0·7	−0·1 (−0·3 to 0·1)	2·3	2·5	−0·2 (−0·9 to 0·5)
Breast (C50)	0·2	0·1	0·1 (0·1 to 0·2)	0·7	0·7	0·1 (−0·0 to 0·2)	2·1	2·3	−0·1 (−0·5 to 0·2)
Uterus (C54–55)	0·2	0·2	−0·0 (−0·1 to 0·1)	0·7	0·8	−0·1 (−0·3 to 0·2)	3·3	2·7	0·6 (−0·7 to 1·9)
Prostate (C61)	0·4	0·4	−0·1 (−0·2 to 0·1)	1·4	1·4	0·0 (−0·1 to 0·2)	4·0	3·6	0·3 (−0·2 to 0·8)
Bladder (C67)	0·4	0·3	0·1 (−0·1 to 0·3)	1·7	1·5	0·3 (0·0 to 0·5)	4·2	3·9	0·3 (−0·5 to 1·1)
NHL (C82–85)	0·6	0·2	0·4 (0·2 to 0·6)	1·8	0·9	1·0 (0·6 to 1·3)	4·6	2·5	2·1 (0·8 to 3·3)
Leukaemia (C91–95)	0·5	0·2	0·3 (0·0 to 0·5)	1·6	1·1	0·5 (0·1 to 0·9)	3·8	3·5	0·4 (−0·8 to 1·5)
**Stroke incidence at 5 years**									
Colorectal (C18–20)	0·4	0·3	0·1 (−0·0 to 0·2)	1·1	1·1	−0·0 (−0·2 to 0·1)	2·1	2·5	−0·4 (−0·8 to 0·0)
Lung (C34)	1·1	0·3	0·8 (0·4 to 1·3)	1·6	1·3	0·4 (−0·0 to 0·8)	2·6	2·6	0·0 (−1·0 to 1·1)
Melanoma (C43)	0·3	0·2	0·1 (−0·0 to 0·2)	0·8	0·8	−0·0 (−0·2 to 0·2)	1·9	2·1	−0·2 (−0·9 to 0·5)
Breast (C50)	0·2	0·2	0·0 (−0·0 to 0·1)	0·8	0·7	0·0 (−0·1 to 0·1)	2·0	2·1	−0·1 (−0·5 to 0·2)
Uterus (C54–55)	0·2	0·2	−0·0 (−0·2 to 0·1)	0·7	0·8	−0·0 (−0·3 to 0·2)	1·8	1·7	0·1 (−0·8 to 1·0)
Prostate (C61)	0·6	0·5	0·1 (−0·0 to 0·3)	1·3	1·2	0·0 (−0·1 to 0·2)	2·6	2·5	0·1 (−0·3 to 0·5)
Bladder (C67)	0·4	0·3	0·1 (−0·1 to 0·3)	1·4	1·3	0·1 (−0·2 to 0·3)	3·0	2·8	0·2 (−0·5 to 0·8)
NHL (C82–85)	0·4	0·2	0·2 (0·0 to 0·4)	1·2	1·0	0·2 (−0·1 to 0·6)	2·9	2·4	0·5 (−0·5 to 1·6)
Leukaemia (C91–95)	0·6	0·3	0·3 (0·1 to 0·6)	1·2	0·9	0·3 (−0·1 to 0·6)	2·0	2·3	−0·3 (−1·1 to 0·5)
**Venous thromboembolism incidence at 1 year**									
Colorectal (C18–20)	1·5	0·1	1·4 (0·9 to 1·8)	2·0	0·3	1·7 (1·2 to 2·1)	2·0	0·6	1·4 (0·8 to 1·9)
Lung (C34)	4·5	0·2	4·3 (2·9 to 5·8)	3·8	0·5	3·3 (2·4 to 4·2)	3·1	0·8	2·3 (0·9 to 3·7)
Melanoma (C43)	0·3	0·1	0·3 (0·1 to 0·4)	0·8	0·2	0·6 (0·2 to 1·0)	1·0	0·4	0·5 (−0·1 to 1·2)
Breast (C50)	0·7	0·1	0·6 (0·5 to 0·8)	1·1	0·3	0·9 (0·7 to 1·1)	1·7	0·5	1·2 (0·7 to 1·6)
Uterus (C54–55)	1·3	0·2	1·2 (0·4 to 2·0)	2·3	0·3	1·9 (0·8 to 3·1)	3·0	0·8	2·3 (0·3 to 4·2)
Prostate (C61)	0·4	0·2	0·2 (0·1 to 0·4)	0·9	0·3	0·5 (0·3 to 0·8)	1·3	0·5	0·8 (0·3 to 1·2)
Bladder (C67)	1·2	0·1	1·1 (0·5 to 1·7)	1·9	0·3	1·6 (0·9 to 2·4)	1·7	0·5	1·2 (0·5 to 1·9)
NHL (C82–85)	0·7	0·1	0·6 (0·0 to 1·2)	1·4	0·3	1·1 (0·0 to 2·1)	1·7	0·7	1·0 (−0·4 to 2·4)
Leukaemia (C91–95)	0·4	0·1	0·3 (−0·0 to 0·6)	0·8	0·4	0·4 (−0·2 to 0·9)	0·8	0·7	0·2 (−0·6 to 0·9)
**Venous thromboembolism incidence at 5 years**									
Colorectal (C18–20)	0·8	0·2	0·6 (0·4 to 0·7)	1·0	0·5	0·5 (0·3 to 0·6)	1·0	1·0	0·0 (−0·2 to 0·3)
Lung (C34)	1·7	0·2	1·5 (0·8 to 2·2)	1·4	0·6	0·9 (0·4 to 1·4)	1·2	0·9	0·2 (−0·4 to 0·9)
Melanoma (C43)	0·3	0·1	0·1 (0·0 to 0·2)	0·6	0·5	0·2 (−0·0 to 0·4)	0·8	0·9	−0·2 (−0·6 to 0·2)
Breast (C50)	0·5	0·2	0·4 (0·3 to 0·4)	0·8	0·4	0·4 (0·3 to 0·5)	1·2	0·8	0·4 (0·1 to 0·6)
Uterus (C54–55)	0·5	0·2	0·3 (0·1 to 0·5)	0·9	0·5	0·4 (0·1 to 0·7)	1·2	1·0	0·1 (−0·6 to 0·8)
Prostate (C61)	0·4	0·2	0·2 (0·0 to 0·3)	0·9	0·5	0·4 (0·2 to 0·5)	1·3	0·8	0·5 (0·2 to 0·8)
Bladder (C67)	0·6	0·2	0·3 (0·1 to 0·5)	0·9	0·5	0·3 (0·2 to 0·5)	0·8	0·9	−0·1 (−0·4 to 0·2)
NHL (C82–85)	0·5	0·2	0·3 (0·1 to 0·5)	0·9	0·5	0·4 (0·1 to 0·7)	1·1	1·0	0·0 (−0·6 to 0·6)
Leukaemia (C91–95)	0·7	0·2	0·5 (0·1 to 0·8)	1·2	0·6	0·6 (0·2 to 1·0)	1·3	1·0	0·3 (−0·3 to 1·0)

Data are absolute incidence (from time of cancer diagnosis) of cardiovascular disease outcomes per 100 person-years (or equivalently risk [%] per year). Cancer codes from the International Classification of Diseases, version 10. Incidence in cancer survivors and expected incidence calculated by fitting flexible parametric survival models with exposure (cancer survivor *vs* control), covariates, and interaction between exposure and time since diagnosis and predicting incidence for exposed or unexposed at the mean value of covariates in cancer survivors. NHL=non-Hodgkin lymphoma.

In exploratory analyses in which cancer survivors were grouped according to treatment methods recorded in the registry data, we found evidence of a substantially elevated risk of venous thromboembolism in all cancer survivor groups compared with that of the general population, regardless of treatment. This risk was most pronounced in patients receiving chemotherapy. Chemotherapy use also seemed to be the key driver of heart failure or cardiomyopathy risk after breast cancer (appendix p 40). In sensitivity analyses using primary care data alone, effect estimates in the main Cox models were broadly similar, with slight movement towards the null for some exposure–outcome combinations, consistent with the known direction of bias resulting from non-differential misclassification.^18^ The use of more sensitive covariate definitions for hypertension and alcohol use made little difference to the effect estimates (appendix pp 41–44).

## Discussion

In this comprehensive assessment of the risk of cardiovascular disease in cancer survivors compared with that of general population controls, we found increased risks of cardiovascular diseases in the years after diagnosis of most site-specific cancers. We observed large increases in the risk of venous thromboembolism in survivors of 18 of 20 cancer types. This risk attenuated over time but remained elevated for at least 5 years after diagnosis for most cancer types. We also observed increased risks of heart failure or cardiomyopathy in survivors of ten of 20 site-specific cancer types, including greater than 50% increases in risk after haematological, oesophageal, lung, kidney, and ovarian cancers and increased risks of arrhythmia, pericarditis, coronary artery disease, stroke, and valvular heart disease for multiple cancer sites. Survivors of haematological malignancies had increased risks across all these outcomes, but the pattern of risks varied for other cancer sites. Adjustment for shared risk factors for cancer and cardiovascular disease had little effect on associations. Relative risks for heart failure or cardiomyopathy and venous thromboembolism associated with cancer survival were most pronounced in younger ages and in individuals with no previous cardiovascular disease or hypertension (ie, those with the lowest baseline risk); however, higher absolute effects were often observed in older individuals.

Our finding of substantial variability by cancer site of associations between cancer survival and subsequent cardiovascular disease is consistent with a US study^19^ comparing risks for a composite cardiovascular disease outcome between cohorts with site-specific cancers and controls without a history of cancer. This study showed significantly increased cardiovascular disease risks for breast and lung cancers, malignant melanoma, and non-Hodgkin lymphoma and an unexpected reduced risk for prostate cancer; no associations were found for the remaining nine cancers studied, including for bladder cancer.^19^ The composite outcome in the US study included ischaemic heart disease, heart failure or cardiomyopathy, and stroke. In our study, we analysed these three outcomes individually, and our results were consistent with the US study findings, except that we did not replicate the inverse association in survivors of prostate cancer, and we found the risk of all three outcomes to be increased after bladder cancer. This discrepancy might reflect differences in the profiles of patients with bladder cancer between the two countries, differences in treatment protocols,^20^ or chance variation.

Most previous studies have focused on survivors of a single, site-specific cancer, most commonly breast cancer (appendix pp 47–59). Although consistent findings exist of increased heart failure risk in patients after breast cancer, mainly driven by patients receiving anthracyclines, trastuzumab, or both,^19^, ^21^ the risks of coronary artery disease in this population are less clear. We found a lower risk of coronary artery disease in survivors of breast cancer compared with that of controls, in line with a US study^22^ that found reductions in myocardial infarction risk in this population. By contrast, a Danish study^11^ in people diagnosed at age 15–39 years observed a higher risk of myocardial infarction in survivors of breast cancer compared with that of controls, whereas most other studies^19^, ^21^, ^23^, ^24^, ^25^, ^26^ found no difference in either direction. A reduced risk of coronary artery disease in survivors of breast cancer might be driven by socioeconomic factors;^27^ these can vary by setting, with a higher socioeconomic level being associated with a higher risk of breast cancer in the UK. In our study, we matched by general practice, which would give some control for geographical area-based socioeconomic factors and, in fully adjusted models, we also included the Index of Multiple Deprivation, a patient postcode-based deprivation index; however, we were not able to adjust for socioeconomic status at the more granular household or individual level. Several studies^11^, ^28^, ^29^ have shown increased risks of venous thromboembolism in patients after breast cancer, including a study^30^ that found increased risks during and immediately after treatment. Strikingly, in our study, venous thromboembolism risk was elevated in patients for at least 5 years after diagnosis compared with that of controls with no history of cancer, even among patients with no record of receiving chemotherapy, suggesting long-term effects of the underlying malignancy, long-term endocrine therapies, or residual confounding by shared risk factors.

A major strength of our study was that the size and breadth of the linked data sources enabled us to investigate associations in granular detail. The importance of this is evident in the observed heterogeneity in the associations by cancer site and specific cardiovascular disease outcomes, showing that analyses of composite outcomes and all-cancer exposures can mask important variation. We had the data and statistical power to do a comprehensive analysis of medium-term and long-term cardiovascular disease risks for survivors of 20 site-specific cancers and to investigate effect modifications for the nine most common cancers and key cardiovascular disease outcomes. The use of consistent methodology across a wide range of cancers and outcomes gives confidence that the observed patterns of risk reflect real phenomena, rather than methodological artefacts. Multiple validation studies^31^, ^32^, ^33^ have established the strengths of using CPRD GOLD data linked to secondary care data sources to identify disease phenotypes, including for cancer and cardiovascular disease. We used multiple data linkages to maximise capture of cancer exposures and cardiovascular disease outcomes and to minimise misclassification. CPRD GOLD data have been shown to be representative of the general UK population on key demographics; therefore our results are generalisable to the UK and similar settings.^12^

Nonetheless, our study has important limitations. Although we could broadly categorise some groups of cancer survivors according to key treatment methods recorded, and thus begin to explore the role of treatment in driving cardiovascular disease risks, we had no information on specific chemotherapy drugs or doses received and no details on fields or doses of radiotherapy exposure. For several cancer sites, cancer registration data were too incomplete to be used at all. Where data were available, power was insufficient to assess whether the time course of cardiovascular disease in cancer survivors differed according to the initial treatments received. Cancer registry treatment data are most complete for treatments received during the first 6 months after diagnosis, with less information after this, and we had no reliable information on cancer recurrence, which could have been an important predictor of further cardiotoxic treatment and subsequent increased cardiovascular disease risk. Stage and grade data were not sufficiently complete across the study period to assess differences by severity of cancer at diagnosis. Our prescription data came from primary care alone and thus, we could not assess trends over time in hospital prescribing of drugs that might be given to mitigate chemotherapy-induced cardiotoxicities, such as anti-hypertensive drugs or dexrazoxane, in the context of increasing awareness of the cardiovascular risks in patients after cancer. The greater use of such cardioprotective drugs in patients with pre-existing cardiovascular disease might explain the lower HRs observed in that group for some outcomes. We did not have sufficiently detailed data to classify the stroke outcome as ischaemic or haemorrhagic, which could have been important given the observed heterogeneity of associations between cancer survival and different cardiovascular disease outcomes. Some individuals had missing BMI and smoking data; our complete-case analysis assumed that associations between cancer and cardiovascular disease, conditional on covariates, do not differ between included and excluded individuals,^34^ which we have no reason to doubt. We estimated associations between multiple cancer sites and cardiovascular disease outcomes, increasing the risk of false positive associations; therefore, individual effect estimates with wide CIs that are close to the null should be interpreted cautiously. Finally, we did not have reliable information on potentially important variables including dyslipidaemia, family history of cardiovascular disease, ethnicity, diet, alcohol intake, exercise, and age at menopause (for gynaecological cancers).

The causes of increased cardiovascular disease risks in these patient groups are likely to be varied, but our analyses suggest that cancer treatments, particularly chemotherapy, are likely to play a more prominent role than shared risk factors such as smoking and excess weight. Surgery-associated phenomena and mechanisms directly related to cancer biology might also play a part, for example in the increased risk of stroke in survivors of central nervous system cancers. The best-known cardiotoxicity related to cancer treatment is that of anthracycline chemotherapy drugs, which have been found to substantially increase risk of reduced left ventricular ejection fraction and subsequent clinical heart failure.^35^ Trastuzumab is also known to cause left ventricular dysfunction and is likely to have driven part of the observed elevated heart failure risk in patients after breast cancer.^36^ Chest radiotherapy involving the heart has been shown to increase risks of coronary artery disease in a dose-dependent manner,^37^ which might explain the increased risks of this outcome in patients after oesophageal cancer, lung cancer, and haematological malignancies that might require chest irradiation. Platinum-containing chemotherapies used in lung, oesophageal, and other cancers might also play a role.^38^ However, more broadly, insufficient evidence exists on the specific long-term cardiotoxicities of modern systemic anti-cancer drugs, radiotherapy with modern techniques, and the interactions between them. Registration of anti-cancer therapies is becoming more widespread, with potential for linkage to other routinely collected data; this will begin to provide crucial long-term data to fill this evidence gap and enable greater delineation of risk drivers in follow-up studies.

Our findings might have several implications for patient management to improve outcomes. The relevance of traditional cardiovascular disease risk prediction and risk minimisation for patients living after a cancer diagnosis is unclear. Prediction models of cardiovascular disease risk used in general practice typically do not include outcomes such as heart failure and venous thromboembolism and do not include cancer as a predictor. Additionally, these models generally ignore competing risks,^39^ which are likely to be important in a cancer survival setting: our analyses of cumulative incidence showed that cardiovascular disease burden might be influenced by both cancer mortality and cardiovascular disease risk. For example, heart failure risk was considerably elevated in survivors of lung cancer compared with that of general population controls, but a high incidence of lung cancer mortality meant that the 5-year cumulative incidence of heart failure was actually lower in our lung cancer cohort compared with that in general population controls, after we accounted for the competing risk of non-cardiovascular disease death. Beyond more tailored risk prediction modelling, screening for biomarkers or with routine imaging might be needed to identify cancer survivors at high risk of a range of cardiovascular diseases. Prevention at early stages is a priority because the prognosis for cancer survivors diagnosed with cardiovascular disease is particularly poor.^19^ Hopes have been placed on the use of myocardial strain, troponin, and natriuretic peptide as biomarkers that could identify developing heart function problems at an early stage.^40^ One difficulty with managing post-cancer risk of cardiovascular disease has been that the long-term follow-up is typically done by cancer specialists, leading to insufficient focus on potential cardiovascular disease sequelae. Cardio-oncology clinics that bring together oncologists, cardiologists, and specialist nurses are starting to appear and will help to address this growing clinical problem.^40^, ^41^ Primary care physicians are also key for early prevention, and a pressing need exists to raise awareness among GPs of the increased cardiovascular disease risks in cancer survivors. In a survey of English GPs, only about half reported receiving cancer treatment summaries and only a fifth reported considering cancer in relation to cardiovascular health.^42^ This is corroborated by a study published in 2018,^43^ in which we showed that cancer survivors were no more likely to receive cardioprotective medications than controls without a history of cancer, with low uptake in both groups. Clearly, more and better education and awareness is needed among GPs, other clinicians, and patients, so that preventive strategies can be considered and implemented, covering the extended time course during which our results suggest that cardiovascular disease risk might be elevated. Available information for clinicians is insufficient, and the development of comprehensive, evidence-based, national level guidance on cardiovascular considerations in cancer survival care could be prioritised to help optimise the care of this patient group.

In conclusion, survivors of most site-specific cancers had higher risks of cardiovascular disease compared with those of people without diagnosed cancer, with patterns of risk varying by cancer site and by specific cardiovascular disease outcome. Strategies to minimise and manage cardiovascular risk are needed for the growing population of cancer survivors.
