# Supplementary material for: Medium and long-term risks of specific cardiovascular diseases in survivors of 20 adult cancers: a population-based cohort study using multiple linked UK electronic health records databases
Source: Lancet. 2019 Sep 21;394(10203):1041–54. doi: 10.1016/S0140-6736(19)31674-5 (PMC6857444; doi:10.1016/S0140-6736(19)31674-5)
Supplement: Supplementary appendix [file mmc1.pdf]

THE LANCET

Supplementary appendix

This appendix formed part of the original submission and has been peer reviewed. We post it as supplied by the authors.

Supplement to: Strongman H, Gadd S, Matthews A, et al. Medium and long-term risks of specific cardiovascular diseases in survivors of 20 adult cancers: a population-based cohort study using multiple linked UK electronic health records databases. *Lancet* 2019; published online Aug 20. http://dx.doi.org/10.1016/S0140-6736(19)31674-5.

Supplementary appendix

Supplement to: Strongman H, Gadd S, Matthews A, Mansfield KE, Stanway S, Lyon AR, dos-Santos-Silva I, Smeeth L, Bhaskaran K. Long-term risks of specific cardiovascular diseases among 108,215 survivors of 20 adult cancers: population-based cohort study using multiple linked UK electronic health records databases.

Table of contents

	Page
**Part A - additional methods**	
Table A1. Variable definitions	4-6
Figure A1. Directed acyclic graph of the assumed association between cancer and its treatment; cardiovascular disease; and shared risk factors and demographic characteristics	7
**Part B – additional results**	
Figure B1: Flow diagrams describing the creation of the cancer survivor groups and reasons for exclusions	8
Table B1-B20: Characteristics of site-specific cancer survivor and matched control study populations	9-28
Table B1: Oral cancer (ICD10 C00-8)	9
Table B2: Oesophageal cancer (ICD10 C15)	10
Table B3: Stomach cancer (ICD10 C16)	11
Table B4: Colorectal cancer (ICD10 C18-20)	12
Table B5: Liver cancer (ICD10 C22)	13
Table B6: Pancreas cancer (ICD10 C25)	14
Table B7: Lung cancer (ICD10 C34)	15
Table B8: Malignant melanoma (ICD10 C43)	16
Table B9: Breast cancer (ICD10 C50)	17
Table B10: Cervical cancer (ICD10 C53)	18
Table B11: Uterus cancer (ICD10 C54-55)	19
Table B12: Ovarian cancer (ICD10 C56)	20

Table B13: Prostate cancer (ICD10 C61)	21
Table B14: Kidney cancer (ICD10 C64)	22
Table B15: Bladder cancer (ICD10 C67)	23
Table B16: Brain / Central Nervous System cancer (ICD10 C71-72)	24
Table B17: Thyroid cancer (ICD10 C73)	25
Table B18: Non-Hodgkin Lymphoma (ICD10 C82-85)	26
Table B19: Multiple Myeloma (ICD10 C90)	27
Table B20: Leukaemia (ICD10 C91-95)	28
Figure B2: Absolute and relative risk of cardiovascular disease in cancer survivors compared to general population controls	29-30
A-D) Coronary artery disease component outcomes	29
E-H) Venous thromboembolism component outcomes and heart failure/cardiomyopathy	30
Figure B3: Associations between site-specific cancer survivorship and specific CVD outcomes, stratified by other factors	31-35
A) Coronary artery disease	31
B) Stroke	32
C) Heart failure/cardiomyopathy	33
D) Arrhythmia	34
E) Venous thromboembolism	35
Figure B4: Observed and expected absolute incidences of cardiovascular outcomes over time since diagnosis, by age group	36-38
Table B21: Cumulative incidence at 5 years of specific cardiovascular outcomes among cancer survivors and controls, in the presence of competing risks of other causes of death	39
Figure B5: Associations between site-specific cancer survivorship and specific CVD outcomes, stratified by receipt of chemotherapy/radiotherapy	40
Figure B6: Relative risk of cardiovascular disease in cancer survivors compared to controls from sensitivity analyses	41-44
A-B) Coronary artery disease and arrhythmia	41
C-D) Heart failure/cardiomyopathy and stroke	42
E-F) Peripheral vascular disease and venous thromboembolism	43
G-H) Pericarditis and valvular heart disease	44

**Part C – systematic review of previous studies comparing specific CVD outcomes in site-specific cancer survivors vs non-cancer or general population controls**	45
Methods for systematic review	45
Figure C1: Review of previous studies: flow chart of the systematic review search process	46
Table C1: Review of previous studies - study characteristics	47-51
Figure C2: Risk of CVD outcomes in cancer survivors vs controls in previous studies and present study	52-59
A) Arrhythmia	52
B) Coronary artery disease	53
C) Heart failure/cardiomyopathy	54
D) Pericarditis	55
E) Peripheral vascular disease	56
F) Stroke/cerebrovascular disease	57
G) Ventricular heart disease	58
H) Venous thromboembolism	59
References for the systematic review	60-61
**Part D – original study protocol**	62
Protocol: The risk of cardiovascular disease among survivors of site-specific adult cancers	63-76
Deviations from approved protocol with justification	77

Part A - Additional Methods

Table A1: Variable definition spreadsheet

Covariate	Definition and use	Derivation
Demographic		
Age	matching variable (=/- 3 years) and categorical potential effect modifier (18-59, 60-79, >=80)	01/07/birthyear (Exact date of birth not collected by CPRD to maintain the de-identified nature of the data)
Sex	Sex (male, female); matching variable and potential effect modifier	
Patient area based deprivation	Ordered categorical covariate and potential effect modifier used as a proxy for socio-economic status (1 least deprived, 2, 3, 4, 5 most deprived)	Index of multiple deprivation (IMD) quintile identified by CPRD through third party linkage to patient postcode
Practice area based deprivation	Replaces patient area based deprivation in sensitivity analysis using primary care data only	IMD quintile identified by CPRD through linkage to practice postcode
Behavioural / lifestyle		
Smoking status	Categorical shared risk factor and potential effect modifier (non-smoker, smoker, ex-smoker)<br><br>Subjects with missing data excluded from all models	Nearest structured or Read coded record of smoking status to baseline entered at any time before this date or up to 30 days after. Non-smokers recategorised as ex-smoker if there was a previous record of smoking
Current or previous heavy drinker	Binary shared risk factor (use alcohol status code list)	Read-coded record of problem / drinking at any time prior to baseline
Alcohol status and level	Categorical shared risk factor replacing 'current or previous heavy drinker' in alcohol sensitivity models [non drinker, ex-drinker, current drinker (light, moderate, heavy, amount unknown), missing]<br><br>Subjects with missing data excluded from alcohol sensitivity models only	Nearest Read-coded or structured record of alcohol status to baseline entered at any time before this date or up to 30 days after. Non-drinkers recategorised as ex-drinkers if there was a previous record of drinking. Level of current consumption based on information in the Read codes or structured record [Light (1-14 units), moderate (15/42 units), heavy (>=43 units)]
Cardio-metabolic disease		
Body Mass Index (BMI)	Shared risk factor (3 knotted restricted cubic spline)<br><br>Categorical potential effect modifier (underweight, healthy weight, overweight, obese) based on WHO categories	Calculated from nearest structured record of weight and height entered at any time before baseline or up to 30 days after. Implausible measurements excluded (https://www.ncbi.nlm.nih.gov/pubmed/24038008)

Covariate	Definition and use	Derivation
Previous diabetes	Initially defined as categorical variable differentiating between type 1 and type 2 diabetes (see tables in Appendix).<br><br>Collapsed into binary shared risk factor to improve regression model stability / likelihood ratios	specific T1DM or T2DM Read code with no contradictions prior to baseline OR<br>non-specific code and insulin as first therapy (T1D) OR<br>non-specific code and met, other or no therapy (T2D)
hypertension in year following baseline	Binary shared risk factor and potential effect modifier. In main analysis, subjects without as least two blood pressure measures, including one in the 365 days prior to baseline, are classified as normotensive. In the hypertension sensitivity analysis, these subjects are excluded from the analysis.	Two most recent valid* blood pressure measurements high (systolic >= 140 or diastolic >=90), including one within 365 days prior to baseline. *Diastolic values <30 and >200, and sytolic values <40 and > 240 discounted; smallest value taken if more than one on a single day
(other) cardiovascular disease	Binary shared risk factor and potential effect model	Record of cardiovascular disease prior to baseline (Read codes in CPRD GOLD or ICD-10 codes in HES). For each analysis, subjects with the CVD (e.g. coronary artery disease) prior to index are excluded. This variable therefore only includes other CVDs
Cardiovascular treatment related		
Statin use	Binary shared risk factor	At least two statin precriptions recorded in year before baseline
Beta-blockers	Binary shared risk factor	At least two beta blocker precriptions recorded in year before baseline
ACE inhibitors	Binary shared risk factor	At least two ACE-inhibitor precriptions recorded in year before baseline
ARBs	Binary shared risk factor	At least two ARB precriptions recorded in year before baseline
NSAIDs including aspirin	Binary shared risk factor	At least two NSAID precriptions recorded in year before baseline
Hormone related risk factors		
Hormone replacement therapy	Categorical shared risk factor for female organ cancers only (never, HRT in year before baseline, HRT stopped prior to baseline year)	At least one record of hormone replacement in the last year or ever prior to baseline (past use)
Hysterectomy	Binary shared risk factor for female organ cancers only	Read-coded record of hysterectomy at any time prior to baseline
Chronic inflammatory / autoimmune diseases		
rheumatoid arthritis	Binary shared risk factor in initial analyses. Excluded as rarity destabilises regression models.	At least one Read coded record of rheumatoid arthritis at any time prior to baseline
Lupus / SLE	Binary shared risk factor in initial analyses. Excluded as rarity destabilises regression models.	At least one Read coded record of lupus/SLE at any time prior to baseline

Covariate	Definition and use	Derivation
Sclerosis	Binary shared risk factor for leukaemia and lung cancer in initial analyses. Excluded from leukaemia analysis as rarity destabilises regression models.	At least one Read coded record of sclerosis at any time prior to baseline
COPD	Binary shared risk factor for lung cancer	At least one Read coded record of COPD at any time prior to baseline
Other risk factors		
migraines	Binary shared risk factor	At least one Read coded record of migraine at any time prior to baseline
severe mental illness (schizophrenia, bipolar, moderate / severe depression)	Binary shared risk factor in initial analyses. Excluded as rarity destabilises regression models.	At least one Read coded record of SMI at any time prior to baseline
chronic kidney disease	Initially defined as categorical variable differentiating between CKD stages 3a, 3b, 4 and 5 (see tables in Appendix).<br><br>Collapsed into binary shared risk factor (no CKD, CKD) to improve regression model likelihood ratios	Most recent cleaned* serum creatine value recorded at any time prior to baseline used to calculate eGFR using formula (SCrx0.95)/88.4. Categories: no CKD >=60 mL/min, stage 3a 45-<60 mL/min, stage 3b 30-<45 mL/min, stage 4 15-<30 v, stage 5 <15 mL/min<br><br>*only include serum creatinine records with both structured data area / read code indicative of serum creatinine test, values >=20, <3000
Chronic liver disease including HBV / HCV	Binary shared risk factor for liver cancer only	At least one Read coded record of SMI at any time prior to baseline
Immunosuppression	Binary shared risk factor for NHL only	At least one Read coded record of HIV or organ transplantation, or at least one prescription record for immunosuppression drugs ever prior to baseline
Obstructive sleep apnoea	Binary shared risk factor in initial analyses. Excluded as rarity destabilises regression models and it can be difficult to differentiate between OSA as a shared risk factor and part of the causal pathway.	At least one Read coded record of obstructive sleep apnoea at any time prior to baseline

Figure A1: Directed acyclic graph of the assumed association between cancer and its treatment; cardiovascular disease; and shared risk factors and demographic characteristics

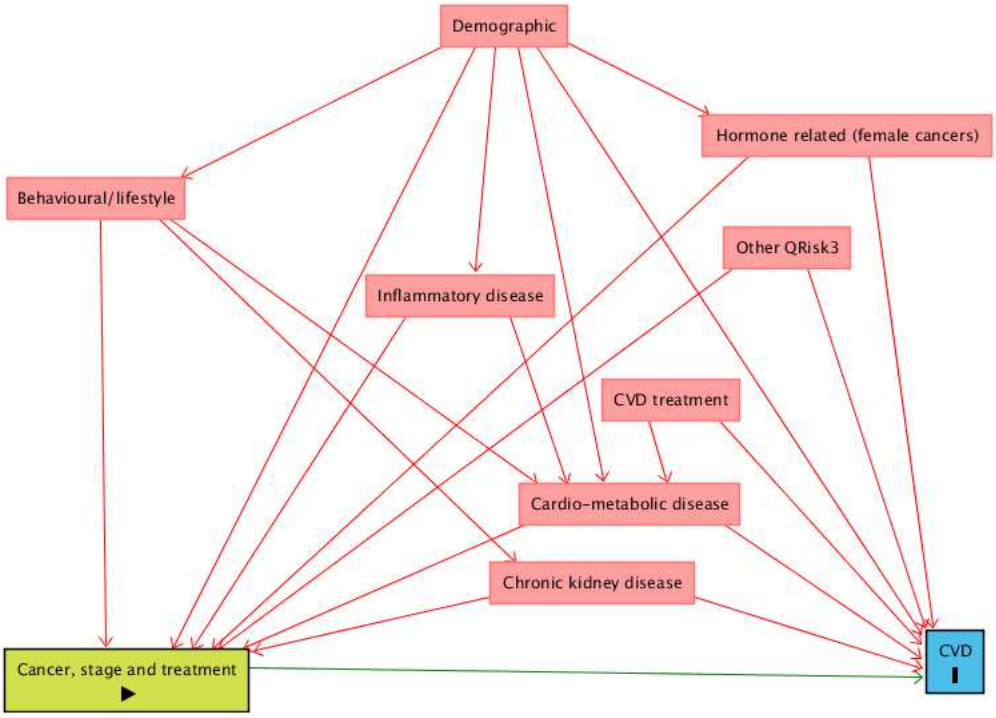

Part B - Additional Results

Figure B1: Flow diagram describing the creation of the cancer survivor groups and reasons for exclusions for the primary care only cohort (sensitivity analysis)

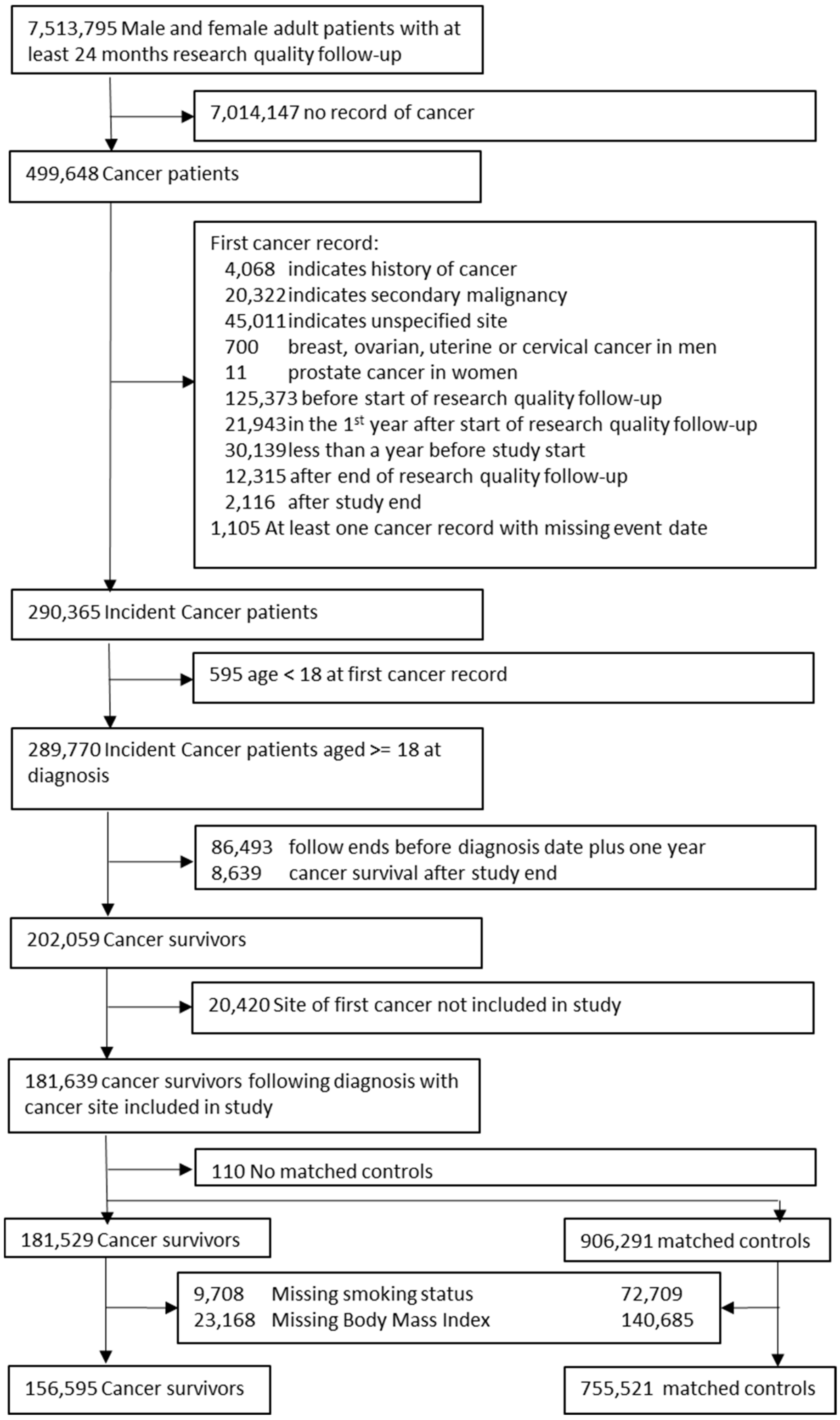

Table B1: Characteristics of ORAL cancer (ICD10 C00-8) survivors and matched controls

	Cancer survivors	Controls
**N**	1,584	7,787
**Person-years from cancer the matched diagnosis date (baseline) to end of follow-up I**		
Mean (SD)	5.5 (4.1)	6.4 (4.3)
Median (IQR)	4.4 (2.5, 7.5)	5.3 (3.0, 8.7)
Range	1.0-24.5	1.0-24.6
**Total person-years included* (millions)**	0.007	0.042
**Age (years) M**		
Mean (SD)	63.3 (13.3)	63.7 (13.2)
Median (IQR)	63.0 (54.0, 73.0)	64.0 (55.0, 73.0)
**Age (years) I**		
18-59	618 (39.0)	2,949 (37.9)
60-79	775 (48.9)	3,856 (49.5)
>=80	191 (12.1)	982 (12.6)
**Sex M,I**		
male	923 (58.3)	4,518 (58.0)
female	661 (41.7)	3,269 (42.0)
**Index of multiple deprivation quintile (patient area) SRF,I**		
1 (least deprived)	341 (21.5)	1,895 (24.3)
2	345 (21.8)	1,785 (22.9)
3	310 (19.6)	1,628 (20.9)
4	302 (19.1)	1,372 (17.6)
5 (most deprived)	286 (18.1)	1,107 (14.2)
**Year of cancer diagnosis M,I**		
1989 to 1998	157 (9.9)	737 (9.5)
1999 to 2003	330 (20.8)	1,621 (20.8)
2004 to 2008	535 (33.8)	2,632 (33.8)
2009 to 2014	562 (35.5)	2,797 (35.9)
**Smoking status SRF,I**		
non-smoker	511 (32.3)	3,584 (46.0)
current smoker	587 (37.1)	1,522 (19.5)
ex-smoker	486 (30.7)	2,681 (34.4)
**Heavy drinker SRF**	143 (9.0)	423 (5.4)
**Body Mass Index SRF(spline),I(categorical)**		
underweight	56 (3.5)	96 (1.2)
healthy weight	628 (39.6)	2,733 (35.1)
overweight	603 (38.1)	3,168 (40.7)
obese	297 (18.8)	1,790 (23.0)
**Diabetes SRF**	149 (9.4)	812 (10.4)
**Hypertension SRF,I**	347 (21.9)	1,634 (21.0)
**Previous cardiovascular disease SRF,I**	410 (25.9)	2,048 (26.3)
**Cardiovascular treatments SRF**		
**Statins**	341 (21.5)	1,731 (22.2)
**Beta blockers**	236 (14.9)	1,149 (14.8)
**Angiotensin converting enzyme (ACE) inhibitors**	262 (16.5)	1,304 (16.7)
**Angiotensin II receptor blockers (ARBs)**	95 (6.0)	480 (6.2)
**Non steroidal anti-inflammatory drugs (NSAIDs)**	140 (8.8)	760 (9.8)
**Previous migraine SRF**	75 (4.7)	474 (6.1)
**Chronic kidney disease SRF**	164 (10.4)	900 (11.6)

Data are n (%) and represent status at the matched diagnosis date (baseline) unless stated otherwise. * index date to end of follow-up. M Matching variable, SRF Shared Risk Factor, I Interactions investigated.

Table B2: Characteristics of OESOPHAGEAL cancer (ICD10 C15) survivors and matched controls

	Cancer survivors	Controls
**N**	1,794	8,555
**Person-years from cancer the matched diagnosis date (baseline) to end of follow-up I**		
Mean (SD)	3.6 (3.3)	6.2 (3.9)
Median (IQR)	2.2 (1.4, 4.3)	5.3 (3.1, 8.5)
Range	1.0-23.7	1.0-24.2
**Total person-years included* (millions)**	0.005	0.044
**Age (years) M**		
Mean (SD)	67.9 (11.1)	68.1 (11.0)
Median (IQR)	68.0 (60.0, 76.0)	68.0 (61.0, 76.0)
**Age (years) I**		
18-59	401 (22.4)	1,858 (21.7)
60-79	1,116 (62.2)	5,373 (62.8)
>=80	277 (15.4)	1,324 (15.5)
**Sex M,I**		
male	1,230 (68.6)	5,770 (67.4)
female	564 (31.4)	2,785 (32.6)
**Index of multiple deprivation quintile (patient area) SRF,I**		
1 (least deprived)	427 (23.8)	2,060 (24.1)
2	401 (22.4)	2,044 (23.9)
3	366 (20.4)	1,757 (20.5)
4	313 (17.4)	1,465 (17.1)
5 (most deprived)	287 (16.0)	1,229 (14.4)
**Year of cancer diagnosis M,I**		
1989 to 1998	138 (7.7)	625 (7.3)
1999 to 2003	427 (23.8)	2,021 (23.6)
2004 to 2008	629 (35.1)	2,988 (34.9)
2009 to 2014	600 (33.4)	2,921 (34.1)
**Smoking status SRF,I**		
non-smoker	544 (30.3)	3,649 (42.7)
current smoker	461 (25.7)	1,498 (17.5)
ex-smoker	789 (44.0)	3,408 (39.8)
**Heavy drinker SRF**	56 (3.1)	455 (5.3)
**Body Mass Index SRF(spline),I(categorical)**		
underweight	50 (2.8)	135 (1.6)
healthy weight	624 (34.8)	2,995 (35.0)
overweight	729 (40.6)	3,561 (41.6)
obese	391 (21.8)	1,864 (21.8)
**Diabetes SRF**	254 (14.2)	1,017 (11.9)
**Hypertension SRF,I**	461 (25.7)	2,045 (23.9)
**Previous cardiovascular disease SRF,I**	624 (34.8)	2,728 (31.9)
**Cardiovascular treatments SRF**		
**Statins**	535 (29.8)	2,315 (27.1)
**Beta blockers**	318 (17.7)	1,543 (18.0)
**Angiotensin converting enzyme (ACE) inhibitors**	394 (22.0)	1,715 (20.0)
**Angiotensin II receptor blockers (ARBs)**	133 (7.4)	582 (6.8)
**Non steroidal anti-inflammatory drugs (NSAIDs)**	157 (8.8)	874 (10.2)
**Previous migraine SRF**	95 (5.3)	416 (4.9)
**Chronic kidney disease SRF**	266 (14.8)	1,228 (14.4)

Data are n (%) and represent status at the matched diagnosis date (baseline) unless stated otherwise. * index date to end of follow-up. M Matching variable, SRF Shared Risk Factor, I Interactions investigated.

Table B3: Characteristics of STOMACH cancer (ICD10 C16) survivors and matched controls

	Cancer survivors	Controls
**N**	1,507	7,135
**Person-years from cancer the matched diagnosis date (baseline) to end of follow-up I**		
Mean (SD)	4.0 (3.5)	6.4 (4.2)
Median (IQR)	2.6 (1.6, 5.4)	5.5 (3.2, 8.7)
Range	1.0-23.1	1.0-23.1
**Total person-years included* (millions)**	0.005	0.039
**Age (years) M**		
Mean (SD)	70.5 (11.4)	70.3 (11.4)
Median (IQR)	72.0 (64.0, 78.0)	72.0 (64.0, 78.0)
**Age (years) I**		
18-59	248 (16.5)	1,187 (16.6)
60-79	942 (62.5)	4,465 (62.6)
>=80	317 (21.0)	1,483 (20.8)
**Sex M,I**		
male	972 (64.5)	4,589 (64.3)
female	535 (35.5)	2,546 (35.7)
**Index of multiple deprivation quintile (patient area) SRF,I**		
1 (least deprived)	301 (20.0)	1,522 (21.3)
2	294 (19.5)	1,552 (21.8)
3	338 (22.4)	1,487 (20.8)
4	295 (19.6)	1,314 (18.4)
5 (most deprived)	279 (18.5)	1,260 (17.7)
**Year of cancer diagnosis M,I**		
1989 to 1998	187 (12.4)	860 (12.1)
1999 to 2003	378 (25.1)	1,801 (25.2)
2004 to 2008	512 (34.0)	2,425 (34.0)
2009 to 2014	430 (28.5)	2,049 (28.7)
**Smoking status SRF,I**		
non-smoker	538 (35.7)	3,087 (43.3)
current smoker	306 (20.3)	1,158 (16.2)
ex-smoker	663 (44.0)	2,890 (40.5)
**Heavy drinker SRF**	35 (2.3)	366 (5.1)
**Body Mass Index SRF(spline),I(categorical)**		
underweight	35 (2.3)	109 (1.5)
healthy weight	550 (36.5)	2,497 (35.0)
overweight	628 (41.7)	3,003 (42.1)
obese	294 (19.5)	1,526 (21.4)
**Diabetes SRF**	223 (14.8)	923 (12.9)
**Hypertension SRF,I**	384 (25.5)	1,826 (25.6)
**Previous cardiovascular disease SRF,I**	604 (40.1)	2,513 (35.2)
**Cardiovascular treatments SRF**		
**Statins**	447 (29.7)	2,046 (28.7)
**Beta blockers**	287 (19.0)	1,349 (18.9)
**Angiotensin converting enzyme (ACE) inhibitors**	337 (22.4)	1,490 (20.9)
**Angiotensin II receptor blockers (ARBs)**	127 (8.4)	540 (7.6)
**Non steroidal anti-inflammatory drugs (NSAIDs)**	146 (9.7)	796 (11.2)
**Previous migraine SRF**	73 (4.8)	348 (4.9)
**Chronic kidney disease SRF**	277 (18.4)	1,281 (18.0)

Data are n (%) and represent status at the matched diagnosis date (baseline) unless stated otherwise. * index date to end of follow-up. M Matching variable, SRF Shared Risk Factor, I Interactions investigated.

Table B4: Characteristics of COLORECTAL cancer (ICD10 C18-20) survivors and matched controls

	Cancer survivors	Controls
**N**	14,216	68,776
**Person-years from cancer the matched diagnosis date (baseline) to end of follow-up I**		
Mean (SD)	5.5 (4.0)	6.3 (4.1)
Median (IQR)	4.3 (2.3, 7.5)	5.4 (3.1, 8.6)
Range	1.0-25.1	1.0-25.9
**Total person-years included* (millions)**	0.063	0.366
**Age (years) M**		
Mean (SD)	69.4 (11.6)	69.4 (11.5)
Median (IQR)	70.0 (62.0, 78.0)	70.0 (62.0, 78.0)
**Age (years) I**		
18-59	2,686 (18.9)	12,900 (18.8)
60-79	8,672 (61.0)	42,241 (61.4)
>=80	2,858 (20.1)	13,635 (19.8)
**Sex M,I**		
male	7,807 (54.9)	37,591 (54.7)
female	6,409 (45.1)	31,185 (45.3)
**Index of multiple deprivation quintile (patient area) SRF,I**		
1 (least deprived)	3,498 (24.6)	17,046 (24.8)
2	3,360 (23.6)	16,063 (23.4)
3	3,063 (21.5)	14,725 (21.4)
4	2,389 (16.8)	11,760 (17.1)
5 (most deprived)	1,906 (13.4)	9,182 (13.4)
**Year of cancer diagnosis M,I**		
1989 to 1998	1,552 (10.9)	7,326 (10.7)
1999 to 2003	3,244 (22.8)	15,645 (22.7)
2004 to 2008	5,001 (35.2)	24,145 (35.1)
2009 to 2014	4,419 (31.1)	21,660 (31.5)
**Smoking status SRF,I**		
non-smoker	6,241 (43.9)	31,383 (45.6)
current smoker	2,062 (14.5)	11,184 (16.3)
ex-smoker	5,913 (41.6)	26,209 (38.1)
**Heavy drinker SRF**	491 (3.5)	3,112 (4.5)
**Body Mass Index SRF(spline),I(categorical)**		
underweight	266 (1.9)	1,081 (1.6)
healthy weight	5,159 (36.3)	25,044 (36.4)
overweight	5,776 (40.6)	27,675 (40.2)
obese	3,015 (21.2)	14,976 (21.8)
**Diabetes SRF**	1,882 (13.2)	8,104 (11.8)
**Hypertension SRF,I**	3,498 (24.6)	17,302 (25.2)
**Previous cardiovascular disease SRF,I**	4,612 (32.4)	22,220 (32.3)
**Cardiovascular treatments SRF**		
**Statins**	3,756 (26.4)	18,058 (26.3)
**Beta blockers**	2,575 (18.1)	12,525 (18.2)
**Angiotensin converting enzyme (ACE) inhibitors**	2,844 (20.0)	13,983 (20.3)
**Angiotensin II receptor blockers (ARBs)**	1,061 (7.5)	5,122 (7.4)
**Non steroidal anti-inflammatory drugs (NSAIDs)**	1,213 (8.5)	7,074 (10.3)
**Previous migraine SRF**	676 (4.8)	3,854 (5.6)
**Chronic kidney disease SRF**	2,445 (17.2)	11,369 (16.5)

Data are n (%) and represent status at the matched diagnosis date (baseline) unless stated otherwise. * index date to end of follow-up. M Matching variable, SRF Shared Risk Factor, I Interactions investigated.

Table B5: Characteristics of LIVER cancer (ICD10 C22) survivors and matched controls

	Cancer survivors	Controls
N	554	2,643
**Person-years from cancer the matched diagnosis date (baseline) to end of follow-up I**		
Mean (SD)	3.1 (2.6)	5.5 (3.5)
Median (IQR)	2.1 (1.4, 3.5)	4.8 (2.9, 7.3)
Range	1.0-18.3	1.0-21.5
**Total person-years included* (millions)**	0.001	0.012
**Age (years) M**		
Mean (SD)	66.3 (12.7)	66.7 (12.3)
Median (IQR)	66.0 (59.0, 76.0)	67.0 (59.0, 76.0)
**Age (years) I**		
18-59	155 (28.0)	705 (26.7)
60-79	308 (55.6)	1,511 (57.2)
>=80	91 (16.4)	427 (16.2)
**Sex M,I**		
male	348 (62.8)	1,627 (61.6)
female	206 (37.2)	1,016 (38.4)
**Index of multiple deprivation quintile (patient area) SRF,I**		
1 (least deprived)	125 (22.6)	622 (23.5)
2	113 (20.4)	591 (22.4)
3	115 (20.8)	592 (22.4)
4	99 (17.9)	465 (17.6)
5 (most deprived)	102 (18.4)	373 (14.1)
**Year of cancer diagnosis M,I**		
1989 to 1998	28 (5.1)	126 (4.8)
1999 to 2003	92 (16.6)	434 (16.4)
2004 to 2008	192 (34.7)	900 (34.1)
2009 to 2014	242 (43.7)	1,183 (44.8)
**Smoking status SRF,I**		
non-smoker	208 (37.5)	1,132 (42.8)
current smoker	112 (20.2)	432 (16.3)
ex-smoker	234 (42.2)	1,079 (40.8)
**Heavy drinker SRF**	26 (4.7)	136 (5.1)
**Body Mass Index SRF(spline),I(categorical)**		
underweight	8 (1.4)	37 (1.4)
healthy weight	194 (35.0)	885 (33.5)
overweight	203 (36.6)	1,086 (41.1)
obese	149 (26.9)	635 (24.0)
**Diabetes SRF**	151 (27.3)	332 (12.6)
**Hypertension SRF,I**	123 (22.2)	538 (20.4)
**Previous cardiovascular disease SRF,I**	210 (37.9)	800 (30.3)
**Cardiovascular treatments SRF**		
**Statins**	158 (28.5)	744 (28.1)
**Beta blockers**	147 (26.5)	407 (15.4)
**Angiotensin converting enzyme (ACE) inhibitors**	134 (24.2)	543 (20.5)
**Angiotensin II receptor blockers (ARBs)**	58 (10.5)	208 (7.9)
**Non steroidal anti-inflammatory drugs (NSAIDs)**	49 (8.8)	278 (10.5)
**Previous migraine SRF**	33 (6.0)	155 (5.9)
**Chronic kidney disease SRF**	87 (15.7)	362 (13.7)
**Previous chronic liver disease**	120 (21.7)	48 (1.8)

Data are n (%) and represent status at the matched diagnosis date (baseline) unless stated otherwise. * index date to end of follow-up. M Matching variable, SRF Shared Risk Factor, I Interactions investigated.

Table B6: Characteristics of PANCREAS cancer (ICD10 C25) survivors and matched controls

	Cancer survivors	Controls
**N**	864	4,089
**Person-years from cancer the matched diagnosis date (baseline) to end of follow-up I**		
Mean (SD)	3.3 (3.2)	6.0 (3.8)
Median (IQR)	1.9 (1.3, 3.9)	5.2 (3.0, 8.1)
Range	1.0-20.8	1.0-22.9
**Total person-years included* (millions)**	0.002	0.020
**Age (years) M**		
Mean (SD)	67.0 (12.5)	67.2 (12.2)
Median (IQR)	68.0 (59.0, 76.0)	69.0 (59.0, 76.0)
**Age (years) I**		
18-59	225 (26.0)	1,033 (25.3)
60-79	501 (58.0)	2,416 (59.1)
>=80	138 (16.0)	640 (15.7)
**Sex M,I**		
male	416 (48.1)	1,944 (47.5)
female	448 (51.9)	2,145 (52.5)
**Index of multiple deprivation quintile (patient area) SRF,I**		
1 (least deprived)	224 (25.9)	1,043 (25.5)
2	211 (24.4)	1,035 (25.3)
3	191 (22.1)	855 (20.9)
4	140 (16.2)	669 (16.4)
5 (most deprived)	98 (11.3)	487 (11.9)
**Year of cancer diagnosis M,I**		
1989 to 1998	63 (7.3)	257 (6.3)
1999 to 2003	167 (19.3)	818 (20.0)
2004 to 2008	314 (36.3)	1,451 (35.5)
2009 to 2014	320 (37.0)	1,563 (38.2)
**Smoking status SRF,I**		
non-smoker	337 (39.0)	1,912 (46.8)
current smoker	190 (22.0)	661 (16.2)
ex-smoker	337 (39.0)	1,516 (37.1)
**Heavy drinker SRF**	20 (2.3)	212 (5.2)
**Body Mass Index SRF(spline),I(categorical)**		
underweight	24 (2.8)	64 (1.6)
healthy weight	358 (41.4)	1,526 (37.3)
overweight	322 (37.3)	1,572 (38.4)
obese	160 (18.5)	927 (22.7)
**Diabetes SRF**	210 (24.3)	466 (11.4)
**Hypertension SRF,I**	183 (21.2)	883 (21.6)
**Previous cardiovascular disease SRF,I**	286 (33.1)	1,198 (29.3)
**Cardiovascular treatments SRF**		
**Statins**	263 (30.4)	1,059 (25.9)
**Beta blockers**	163 (18.9)	677 (16.6)
**Angiotensin converting enzyme (ACE) inhibitors**	183 (21.2)	769 (18.8)
**Angiotensin II receptor blockers (ARBs)**	65 (7.5)	326 (8.0)
**Non steroidal anti-inflammatory drugs (NSAIDs)**	98 (11.3)	427 (10.4)
**Previous migraine SRF**	48 (5.6)	273 (6.7)
**Chronic kidney disease SRF**	135 (15.6)	613 (15.0)

Data are n (%) and represent status at the matched diagnosis date (baseline) unless stated otherwise. * index date to end of follow-up. M Matching variable, SRF Shared Risk Factor, I Interactions investigated.

Table B7: Characteristics of LUNG cancer (ICD10 C34) survivors and matched controls

	Cancer survivors	Controls
**N**	5,369	26,116
**Person-years from cancer the matched diagnosis date (baseline) to end of follow-up I**		
Mean (SD)	3.2 (3.0)	6.2 (4.1)
Median (IQR)	2.1 (1.4, 3.8)	5.2 (3.0, 8.6)
Range	1.0-24.1	1.0-24.6
**Total person-years included* (millions)**	0.012	0.137
**Age (years) M**		
Mean (SD)	69.1 (10.6)	69.2 (10.4)
Median (IQR)	70.0 (62.0, 77.0)	70.0 (63.0, 77.0)
**Age (years) I**		
18-59	935 (17.4)	4,408 (16.9)
60-79	3,566 (66.4)	17,524 (67.1)
>=80	868 (16.2)	4,184 (16.0)
**Sex M,I**		
male	2,916 (54.3)	14,217 (54.4)
female	2,453 (45.7)	11,899 (45.6)
**Index of multiple deprivation quintile (patient area) SRF,I**		
1 (least deprived)	956 (17.8)	5,550 (21.3)
2	1,044 (19.4)	5,766 (22.1)
3	1,076 (20.0)	5,526 (21.2)
4	1,107 (20.6)	4,809 (18.4)
5 (most deprived)	1,186 (22.1)	4,465 (17.1)
**Year of cancer diagnosis M,I**		
1989 to 1998	532 (9.9)	2,618 (10.0)
1999 to 2003	1,191 (22.2)	5,756 (22.0)
2004 to 2008	1,825 (34.0)	8,808 (33.7)
2009 to 2014	1,821 (33.9)	8,934 (34.2)
**Smoking status SRF,I**		
non-smoker	657 (12.2)	11,397 (43.6)
current smoker	2,158 (40.2)	4,399 (16.8)
ex-smoker	2,554 (47.6)	10,320 (39.5)
**Heavy drinker SRF**	149 (2.8)	1,197 (4.6)
**Body Mass Index SRF(spline),I(categorical)**		
underweight	251 (4.7)	424 (1.6)
healthy weight	2,266 (42.2)	9,098 (34.8)
overweight	1,894 (35.3)	10,618 (40.7)
obese	958 (17.8)	5,976 (22.9)
**Diabetes SRF**	655 (12.2)	3,224 (12.3)
**Hypertension SRF,I**	1,323 (24.6)	6,368 (24.4)
**Previous cardiovascular disease SRF,I**	2,191 (40.8)	8,600 (32.9)
**Cardiovascular treatments SRF**		
**Statins**	1,696 (31.6)	7,496 (28.7)
**Beta blockers**	901 (16.8)	4,853 (18.6)
**Angiotensin converting enzyme (ACE) inhibitors**	1,164 (21.7)	5,486 (21.0)
**Angiotensin II receptor blockers (ARBs)**	405 (7.5)	2,000 (7.7)
**Non steroidal anti-inflammatory drugs (NSAIDs)**	704 (13.1)	2,870 (11.0)
**Previous migraine SRF**	282 (5.3)	1,498 (5.7)
**Chronic kidney disease SRF**	910 (16.9)	4,015 (15.4)
**Previous systemic sclerosis**	9 (0.2)	33 (0.1)
**Previous COPD**	1,808 (33.7)	3,226 (12.4)

Data are n (%) and represent status at the matched diagnosis date (baseline) unless stated otherwise. * index date to end of follow-up. M Matching variable, SRF Shared Risk Factor, I Interactions investigated.

Table B8: Characteristics of MALIGNANT MELANOMA cancer (ICD10 C43) survivors and matched controls

	Cancer survivors	Controls
**N**	7,098	34,108
**Person-years from cancer the matched diagnosis date (baseline) to end of follow-up I**		
Mean (SD)	6.3 (4.3)	6.4 (4.3)
Median (IQR)	5.2 (3.0, 8.8)	5.3 (3.0, 8.9)
Range	1.0-26.3	1.0-26.3
**Total person-years included* (millions)**	0.038	0.185
**Age (years) M**		
Mean (SD)	60.5 (15.9)	60.6 (15.8)
Median (IQR)	62.0 (49.0, 72.0)	62.0 (49.0, 73.0)
**Age (years) I**		
18-59	3,223 (45.4)	15,354 (45.0)
60-79	3,011 (42.4)	14,629 (42.9)
>=80	864 (12.2)	4,125 (12.1)
**Sex M,I**		
male	3,084 (43.4)	14,486 (42.5)
female	4,014 (56.6)	19,622 (57.5)
**Index of multiple deprivation quintile (patient area) SRF,I**		
1 (least deprived)	2,136 (30.1)	9,560 (28.0)
2	1,817 (25.6)	8,175 (24.0)
3	1,523 (21.5)	7,300 (21.4)
4	1,025 (14.4)	5,441 (16.0)
5 (most deprived)	597 (8.4)	3,632 (10.6)
**Year of cancer diagnosis M,I**		
1989 to 1998	656 (9.2)	3,008 (8.8)
1999 to 2003	1,447 (20.4)	6,780 (19.9)
2004 to 2008	2,520 (35.5)	12,235 (35.9)
2009 to 2014	2,475 (34.9)	12,085 (35.4)
**Smoking status SRF,I**		
non-smoker	3,812 (53.7)	16,422 (48.1)
current smoker	992 (14.0)	6,338 (18.6)
ex-smoker	2,294 (32.3)	11,348 (33.3)
**Heavy drinker SRF**	294 (4.1)	1,533 (4.5)
**Body Mass Index SRF(spline) I(categorical)**		
underweight	91 (1.3)	630 (1.8)
healthy weight	2,754 (38.8)	13,484 (39.5)
overweight	2,784 (39.2)	12,604 (37.0)
obese	1,469 (20.7)	7,390 (21.7)
**Diabetes SRF**	532 (7.5)	2,881 (8.4)
**Hypertension SRF,I**	1,398 (19.7)	6,078 (17.8)
**Previous cardiovascular disease SRF,I**	1,715 (24.2)	8,014 (23.5)
**Cardiovascular treatments SRF**		
**Statins**	1,376 (19.4)	6,628 (19.4)
**Beta blockers**	905 (12.8)	4,342 (12.7)
**Angiotensin converting enzyme (ACE) inhibitors**	1,082 (15.2)	4,971 (14.6)
**Angiotensin II receptor blockers (ARBs)**	394 (5.6)	1,945 (5.7)
**Non steroidal anti-inflammatory drugs (NSAIDs)**	621 (8.7)	3,043 (8.9)
**Previous migraine SRF**	510 (7.2)	2,475 (7.3)
**Chronic kidney disease SRF**	783 (11.0)	3,594 (10.5)

Data are n (%) and represent status at the matched diagnosis date (baseline) unless stated otherwise. * index date to end of follow-up. M Matching variable, SRF Shared Risk Factor, I Interactions investigated.

Table B9: Characteristics of BREAST cancer (ICD10 C50) survivors and matched controls

	Cancer survivors	Controls
**N**	25,633	126,103
**Person-years from cancer the matched diagnosis date (baseline) to end of follow-up I**		
Mean (SD)	6.7 (4.4)	7.0 (4.5)
Median (IQR)	5.6 (3.1, 9.3)	6.0 (3.4, 9.7)
Range	1.0-26.3	1.0-26.6
**Total person-years included* (millions)**	0.145	0.757
**Age (years) M**		
Mean (SD)	61.6 (13.6)	61.6 (13.6)
Median (IQR)	61.0 (51.0, 71.0)	61.0 (51.0, 71.0)
**Age (years) I**		
18-59	11,647 (45.4)	57,594 (45.7)
60-79	11,055 (43.1)	54,260 (43.0)
>=80	2,931 (11.4)	14,249 (11.3)
**Sex M,I**		
female	25,633 (100.0)	126,103 (100.0)
**Index of multiple deprivation quintile (patient area) SRF,I**		
1 (least deprived)	6,794 (26.5)	32,820 (26.0)
2	6,206 (24.2)	29,913 (23.7)
3	5,397 (21.1)	26,637 (21.1)
4	4,139 (16.1)	20,727 (16.4)
5 (most deprived)	3,097 (12.1)	16,006 (12.7)
**Year of cancer diagnosis M,I**		
1989 to 1998	3,027 (11.8)	14,711 (11.7)
1999 to 2003	6,108 (23.8)	29,904 (23.7)
2004 to 2008	8,669 (33.8)	42,693 (33.9)
2009 to 2014	7,829 (30.5)	38,795 (30.8)
**Smoking status SRF,I**		
non-smoker	13,973 (54.5)	70,220 (55.7)
current smoker	4,205 (16.4)	22,032 (17.5)
ex-smoker	7,455 (29.1)	33,851 (26.8)
**Heavy drinker SRF**	807 (3.1)	4,111 (3.3)
**Body Mass Index SRF(spline) I(categorical)**		
underweight	447 (1.7)	2,665 (2.1)
healthy weight	10,615 (41.4)	52,775 (41.9)
overweight	8,491 (33.1)	41,410 (32.8)
obese	6,080 (23.7)	29,253 (23.2)
**Diabetes SRF**	1,853 (7.2)	8,668 (6.9)
**Hypertension SRF,I**	5,440 (21.2)	24,502 (19.4)
**Previous cardiovascular disease SRF,I**	5,710 (22.3)	27,197 (21.6)
**Cardiovascular treatments SRF**		
**Statins**	3,772 (14.7)	19,005 (15.1)
**Beta blockers**	3,120 (12.2)	15,607 (12.4)
**Angiotensin converting enzyme (ACE) inhibitors**	3,100 (12.1)	15,064 (11.9)
**Angiotensin II receptor blockers (ARBs)**	1,462 (5.7)	6,759 (5.4)
**Non steroidal anti-inflammatory drugs (NSAIDs)**	2,749 (10.7)	12,737 (10.1)
**Previous migraine SRF**	2,513 (9.8)	12,134 (9.6)
**Chronic kidney disease SRF**	2,796 (10.9)	13,808 (10.9)
**HRT prescription**		
never	17,678 (69.0)	90,010 (71.4)
HRT in year before cancer diagnosis	316 (1.2)	1,496 (1.2)
HRT stopped prior to baseline year	7,639 (29.8)	34,597 (27.4)
**Hysterectomy**	4,700 (18.3)	23,369 (18.5)

Data are n (%) and represent status at the matched diagnosis date (baseline) unless stated otherwise. * index date to end of follow-up. M Matching variable, SRF Shared Risk Factor, I Interactions investigated.

Table B10: Characteristics of CERVICAL cancer (ICD10 C53) survivors and matched controls

	Cancer survivors	Controls
**N**	1,209	6,119
**Person-years from cancer the matched diagnosis date (baseline) to end of follow-up I**		
Mean (SD)	6.3 (4.6)	7.3 (4.9)
Median (IQR)	5.0 (2.6, 8.7)	6.1 (3.4, 10.3)
Range	1.0-21.9	1.0-25.7
**Total person-years included* (millions)**	0.006	0.039
**Age (years) M**		
Mean (SD)	46.4 (16.2)	46.7 (16.1)
Median (IQR)	42.0 (34.0, 57.0)	43.0 (34.0, 57.0)
**Age (years) I**		
18-59	948 (78.4)	4,774 (78.0)
60-79	213 (17.6)	1,119 (18.3)
>=80	48 (4.0)	226 (3.7)
**Sex M,I**		
female	1,209 (100.0)	6,119 (100.0)
**Index of multiple deprivation quintile (patient area) SRF,I**		
1 (least deprived)	256 (21.2)	1,399 (22.9)
2	218 (18.0)	1,274 (20.8)
3	241 (19.9)	1,250 (20.4)
4	240 (19.9)	1,151 (18.8)
5 (most deprived)	254 (21.0)	1,045 (17.1)
**Year of cancer diagnosis M,I**		
1989 to 1998	176 (14.6)	876 (14.3)
1999 to 2003	269 (22.2)	1,427 (23.3)
2004 to 2008	391 (32.3)	1,936 (31.6)
2009 to 2014	373 (30.9)	1,880 (30.7)
**Smoking status SRF,I**		
non-smoker	522 (43.2)	3,293 (53.8)
current smoker	394 (32.6)	1,456 (23.8)
ex-smoker	293 (24.2)	1,370 (22.4)
**Heavy drinker SRF**	37 (3.1)	183 (3.0)
**Body Mass Index SRF(spline) I(categorical)**		
underweight	41 (3.4)	146 (2.4)
healthy weight	562 (46.5)	2,853 (46.6)
overweight	329 (27.2)	1,797 (29.4)
obese	277 (22.9)	1,323 (21.6)
**Diabetes SRF**	41 (3.4)	219 (3.6)
**Hypertension SRF,I**	156 (12.9)	667 (10.9)
**Previous cardiovascular disease SRF,I**	149 (12.3)	821 (13.4)
**Cardiovascular treatments SRF**		
**Statins**	67 (5.5)	425 (6.9)
**Beta blockers**	72 (6.0)	428 (7.0)
**Angiotensin converting enzyme (ACE) inhibitors**	58 (4.8)	359 (5.9)
**Angiotensin II receptor blockers (ARBs)**	27 (2.2)	142 (2.3)
**Non steroidal anti-inflammatory drugs (NSAIDs)**	106 (8.8)	419 (6.8)
**Previous migraine SRF**	143 (11.8)	709 (11.6)
**Chronic kidney disease SRF**	67 (5.5)	278 (4.5)
**HRT prescription**		
never	1,083 (89.6)	5,292 (86.5)
HRT in year before cancer diagnosis	14 (1.2)	40 (0.7)
HRT stopped prior to baseline year	112 (9.3)	787 (12.9)
**Hysterectomy**	34 (2.8)	563 (9.2)

Data are n (%) and represent status at the matched diagnosis date (baseline) unless stated otherwise. * index date to end of follow-up. M Matching variable, SRF Shared Risk Factor, I Interactions investigated.

Table B11: Characteristics of UTERUS cancer (ICD10 C54-55) survivors and matched controls

	Cancer survivors	Controls
**N**	3,440	16,913
**Person-years from cancer the matched diagnosis date (baseline) to end of follow-up I**		
Mean (SD)	6.3 (4.3)	6.6 (4.3)
Median (IQR)	5.3 (2.9, 8.8)	5.7 (3.3, 9.2)
Range	1.0-23.3	1.0-24.6
**Total person-years included* (millions)**	0.018	0.095
**Age (years) M**		
Mean (SD)	65.4 (10.9)	65.3 (10.8)
Median (IQR)	65.0 (58.0, 73.0)	65.0 (58.0, 73.0)
**Age (years) I**		
18-59	1,029 (29.9)	5,082 (30.0)
60-79	2,062 (59.9)	10,117 (59.8)
>=80	349 (10.1)	1,714 (10.1)
**Sex M,I**		
female	3,440 (100.0)	16,913 (100.0)
**Index of multiple deprivation quintile (patient area) SRF,I**		
1 (least deprived)	816 (23.7)	4,047 (23.9)
2	819 (23.8)	3,997 (23.6)
3	752 (21.9)	3,598 (21.3)
4	604 (17.6)	2,912 (17.2)
5 (most deprived)	449 (13.1)	2,359 (13.9)
**Year of cancer diagnosis M,I**		
1989 to 1998	330 (9.6)	1,502 (8.9)
1999 to 2003	745 (21.7)	3,658 (21.6)
2004 to 2008	1,238 (36.0)	6,135 (36.3)
2009 to 2014	1,127 (32.8)	5,618 (33.2)
**Smoking status SRF,I**		
non-smoker	2,053 (59.7)	9,173 (54.2)
current smoker	361 (10.5)	2,748 (16.2)
ex-smoker	1,026 (29.8)	4,992 (29.5)
**Heavy drinker SRF**	73 (2.1)	472 (2.8)
**Body Mass Index SRF(spline) I(categorical)**		
underweight	35 (1.0)	344 (2.0)
healthy weight	764 (22.2)	6,579 (38.9)
overweight	1,002 (29.1)	5,721 (33.8)
obese	1,639 (47.6)	4,269 (25.2)
**Diabetes SRF**	522 (15.2)	1,412 (8.3)
**Hypertension SRF,I**	1,022 (29.7)	3,778 (22.3)
**Previous cardiovascular disease SRF,I**	848 (24.7)	4,124 (24.4)
**Cardiovascular treatments SRF**		
**Statins**	783 (22.8)	3,369 (19.9)
**Beta blockers**	692 (20.1)	2,576 (15.2)
**Angiotensin converting enzyme (ACE) inhibitors**	686 (19.9)	2,487 (14.7)
**Angiotensin II receptor blockers (ARBs)**	327 (9.5)	1,182 (7.0)
**Non steroidal anti-inflammatory drugs (NSAIDs)**	413 (12.0)	1,847 (10.9)
**Previous migraine SRF**	288 (8.4)	1,603 (9.5)
**Chronic kidney disease SRF**	500 (14.5)	2,140 (12.7)
**HRT prescription**		
never	2,513 (73.1)	11,299 (66.8)
HRT in year before cancer diagnosis	67 (1.9)	150 (0.9)
HRT stopped prior to baseline year	860 (25.0)	5,464 (32.3)
**Hysterectomy**	163 (4.7)	3,704 (21.9)

Data are n (%) and represent status at the matched diagnosis date (baseline) unless stated otherwise. * index date to end of follow-up. M Matching variable, SRF Shared Risk Factor, I Interactions investigated.

Table B12: Characteristics of OVARIAN cancer (ICD10 C56) survivors and matched controls

	Cancer survivors	Controls
N	2,710	13,459
**Person-years from cancer the matched diagnosis date (baseline) to end of follow-up I**		
Mean (SD)	5.2 (4.2)	7.1 (4.6)
Median (IQR)	3.8 (2.1, 7.1)	6.1 (3.4, 9.9)
Range	1.0-23.9	1.0-26.6
**Total person-years included* (millions)**	0.011	0.082
**Age (years) M**		
Mean (SD)	60.5 (14.1)	60.3 (14.1)
Median (IQR)	62.0 (51.0, 71.0)	61.0 (51.0, 70.0)
**Age (years) I**		
18-59	1,195 (44.1)	5,978 (44.4)
60-79	1,295 (47.8)	6,440 (47.8)
>=80	220 (8.1)	1,041 (7.7)
**Sex M,I**		
female	2,710 (100.0)	13,459 (100.0)
**Index of multiple deprivation quintile (patient area) SRF,I**		
1 (least deprived)	676 (24.9)	3,363 (25.0)
2	638 (23.5)	3,122 (23.2)
3	543 (20.0)	2,851 (21.2)
4	462 (17.0)	2,286 (17.0)
5 (most deprived)	391 (14.4)	1,837 (13.6)
**Year of cancer diagnosis M,I**		
1989 to 1998	310 (11.4)	1,590 (11.8)
1999 to 2003	674 (24.9)	3,287 (24.4)
2004 to 2008	898 (33.1)	4,439 (33.0)
2009 to 2014	828 (30.6)	4,143 (30.8)
**Smoking status SRF,I**		
non-smoker	1,507 (55.6)	7,496 (55.7)
current smoker	476 (17.6)	2,349 (17.5)
ex-smoker	727 (26.8)	3,614 (26.9)
**Heavy drinker SRF**	59 (2.2)	442 (3.3)
**Body Mass Index SRF(spline) I(categorical)**		
underweight	46 (1.7)	290 (2.2)
healthy weight	1,088 (40.1)	5,703 (42.4)
overweight	894 (33.0)	4,390 (32.6)
obese	682 (25.2)	3,076 (22.9)
**Diabetes SRF**	182 (6.7)	890 (6.6)
**Hypertension SRF,I**	549 (20.3)	2,477 (18.4)
**Previous cardiovascular disease SRF,I**	622 (23.0)	2,719 (20.2)
**Cardiovascular treatments SRF**		
**Statins**	425 (15.7)	1,937 (14.4)
**Beta blockers**	327 (12.1)	1,601 (11.9)
**Angiotensin converting enzyme (ACE) inhibitors**	302 (11.1)	1,448 (10.8)
**Angiotensin II receptor blockers (ARBs)**	155 (5.7)	725 (5.4)
**Non steroidal anti-inflammatory drugs (NSAIDs)**	296 (10.9)	1,413 (10.5)
**Previous migraine SRF**	274 (10.1)	1,304 (9.7)
**Chronic kidney disease SRF**	310 (11.4)	1,356 (10.1)
**HRT prescription**		
never	1,922 (70.9)	9,617 (71.5)
HRT in year before cancer diagnosis	39 (1.4)	167 (1.2)
HRT stopped prior to baseline year	749 (27.6)	3,675 (27.3)
**Hysterectomy**	536 (19.8)	2,494 (18.5)

Data are n (%) and represent status at the matched diagnosis date (baseline) unless stated otherwise. * index date to end of follow-up. M Matching variable, SRF Shared Risk Factor, I Interactions investigated.

Table B13: Characteristics of PROSTATE cancer (ICD10 C61) survivors and matched controls

	Cancer survivors	Controls
**N**	20,709	98,690
**Person-years from cancer the matched diagnosis date (baseline) to end of follow-up I**		
Mean (SD)	5.6 (3.7)	5.8 (3.7)
Median (IQR)	4.7 (2.7, 7.6)	4.9 (2.9, 7.9)
Range	1.0-23.7	1.0-25.8
**Total person-years included* (millions)**	0.095	0.472
**Age (years) M**		
Mean (SD)	71.5 (9.0)	71.5 (8.8)
Median (IQR)	72.0 (65.0, 78.0)	72.0 (65.0, 78.0)
**Age (years) I**		
18-59	2,009 (9.7)	9,270 (9.4)
60-79	14,744 (71.2)	70,714 (71.7)
>=80	3,956 (19.1)	18,706 (19.0)
**Sex M,I**		
male	20,709 (100.0)	98,690 (100.0)
**Index of multiple deprivation quintile (patient area) SRF,I**		
1 (least deprived)	5,898 (28.5)	26,612 (27.0)
2	5,040 (24.3)	23,720 (24.0)
3	4,519 (21.8)	21,345 (21.6)
4	3,069 (14.8)	15,631 (15.8)
5 (most deprived)	2,183 (10.5)	11,382 (11.5)
**Year of cancer diagnosis M,I**		
1989 to 1998	1,745 (8.4)	7,932 (8.0)
1999 to 2003	4,620 (22.3)	21,701 (22.0)
2004 to 2008	7,225 (34.9)	34,690 (35.2)
2009 to 2014	7,119 (34.4)	34,367 (34.8)
**Smoking status SRF,I**		
non-smoker	8,168 (39.4)	36,205 (36.7)
current smoker	2,768 (13.4)	15,779 (16.0)
ex-smoker	9,773 (47.2)	46,706 (47.3)
**Heavy drinker SRF**	922 (4.5)	5,092 (5.2)
**Body Mass Index SRF(spline) I(categorical)**		
underweight	152 (0.7)	912 (0.9)
healthy weight	7,028 (33.9)	32,995 (33.4)
overweight	9,782 (47.2)	45,101 (45.7)
obese	3,747 (18.1)	19,682 (19.9)
**Diabetes SRF**	2,499 (12.1)	14,672 (14.9)
**Hypertension SRF,I**	5,833 (28.2)	24,731 (25.1)
**Previous cardiovascular disease SRF,I**	7,510 (36.3)	37,013 (37.5)
**Cardiovascular treatments SRF**		
**Statins**	6,600 (31.9)	33,226 (33.7)
**Beta blockers**	3,756 (18.1)	19,467 (19.7)
**Angiotensin converting enzyme (ACE) inhibitors**	4,849 (23.4)	24,327 (24.6)
**Angiotensin II receptor blockers (ARBs)**	1,654 (8.0)	7,673 (7.8)
**Non steroidal anti-inflammatory drugs (NSAIDs)**	2,574 (12.4)	10,143 (10.3)
**Previous migraine SRF**	819 (4.0)	3,275 (3.3)
**Chronic kidney disease SRF**	3,859 (18.6)	15,788 (16.0)

Data are n (%) and represent status at the matched diagnosis date (baseline) unless stated otherwise. * index date to end of follow-up. M Matching variable, SRF Shared Risk Factor, I Interactions investigated.

Table B14: Characteristics of KIDNEY cancer (ICD10 C64) survivors and matched controls

	Cancer survivors	Controls
**N**	2,197	10,441
**Person-years from cancer the matched diagnosis date (baseline) to end of follow-up I**		
Mean (SD)	5.2 (3.8)	6.2 (4.2)
Median (IQR)	4.1 (2.3, 7.0)	5.1 (3.0, 8.4)
Range	1.0-22.7	1.0-25.1
**Total person-years included* (millions)**	0.009	0.054
**Age (years) M**		
Mean (SD)	65.9 (12.5)	65.8 (12.6)
Median (IQR)	67.0 (58.0, 75.0)	67.0 (58.0, 75.0)
**Age (years) I**		
18-59	626 (28.5)	2,999 (28.7)
60-79	1,275 (58.0)	6,043 (57.9)
>=80	296 (13.5)	1,399 (13.4)
**Sex M,I**		
male	1,330 (60.5)	6,299 (60.3)
female	867 (39.5)	4,142 (39.7)
**Index of multiple deprivation quintile (patient area) SRF,I**		
1 (least deprived)	526 (23.9)	2,570 (24.6)
2	520 (23.7)	2,393 (22.9)
3	474 (21.6)	2,247 (21.5)
4	379 (17.3)	1,810 (17.3)
5 (most deprived)	298 (13.6)	1,421 (13.6)
**Year of cancer diagnosis M,I**		
1989 to 1998	209 (9.5)	983 (9.4)
1999 to 2003	411 (18.7)	1,960 (18.8)
2004 to 2008	768 (35.0)	3,629 (34.8)
2009 to 2014	809 (36.8)	3,869 (37.1)
**Smoking status SRF,I**		
non-smoker	857 (39.0)	4,619 (44.2)
current smoker	449 (20.4)	1,851 (17.7)
ex-smoker	891 (40.6)	3,971 (38.0)
**Heavy drinker SRF**	57 (2.6)	518 (5.0)
**Body Mass Index SRF(spline) I(categorical)**		
underweight	25 (1.1)	141 (1.4)
healthy weight	689 (31.4)	3,715 (35.6)
overweight	854 (38.9)	4,244 (40.6)
obese	629 (28.6)	2,341 (22.4)
**Diabetes SRF**	355 (16.2)	1,115 (10.7)
**Hypertension SRF,I**	639 (29.1)	2,196 (21.0)
**Previous cardiovascular disease SRF,I**	769 (35.0)	3,037 (29.1)
**Cardiovascular treatments SRF**		
**Statins**	670 (30.5)	2,681 (25.7)
**Beta blockers**	439 (20.0)	1,694 (16.2)
**Angiotensin converting enzyme (ACE) inhibitors**	583 (26.5)	1,958 (18.8)
**Angiotensin II receptor blockers (ARBs)**	250 (11.4)	705 (6.8)
**Non steroidal anti-inflammatory drugs (NSAIDs)**	275 (12.5)	1,025 (9.8)
**Previous migraine SRF**	139 (6.3)	607 (5.8)
**Chronic kidney disease SRF**	504 (22.9)	1,348 (12.9)

Data are n (%) and represent status at the matched diagnosis date (baseline) unless stated otherwise. * index date to end of follow-up. M Matching variable, SRF Shared Risk Factor, I Interactions investigated.

Table B15: Characteristics of BLADDER cancer (ICD10 C67) survivors and matched controls

	Cancer survivors	Controls
**N**	7,712	36,886
**Person-years from cancer the matched diagnosis date (baseline) to end of follow-up I**		
Mean (SD)	5.9 (4.1)	6.2 (4.1)
Median (IQR)	4.9 (2.7, 8.2)	5.3 (3.0, 8.5)
Range	1.0-26.5	1.0-26.5
**Total person-years included* (millions)**	0.038	0.193
**Age (years) M**		
Mean (SD)	71.1 (11.2)	71.0 (11.0)
Median (IQR)	72.0 (64.0, 79.0)	72.0 (64.0, 79.0)
**Age (years) I**		
18-59	1,122 (14.5)	5,280 (14.3)
60-79	4,840 (62.8)	23,422 (63.5)
>=80	1,750 (22.7)	8,184 (22.2)
**Sex M,I**		
male	5,857 (75.9)	27,840 (75.5)
female	1,855 (24.1)	9,046 (24.5)
**Index of multiple deprivation quintile (patient area) SRF,I**		
1 (least deprived)	1,766 (22.9)	8,839 (24.0)
2	1,826 (23.7)	8,823 (23.9)
3	1,617 (21.0)	7,847 (21.3)
4	1,395 (18.1)	6,300 (17.1)
5 (most deprived)	1,108 (14.4)	5,077 (13.8)
**Year of cancer diagnosis M,I**		
1989 to 1998	927 (12.0)	4,302 (11.7)
1999 to 2003	1,840 (23.9)	8,752 (23.7)
2004 to 2008	2,684 (34.8)	12,882 (34.9)
2009 to 2014	2,261 (29.3)	10,950 (29.7)
**Smoking status SRF,I**		
non-smoker	2,264 (29.4)	15,391 (41.7)
current smoker	1,924 (24.9)	6,026 (16.3)
ex-smoker	3,524 (45.7)	15,469 (41.9)
**Heavy drinker SRF**	307 (4.0)	1,727 (4.7)
**Body Mass Index SRF(spline) I(categorical)**		
underweight	133 (1.7)	452 (1.2)
healthy weight	2,638 (34.2)	13,125 (35.6)
overweight	3,301 (42.8)	15,729 (42.6)
obese	1,640 (21.3)	7,580 (20.5)
**Diabetes SRF**	1,152 (14.9)	4,773 (12.9)
**Hypertension SRF,I**	2,215 (28.7)	9,398 (25.5)
**Previous cardiovascular disease SRF,I**	3,133 (40.6)	13,376 (36.3)
**Cardiovascular treatments SRF**		
**Statins**	2,523 (32.7)	10,744 (29.1)
**Beta blockers**	1,559 (20.2)	7,105 (19.3)
**Angiotensin converting enzyme (ACE) inhibitors**	1,831 (23.7)	8,237 (22.3)
**Angiotensin II receptor blockers (ARBs)**	620 (8.0)	2,573 (7.0)
**Non steroidal anti-inflammatory drugs (NSAIDs)**	805 (10.4)	3,927 (10.6)
**Previous migraine SRF**	320 (4.1)	1,600 (4.3)
**Chronic kidney disease SRF**	1,795 (23.3)	6,555 (17.8)

Data are n (%) and represent status at the matched diagnosis date (baseline) unless stated otherwise. * index date to end of follow-up. M Matching variable, SRF Shared Risk Factor, I Interactions investigated.

Table B16: Characteristics of BRAIN/CENTRAL NERVOUS SYSTEM cancer (ICD10 C71-2) survivors and controls

	Cancer survivors	Controls
N	906	4,364
**Person-years from cancer the matched diagnosis date (baseline) to end of follow-up I**		
Mean (SD)	4.6 (3.9)	6.9 (4.6)
Median (IQR)	3.2 (1.6, 6.4)	5.8 (3.2, 9.5)
Range	1.0-24.3	1.0-24.4
**Total person-years included* (millions)**	0.003	0.026
**Age (years) M**		
Mean (SD)	52.8 (15.0)	53.0 (14.9)
Median (IQR)	54.0 (41.0, 64.0)	54.0 (42.0, 64.0)
**Age (years) I**		
18-59	579 (63.9)	2,784 (63.8)
60-79	298 (32.9)	1,446 (33.1)
>=80	29 (3.2)	134 (3.1)
**Sex M,I**		
male	488 (53.9)	2,273 (52.1)
female	418 (46.1)	2,091 (47.9)
**Index of multiple deprivation quintile (patient area) SRF,I**		
1 (least deprived)	244 (26.9)	1,112 (25.5)
2	199 (22.0)	963 (22.1)
3	209 (23.1)	901 (20.6)
4	128 (14.1)	782 (17.9)
5 (most deprived)	126 (13.9)	606 (13.9)
**Year of cancer diagnosis M,I**		
1989 to 1998	99 (10.9)	480 (11.0)
1999 to 2003	197 (21.7)	927 (21.2)
2004 to 2008	300 (33.1)	1,465 (33.6)
2009 to 2014	310 (34.2)	1,492 (34.2)
**Smoking status SRF,I**		
non-smoker	472 (52.1)	2,152 (49.3)
current smoker	177 (19.5)	1,037 (23.8)
ex-smoker	257 (28.4)	1,175 (26.9)
**Heavy drinker SRF**	17 (1.9)	231 (5.3)
**Body Mass Index SRF(spline) I(categorical)**		
underweight	13 (1.4)	69 (1.6)
healthy weight	356 (39.3)	1,728 (39.6)
overweight	343 (37.9)	1,661 (38.1)
obese	194 (21.4)	906 (20.8)
**Diabetes SRF**	63 (7.0)	296 (6.8)
**Hypertension SRF,I**	156 (17.2)	583 (13.4)
**Previous cardiovascular disease SRF,I**	194 (21.4)	713 (16.3)
**Cardiovascular treatments SRF**		
**Statins**	146 (16.1)	608 (13.9)
**Beta blockers**	98 (10.8)	360 (8.2)
**Angiotensin converting enzyme (ACE) inhibitors**	92 (10.2)	489 (11.2)
**Angiotensin II receptor blockers (ARBs)**	42 (4.6)	184 (4.2)
**Non steroidal anti-inflammatory drugs (NSAIDs)**	92 (10.2)	369 (8.5)
**Previous migraine SRF**	93 (10.3)	316 (7.2)
**Chronic kidney disease SRF**	45 (5.0)	212 (4.9)

Data are n (%) and represent status at the matched diagnosis date (baseline) unless stated otherwise. * index date to end of follow-up. M Matching variable, SRF Shared Risk Factor, I Interactions investigated.

Table B17: Characteristics of THYROID cancer (ICD10 C73) survivors and controls

	Cancer survivors	Controls
**N**	1,028	4,932
**Person-years from cancer the matched diagnosis date (baseline) to end of follow-up I**		
Mean (SD)	6.4 (4.2)	6.3 (4.2)
Median (IQR)	5.4 (3.0, 8.6)	5.3 (3.0, 8.6)
Range	1.0-22.5	1.0-25.5
**Total person-years included* (millions)**	0.006	0.026
**Age (years) M**		
Mean (SD)	52.7 (15.8)	52.6 (15.9)
Median (IQR)	52.0 (40.0, 65.0)	52.0 (40.0, 65.0)
**Age (years) I**		
18-59	678 (66.0)	3,253 (66.0)
60-79	298 (29.0)	1,426 (28.9)
>=80	52 (5.1)	253 (5.1)
**Sex M,I**		
male	218 (21.2)	1,049 (21.3)
female	810 (78.8)	3,883 (78.7)
**Index of multiple deprivation quintile (patient area) SRF,I**		
1 (least deprived)	279 (27.1)	1,208 (24.5)
2	231 (22.5)	1,085 (22.0)
3	201 (19.6)	1,019 (20.7)
4	179 (17.4)	912 (18.5)
5 (most deprived)	138 (13.4)	708 (14.4)
**Year of cancer diagnosis M,I**		
1989 to 1998	82 (8.0)	409 (8.3)
1999 to 2003	182 (17.7)	856 (17.4)
2004 to 2008	338 (32.9)	1,605 (32.5)
2009 to 2014	426 (41.4)	2,062 (41.8)
**Smoking status SRF,I**		
non-smoker	570 (55.4)	2,517 (51.0)
current smoker	159 (15.5)	1,036 (21.0)
ex-smoker	299 (29.1)	1,379 (28.0)
**Heavy drinker SRF**	35 (3.4)	187 (3.8)
**Body Mass Index SRF(spline) I(categorical)**		
underweight	12 (1.2)	103 (2.1)
healthy weight	408 (39.7)	2,066 (41.9)
overweight	349 (33.9)	1,653 (33.5)
obese	259 (25.2)	1,110 (22.5)
**Diabetes SRF**	70 (6.8)	297 (6.0)
**Hypertension SRF,I**	144 (14.0)	639 (13.0)
**Previous cardiovascular disease SRF,I**	221 (21.5)	827 (16.8)
**Cardiovascular treatments SRF**		
**Statins**	141 (13.7)	632 (12.8)
**Beta blockers**	117 (11.4)	435 (8.8)
**Angiotensin converting enzyme (ACE) inhibitors**	98 (9.5)	482 (9.8)
**Angiotensin II receptor blockers (ARBs)**	56 (5.4)	189 (3.8)
**Non steroidal anti-inflammatory drugs (NSAIDs)**	91 (8.9)	387 (7.8)
**Previous migraine SRF**	114 (11.1)	509 (10.3)
**Chronic kidney disease SRF**	75 (7.3)	275 (5.6)

Data are n (%) and represent status at the matched diagnosis date (baseline) unless stated otherwise. * index date to end of follow-up. M Matching variable, SRF Shared Risk Factor, I Interactions investigated.

Table B18: Characteristics of NON-HODGKIN LYMPHOMA (ICD10 C82-85) survivors and matched controls

	Cancer survivors	Controls
**N**	4,423	21,195
**Person-years from cancer the matched diagnosis date (baseline) to end of follow-up I**		
Mean (SD)	5.7 (4.0)	6.5 (4.2)
Median (IQR)	4.7 (2.7, 7.8)	5.5 (3.2, 8.9)
Range	1.0-25.1	1.0-25.8
**Total person-years included* (millions)**	0.021	0.117
**Age (years) M**		
Mean (SD)	64.3 (14.1)	64.5 (13.9)
Median (IQR)	66.0 (56.0, 75.0)	66.0 (56.0, 75.0)
**Age (years) I**		
18-59	1,499 (33.9)	7,001 (33.0)
60-79	2,328 (52.6)	11,332 (53.5)
>=80	596 (13.5)	2,862 (13.5)
**Sex M,I**		
male	2,259 (51.1)	10,700 (50.5)
female	2,164 (48.9)	10,495 (49.5)
**Index of multiple deprivation quintile (patient area) SRF,I**		
1 (least deprived)	1,161 (26.2)	5,522 (26.1)
2	1,069 (24.2)	5,016 (23.7)
3	931 (21.0)	4,584 (21.6)
4	729 (16.5)	3,427 (16.2)
5 (most deprived)	533 (12.1)	2,646 (12.5)
**Year of cancer diagnosis M,I**		
1989 to 1998	494 (11.2)	2,258 (10.7)
1999 to 2003	973 (22.0)	4,644 (21.9)
2004 to 2008	1,521 (34.4)	7,381 (34.8)
2009 to 2014	1,435 (32.4)	6,912 (32.6)
**Smoking status SRF,I**		
non-smoker	2,033 (46.0)	9,964 (47.0)
current smoker	839 (19.0)	3,809 (18.0)
ex-smoker	1,551 (35.1)	7,422 (35.0)
**Heavy drinker SRF**	136 (3.1)	1,005 (4.7)
**Body Mass Index SRF(spline) I(categorical)**		
underweight	77 (1.7)	384 (1.8)
healthy weight	1,760 (39.8)	7,850 (37.0)
overweight	1,685 (38.1)	8,265 (39.0)
obese	901 (20.4)	4,696 (22.2)
**Diabetes SRF**	474 (10.7)	2,096 (9.9)
**Hypertension SRF,I**	956 (21.6)	4,651 (21.9)
**Previous cardiovascular disease SRF,I**	1,253 (28.3)	5,698 (26.9)
**Cardiovascular treatments SRF**		
**Statins**	973 (22.0)	4,833 (22.8)
**Beta blockers**	668 (15.1)	3,272 (15.4)
**Angiotensin converting enzyme (ACE) inhibitors**	745 (16.8)	3,629 (17.1)
**Angiotensin II receptor blockers (ARBs)**	270 (6.1)	1,334 (6.3)
**Non steroidal anti-inflammatory drugs (NSAIDs)**	546 (12.3)	1,997 (9.4)
**Previous migraine SRF**	291 (6.6)	1,338 (6.3)
**Chronic kidney disease SRF**	736 (16.6)	2,687 (12.7)
**Previous immunosuppression**	189 (4.3)	303 (1.4)

Data are n (%) and represent status at the matched diagnosis date (baseline) unless stated otherwise. * index date to end of follow-up. M Matching variable, SRF Shared Risk Factor, I Interactions investigated.

Table B19: Characteristics of MULTIPLE MYELOMA (ICD10 C90) survivors and matched controls

	Cancer survivors	Controls
**N**	1,843	8,784
**Person-years from cancer the matched diagnosis date (baseline) to end of follow-up I**		
Mean (SD)	4.6 (3.1)	6.2 (4.1)
Median (IQR)	3.7 (2.2, 6.2)	5.2 (3.0, 8.5)
Range	1.0-22.0	1.0-25.0
**Total person-years included* (millions)**	0.007	0.046
**Age (years) M**		
Mean (SD)	69.3 (11.4)	69.2 (11.2)
Median (IQR)	70.0 (62.0, 78.0)	70.0 (62.0, 78.0)
**Age (years) I**		
18-59	361 (19.6)	1,716 (19.5)
60-79	1,130 (61.3)	5,432 (61.8)
>=80	352 (19.1)	1,636 (18.6)
**Sex M,I**		
male	970 (52.6)	4,576 (52.1)
female	873 (47.4)	4,208 (47.9)
**Index of multiple deprivation quintile (patient area) SRF,I**		
1 (least deprived)	439 (23.8)	2,117 (24.1)
2	429 (23.3)	2,062 (23.5)
3	381 (20.7)	1,882 (21.4)
4	336 (18.2)	1,456 (16.6)
5 (most deprived)	258 (14.0)	1,267 (14.4)
**Year of cancer diagnosis M,I**		
1989 to 1998	200 (10.9)	915 (10.4)
1999 to 2003	412 (22.4)	1,893 (21.6)
2004 to 2008	615 (33.4)	2,962 (33.7)
2009 to 2014	616 (33.4)	3,014 (34.3)
**Smoking status SRF,I**		
non-smoker	874 (47.4)	4,098 (46.7)
current smoker	271 (14.7)	1,388 (15.8)
ex-smoker	698 (37.9)	3,298 (37.5)
**Heavy drinker SRF**	33 (1.8)	355 (4.0)
**Body Mass Index SRF(spline) I(categorical)**		
underweight	37 (2.0)	147 (1.7)
healthy weight	687 (37.3)	3,104 (35.3)
overweight	725 (39.3)	3,586 (40.8)
obese	394 (21.4)	1,947 (22.2)
**Diabetes SRF**	209 (11.3)	1,041 (11.9)
**Hypertension SRF,I**	538 (29.2)	2,120 (24.1)
**Previous cardiovascular disease SRF,I**	620 (33.6)	2,711 (30.9)
**Cardiovascular treatments SRF**		
**Statins**	434 (23.5)	2,325 (26.5)
**Beta blockers**	347 (18.8)	1,565 (17.8)
**Angiotensin converting enzyme (ACE) inhibitors**	374 (20.3)	1,692 (19.3)
**Angiotensin II receptor blockers (ARBs)**	164 (8.9)	659 (7.5)
**Non steroidal anti-inflammatory drugs (NSAIDs)**	443 (24.0)	868 (9.9)
**Previous migraine SRF**	101 (5.5)	537 (6.1)
**Chronic kidney disease SRF**	551 (29.9)	1,348 (15.3)

Data are n (%) and represent status at the matched diagnosis date (baseline) unless stated otherwise. * index date to end of follow-up. M Matching variable, SRF Shared Risk Factor, I Interactions investigated.

Table B20: Characteristics of LEUKAEMIA (ICD10 C91-95) survivors and matched controls

	Cancer survivors	Controls
**N**	3,419	16,446
**Person-years from cancer the matched diagnosis date (baseline) to end of follow-up I**		
Mean (SD)	5.4 (3.8)	6.3 (4.2)
Median (IQR)	4.4 (2.4, 7.5)	5.3 (3.0, 8.8)
Range	1.0-24.9	1.0-26.3
**Total person-years included* (millions)**	0.015	0.088
**Age (years) M**		
Mean (SD)	67.2 (13.5)	67.3 (13.3)
Median (IQR)	69.0 (59.0, 77.0)	69.0 (59.0, 77.0)
**Age (years) I**		
18-59	873 (25.5)	4,132 (25.1)
60-79	1,918 (56.1)	9,314 (56.6)
>=80	628 (18.4)	3,000 (18.2)
**Sex M,I**		
male	2,014 (58.9)	9,591 (58.3)
female	1,405 (41.1)	6,855 (41.7)
**Index of multiple deprivation quintile (patient area) SRF,I**		
1 (least deprived)	892 (26.1)	4,116 (25.0)
2	827 (24.2)	3,943 (24.0)
3	679 (19.9)	3,563 (21.7)
4	572 (16.7)	2,719 (16.5)
5 (most deprived)	449 (13.1)	2,105 (12.8)
**Year of cancer diagnosis M,I**		
1989 to 1998	365 (10.7)	1,783 (10.8)
1999 to 2003	780 (22.8)	3,656 (22.2)
2004 to 2008	1,194 (34.9)	5,726 (34.8)
2009 to 2014	1,080 (31.6)	5,281 (32.1)
**Smoking status SRF,I**		
non-smoker	1,541 (45.1)	7,581 (46.1)
current smoker	542 (15.9)	2,806 (17.1)
ex-smoker	1,336 (39.1)	6,059 (36.8)
**Heavy drinker SRF**	109 (3.2)	700 (4.3)
**Body Mass Index SRF(spline) I(categorical)**		
underweight	51 (1.5)	262 (1.6)
healthy weight	1,265 (37.0)	6,034 (36.7)
overweight	1,366 (40.0)	6,613 (40.2)
obese	737 (21.6)	3,537 (21.5)
**Diabetes SRF**	386 (11.3)	1,853 (11.3)
**Hypertension SRF,I**	881 (25.8)	3,897 (23.7)
**Previous cardiovascular disease SRF,I**	1,145 (33.5)	5,079 (30.9)
**Cardiovascular treatments SRF**		
**Statins**	841 (24.6)	4,085 (24.8)
**Beta blockers**	589 (17.2)	2,793 (17.0)
**Angiotensin converting enzyme (ACE) inhibitors**	664 (19.4)	3,109 (18.9)
**Angiotensin II receptor blockers (ARBs)**	253 (7.4)	1,097 (6.7)
**Non steroidal anti-inflammatory drugs (NSAIDs)**	422 (12.3)	1,621 (9.9)
**Previous migraine SRF**	238 (7.0)	888 (5.4)
**Chronic kidney disease SRF**	672 (19.7)	2,498 (15.2)

Data are n (%) and represent status at the matched diagnosis date (baseline) unless stated otherwise. * index date to end of follow-up. M Matching variable, SRF Shared Risk Factor, I Interactions investigated.

Figure B2: Absolute and relative risk of cardiovascular disease in cancer survivors compared to general population controls

A to D): Coronary artery disease component outcomes

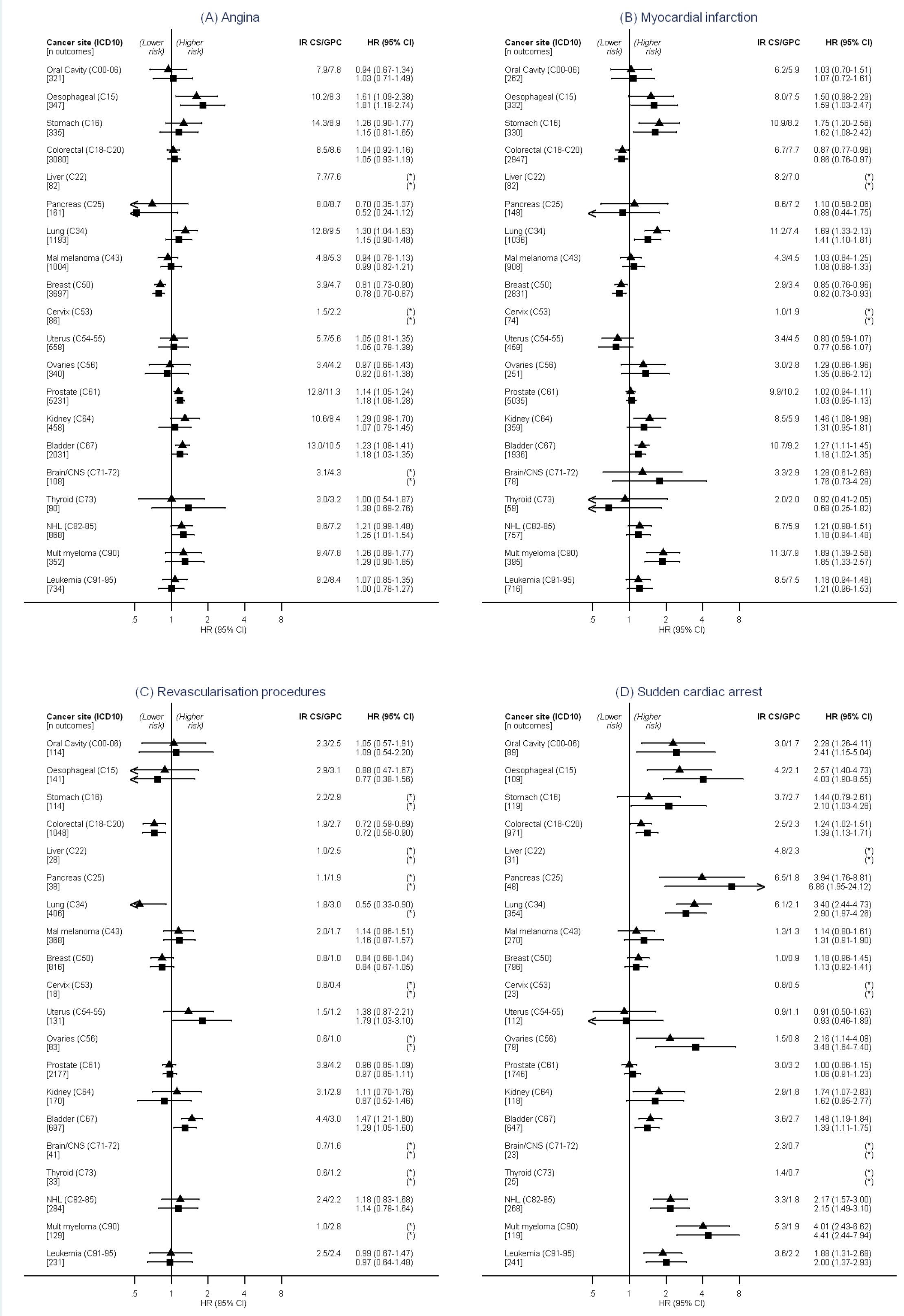

▲ Stratified by age and gender matched sets ■ Additionally adjusted for shared risk factors

(*) too few events for estimation; </> = CI limit <0.5 or >12
HR = hazard ratio, CI = confidence interval, IR = incidence rate per 1000 patient years, GPC = general population controls, CS = cancer survivors

E to H) Venous thromboembolism component outcomes and heart failure/cardiomyopathy

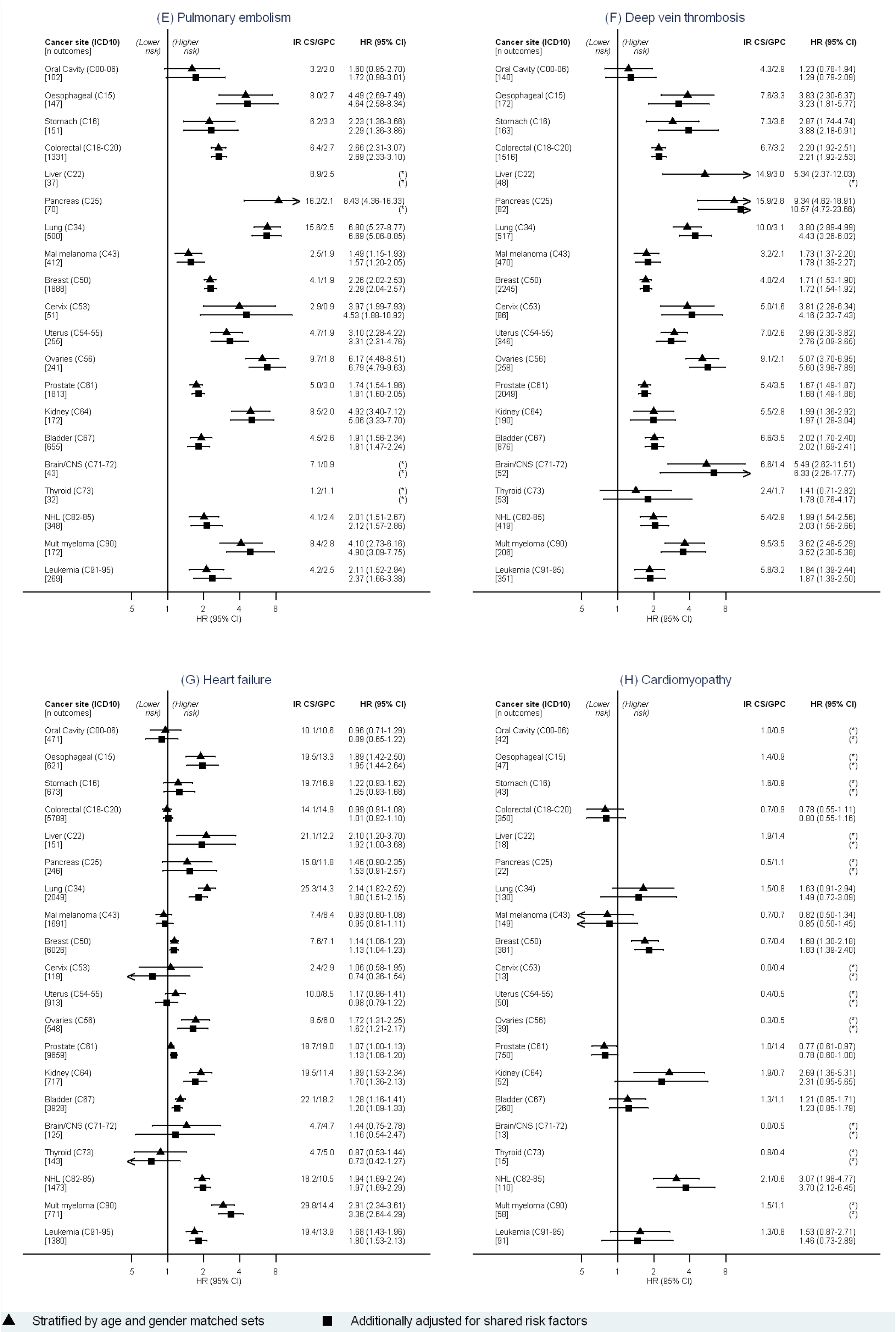

▲ Stratified by age and gender matched sets ■ Additionally adjusted for shared risk factors

(*) too few events for estimation; </> = CI limit <0.5 or >12
HR = hazard ratio, CI = confidence interval, IR = incidence rate per 1000 patient years, GPC = general population controls, CS = cancer survivors

Figure B3: Associations between site-specific cancer survivorship and specific CVD outcomes, stratified by other factors

A) CORONARY ARTERY DISEASE

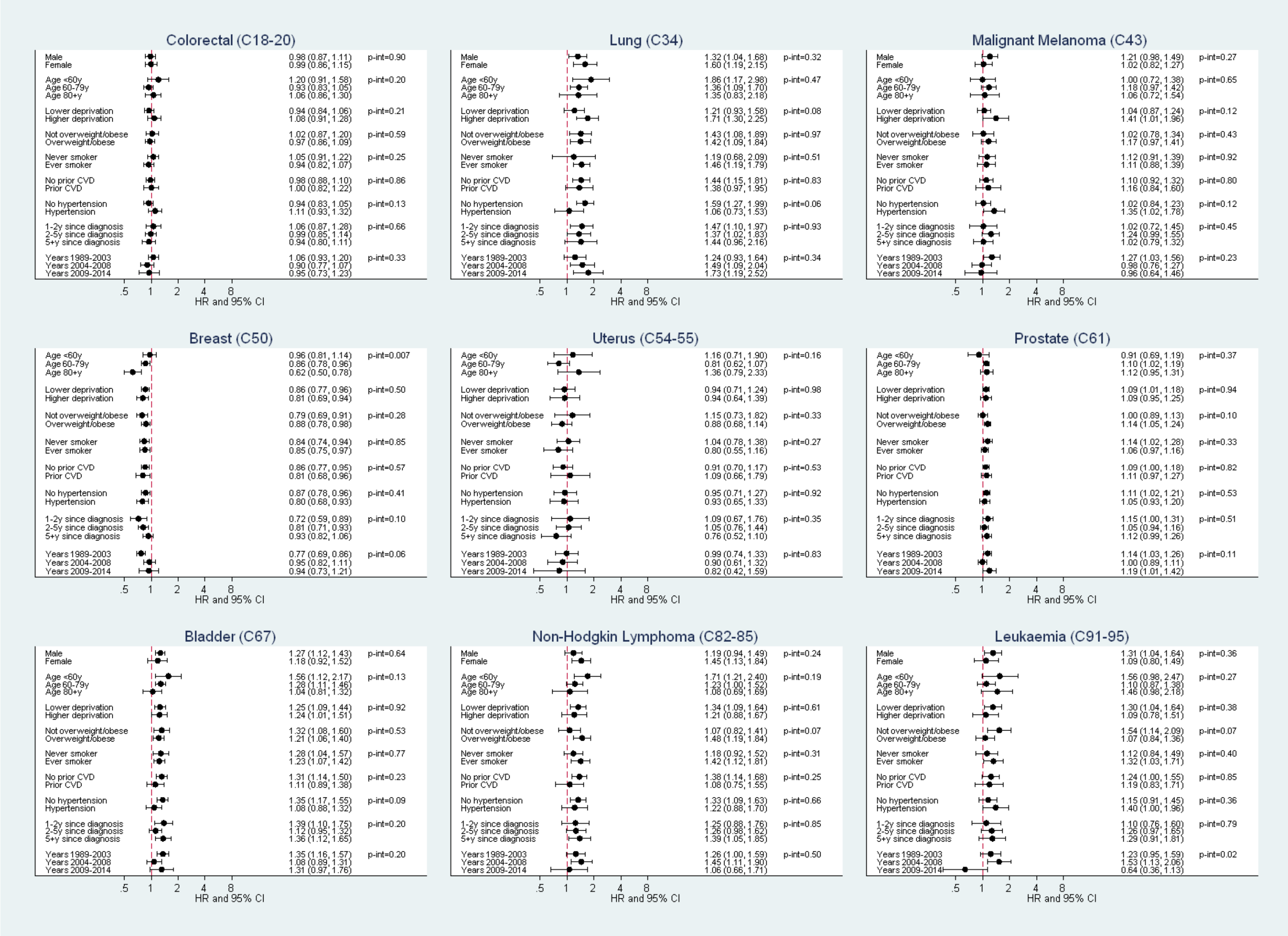

B) STROKE

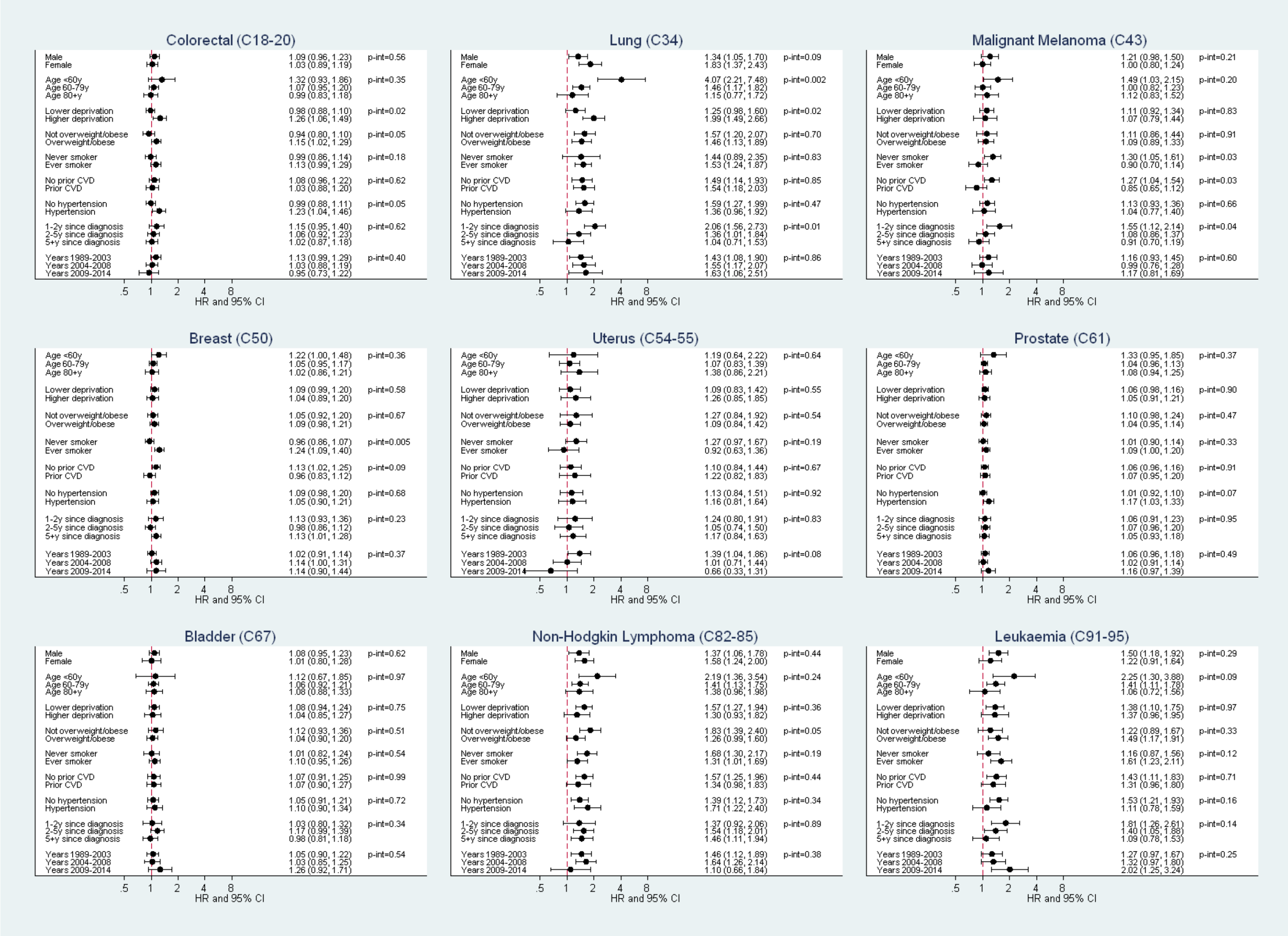

Colorectal (C18-20)
Male 1.09 (0.96, 1.23) p-int=0.56
Female 1.03 (0.89, 1.19)
Age <60y 1.32 (0.93, 1.86) p-int=0.35
Age 60-79y 1.07 (0.95, 1.20)
Age 80+y 0.99 (0.83, 1.18)
Lower deprivation 0.98 (0.88, 1.10) p-int=0.02
Higher deprivation 1.26 (1.06, 1.49)
Not overweight/obese 0.94 (0.80, 1.10) p-int=0.05
Overweight/obese 1.15 (1.02, 1.29)
Never smoker 0.99 (0.86, 1.14) p-int=0.18
Ever smoker 1.13 (0.99, 1.29)
No prior CVD 1.08 (0.96, 1.22) p-int=0.62
Prior CVD 1.03 (0.88, 1.20)
No hypertension 0.99 (0.88, 1.11) p-int=0.05
Hypertension 1.23 (1.04, 1.46)
1-2y since diagnosis 1.15 (0.95, 1.40) p-int=0.62
2-5y since diagnosis 1.06 (0.92, 1.23)
5+y since diagnosis 1.02 (0.87, 1.18)
Years 1989-2003 1.13 (0.99, 1.29) p-int=0.40
Years 2004-2008 1.03 (0.88, 1.19)
Years 2009-2014 0.95 (0.73, 1.22)
.5 1 2 4 8
HR and 95% CI
Lung (C34)
Male 1.34 (1.05, 1.70) p-int=0.09
Female 1.83 (1.37, 2.43)
Age <60y 4.07 (2.21, 7.48) p-int=0.002
Age 60-79y 1.46 (1.17, 1.82)
Age 80+y 1.15 (0.77, 1.72)
Lower deprivation 1.25 (0.98, 1.60) p-int=0.02
Higher deprivation 1.99 (1.49, 2.66)
Not overweight/obese 1.57 (1.20, 2.07) p-int=0.70
Overweight/obese 1.46 (1.13, 1.89)
Never smoker 1.44 (0.89, 2.35) p-int=0.83
Ever smoker 1.53 (1.24, 1.87)
No prior CVD 1.49 (1.14, 1.93) p-int=0.85
Prior CVD 1.54 (1.18, 2.03)
No hypertension 1.59 (1.27, 1.99) p-int=0.47
Hypertension 1.36 (0.96, 1.92)
1-2y since diagnosis 2.06 (1.56, 2.73) p-int=0.01
2-5y since diagnosis 1.36 (1.01, 1.84)
5+y since diagnosis 1.04 (0.71, 1.53)
Years 1989-2003 1.43 (1.08, 1.90) p-int=0.86
Years 2004-2008 1.55 (1.17, 2.07)
Years 2009-2014 1.63 (1.06, 2.51)
.5 1 2 4 8
HR and 95% CI
Malignant Melanoma (C43)
Male 1.21 (0.98, 1.50) p-int=0.21
Female 1.00 (0.80, 1.24)
Age <60y 1.49 (1.03, 2.15) p-int=0.20
Age 60-79y 1.00 (0.82, 1.23)
Age 80+y 1.12 (0.83, 1.52)
Lower deprivation 1.11 (0.92, 1.34) p-int=0.83
Higher deprivation 1.07 (0.79, 1.44)
Not overweight/obese 1.11 (0.86, 1.44) p-int=0.91
Overweight/obese 1.09 (0.89, 1.33)
Never smoker 1.30 (1.05, 1.61) p-int=0.03
Ever smoker 0.90 (0.70, 1.14)
No prior CVD 1.27 (1.04, 1.54) p-int=0.03
Prior CVD 0.85 (0.65, 1.12)
No hypertension 1.13 (0.93, 1.36) p-int=0.66
Hypertension 1.04 (0.77, 1.40)
1-2y since diagnosis 1.55 (1.12, 2.14) p-int=0.04
2-5y since diagnosis 1.08 (0.86, 1.37)
5+y since diagnosis 0.91 (0.70, 1.19)
Years 1989-2003 1.16 (0.93, 1.45) p-int=0.60
Years 2004-2008 0.99 (0.76, 1.28)
Years 2009-2014 1.17 (0.81, 1.69)
.5 1 2 4 8
HR and 95% CI
Breast (C50)
Age <60y 1.22 (1.00, 1.48) p-int=0.36
Age 60-79y 1.05 (0.95, 1.17)
Age 80+y 1.02 (0.86, 1.21)
Lower deprivation 1.09 (0.99, 1.20) p-int=0.58
Higher deprivation 1.04 (0.89, 1.20)
Not overweight/obese 1.05 (0.92, 1.20) p-int=0.67
Overweight/obese 1.09 (0.98, 1.21)
Never smoker 0.96 (0.86, 1.07) p-int=0.005
Ever smoker 1.24 (1.09, 1.40)
No prior CVD 1.13 (1.02, 1.25) p-int=0.09
Prior CVD 0.96 (0.83, 1.12)
No hypertension 1.09 (0.98, 1.20) p-int=0.68
Hypertension 1.05 (0.90, 1.21)
1-2y since diagnosis 1.13 (0.93, 1.36) p-int=0.23
2-5y since diagnosis 0.98 (0.86, 1.12)
5+y since diagnosis 1.13 (1.01, 1.28)
Years 1989-2003 1.02 (0.91, 1.14) p-int=0.37
Years 2004-2008 1.14 (1.00, 1.31)
Years 2009-2014 1.14 (0.90, 1.44)
.5 1 2 4 8
HR and 95% CI
Uterus (C54-55)
Age <60y 1.19 (0.64, 2.22) p-int=0.64
Age 60-79y 1.07 (0.83, 1.39)
Age 80+y 1.38 (0.86, 2.21)
Lower deprivation 1.09 (0.83, 1.42) p-int=0.55
Higher deprivation 1.26 (0.85, 1.85)
Not overweight/obese 1.27 (0.84, 1.92) p-int=0.54
Overweight/obese 1.09 (0.84, 1.42)
Never smoker 1.27 (0.97, 1.67) p-int=0.19
Ever smoker 0.92 (0.63, 1.36)
No prior CVD 1.10 (0.84, 1.44) p-int=0.67
Prior CVD 1.22 (0.82, 1.83)
No hypertension 1.13 (0.84, 1.51) p-int=0.92
Hypertension 1.16 (0.81, 1.64)
1-2y since diagnosis 1.24 (0.80, 1.91) p-int=0.83
2-5y since diagnosis 1.05 (0.74, 1.50)
5+y since diagnosis 1.17 (0.84, 1.63)
Years 1989-2003 1.39 (1.04, 1.86) p-int=0.08
Years 2004-2008 1.01 (0.71, 1.44)
Years 2009-2014 0.66 (0.33, 1.31)
.5 1 2 4 8
HR and 95% CI
Prostate (C61)
Age <60y 1.33 (0.95, 1.85) p-int=0.37
Age 60-79y 1.04 (0.96, 1.13)
Age 80+y 1.08 (0.94, 1.25)
Lower deprivation 1.06 (0.98, 1.16) p-int=0.90
Higher deprivation 1.05 (0.91, 1.21)
Not overweight/obese 1.10 (0.98, 1.24) p-int=0.47
Overweight/obese 1.04 (0.95, 1.14)
Never smoker 1.01 (0.90, 1.14) p-int=0.33
Ever smoker 1.09 (1.00, 1.20)
No prior CVD 1.06 (0.96, 1.16) p-int=0.91
Prior CVD 1.07 (0.95, 1.20)
No hypertension 1.01 (0.92, 1.10) p-int=0.07
Hypertension 1.17 (1.03, 1.33)
1-2y since diagnosis 1.06 (0.91, 1.23) p-int=0.95
2-5y since diagnosis 1.07 (0.96, 1.20)
5+y since diagnosis 1.05 (0.93, 1.18)
Years 1989-2003 1.06 (0.96, 1.18) p-int=0.49
Years 2004-2008 1.02 (0.91, 1.14)
Years 2009-2014 1.16 (0.97, 1.39)
.5 1 2 4 8
HR and 95% CI
Bladder (C67)
Male 1.08 (0.95, 1.23) p-int=0.62
Female 1.01 (0.80, 1.28)
Age <60y 1.12 (0.67, 1.85) p-int=0.97
Age 60-79y 1.06 (0.92, 1.21)
Age 80+y 1.08 (0.88, 1.33)
Lower deprivation 1.08 (0.94, 1.24) p-int=0.75
Higher deprivation 1.04 (0.85, 1.27)
Not overweight/obese 1.12 (0.93, 1.36) p-int=0.51
Overweight/obese 1.04 (0.90, 1.20)
Never smoker 1.01 (0.82, 1.24) p-int=0.54
Ever smoker 1.10 (0.95, 1.26)
No prior CVD 1.07 (0.91, 1.25) p-int=0.99
Prior CVD 1.07 (0.90, 1.27)
No hypertension 1.05 (0.91, 1.21) p-int=0.72
Hypertension 1.10 (0.90, 1.34)
1-2y since diagnosis 1.03 (0.80, 1.32) p-int=0.34
2-5y since diagnosis 1.17 (0.99, 1.39)
5+y since diagnosis 0.98 (0.81, 1.18)
Years 1989-2003 1.05 (0.90, 1.22) p-int=0.54
Years 2004-2008 1.03 (0.85, 1.25)
Years 2009-2014 1.26 (0.92, 1.71)
.5 1 2 4 8
HR and 95% CI
Non-Hodgkin Lymphoma (C82-85)
Male 1.37 (1.06, 1.78) p-int=0.44
Female 1.58 (1.24, 2.00)
Age <60y 2.19 (1.36, 3.54) p-int=0.24
Age 60-79y 1.41 (1.13, 1.75)
Age 80+y 1.38 (0.96, 1.98)
Lower deprivation 1.57 (1.27, 1.94) p-int=0.36
Higher deprivation 1.30 (0.93, 1.82)
Not overweight/obese 1.83 (1.39, 2.40) p-int=0.05
Overweight/obese 1.26 (0.99, 1.60)
Never smoker 1.68 (1.30, 2.17) p-int=0.19
Ever smoker 1.31 (1.01, 1.69)
No prior CVD 1.57 (1.25, 1.96) p-int=0.44
Prior CVD 1.34 (0.98, 1.83)
No hypertension 1.39 (1.12, 1.73) p-int=0.34
Hypertension 1.71 (1.22, 2.40)
1-2y since diagnosis 1.37 (0.92, 2.06) p-int=0.89
2-5y since diagnosis 1.54 (1.18, 2.01)
5+y since diagnosis 1.46 (1.11, 1.94)
Years 1989-2003 1.46 (1.12, 1.89) p-int=0.38
Years 2004-2008 1.64 (1.26, 2.14)
Years 2009-2014 1.10 (0.66, 1.84)
.5 1 2 4 8
HR and 95% CI
Leukaemia (C91-95)
Male 1.50 (1.18, 1.92) p-int=0.29
Female 1.22 (0.91, 1.64)
Age <60y 2.25 (1.30, 3.88) p-int=0.09
Age 60-79y 1.41 (1.11, 1.78)
Age 80+y 1.06 (0.72, 1.56)
Lower deprivation 1.38 (1.10, 1.75) p-int=0.97
Higher deprivation 1.37 (0.96, 1.95)
Not overweight/obese 1.22 (0.89, 1.67) p-int=0.33
Overweight/obese 1.49 (1.17, 1.91)
Never smoker 1.16 (0.87, 1.56) p-int=0.12
Ever smoker 1.61 (1.23, 2.11)
No prior CVD 1.43 (1.11, 1.83) p-int=0.71
Prior CVD 1.31 (0.96, 1.80)
No hypertension 1.53 (1.21, 1.93) p-int=0.16
Hypertension 1.11 (0.78, 1.59)
1-2y since diagnosis 1.81 (1.26, 2.61) p-int=0.14
2-5y since diagnosis 1.40 (1.05, 1.88)
5+y since diagnosis 1.09 (0.78, 1.53)
Years 1989-2003 1.27 (0.97, 1.67) p-int=0.25
Years 2004-2008 1.32 (0.97, 1.80)
Years 2009-2014 2.02 (1.25, 3.24)
.5 1 2 4 8
HR and 95% CI

C) HEART FAILURE/CARDIOMYOPATHY

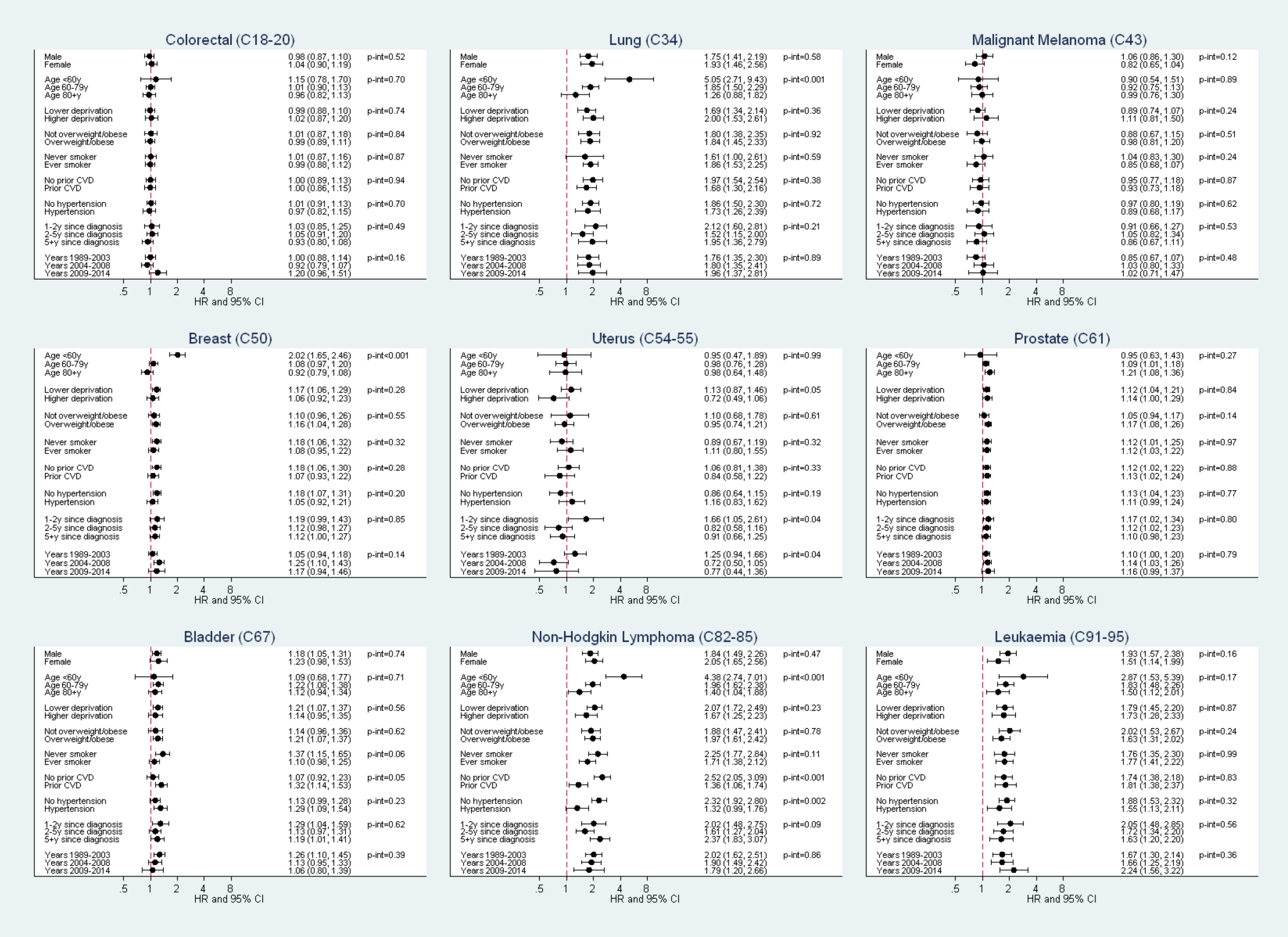

D) ARRHYTHMIA

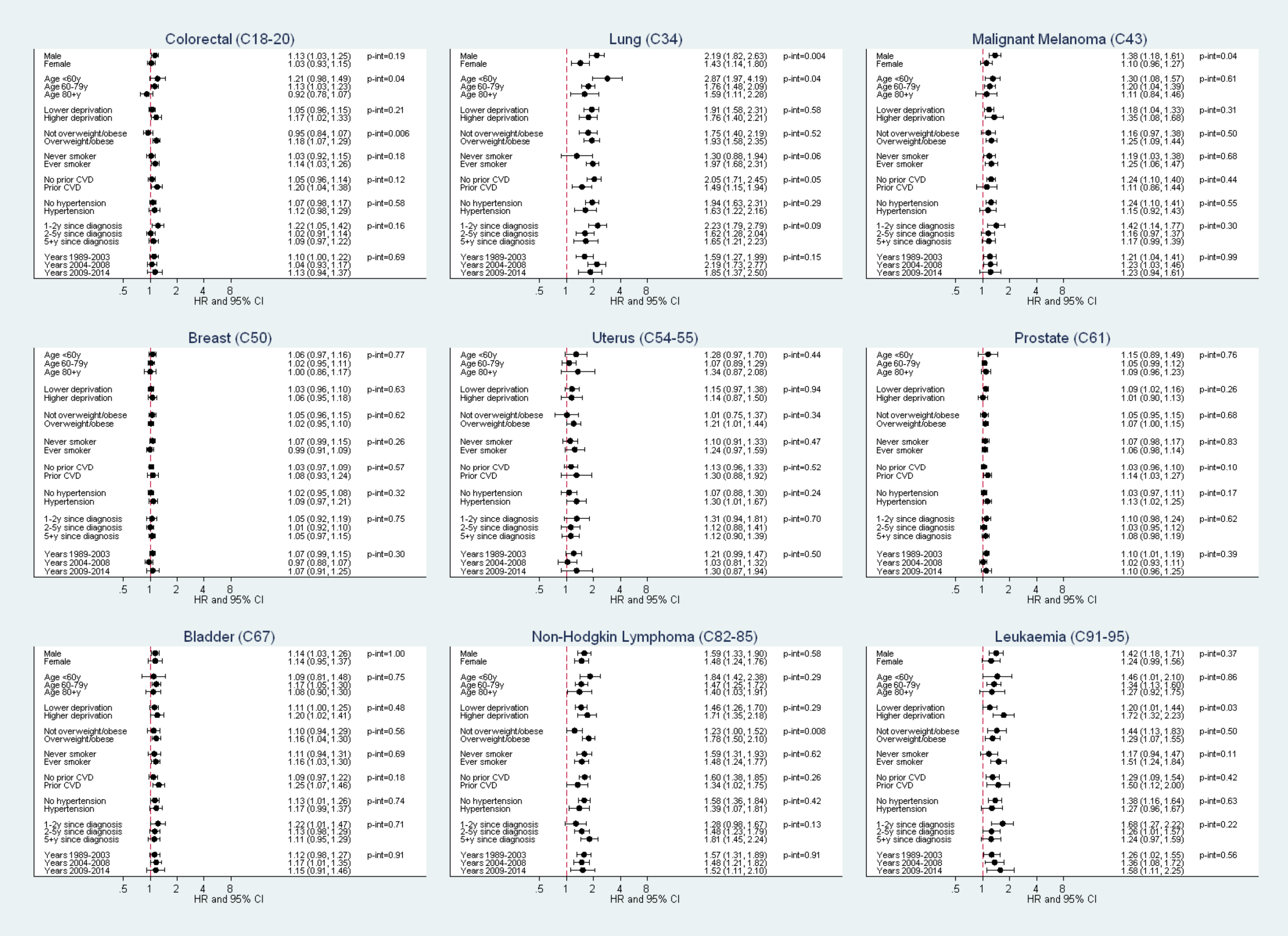

E) VENOUS THROMBOEMBOLISM

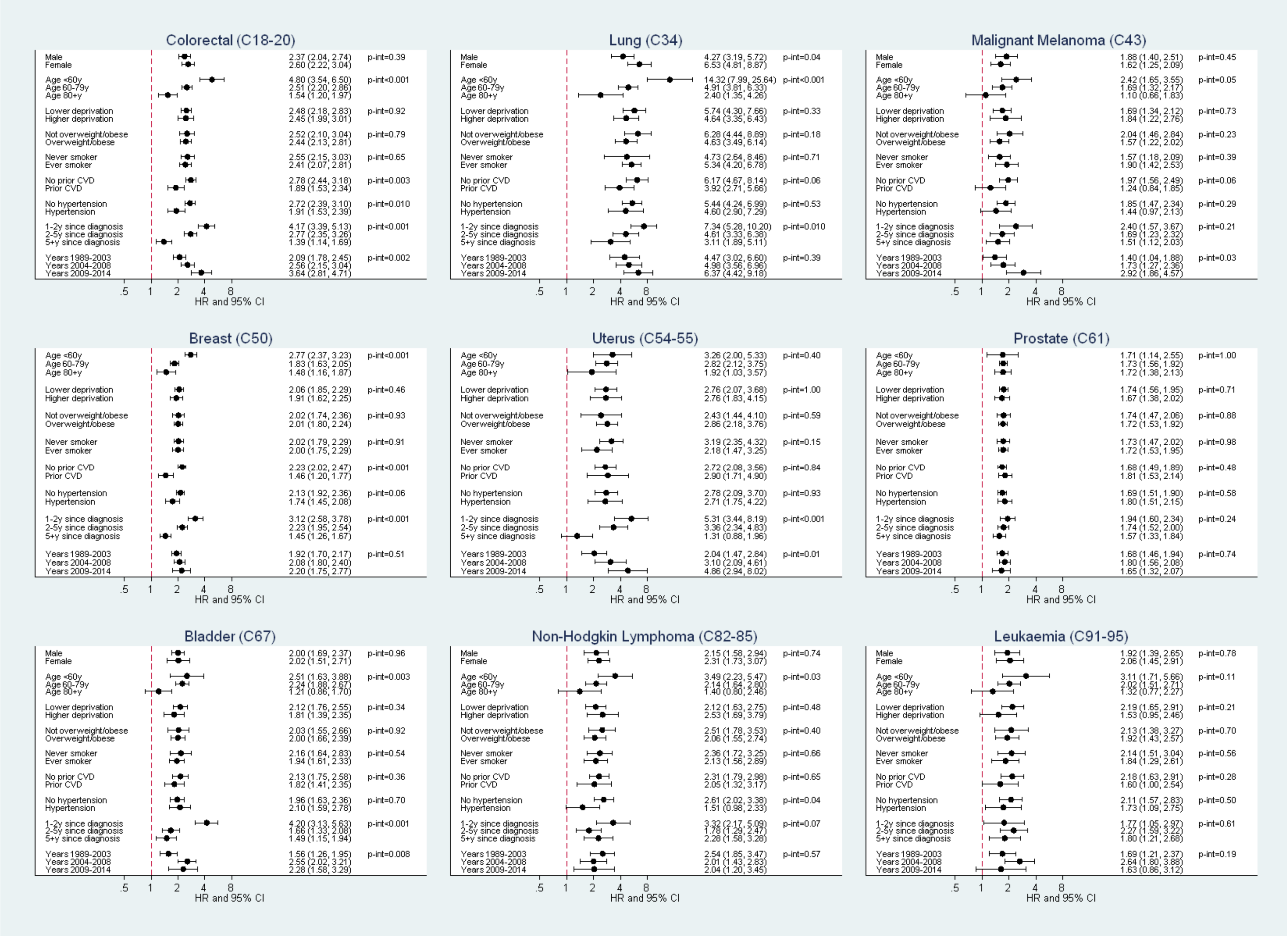

Figure B4: Absolute incidence of cardiovascular outcomes compared with expected incidence, by age group and time since diagnosis

A) Coronary artery disease

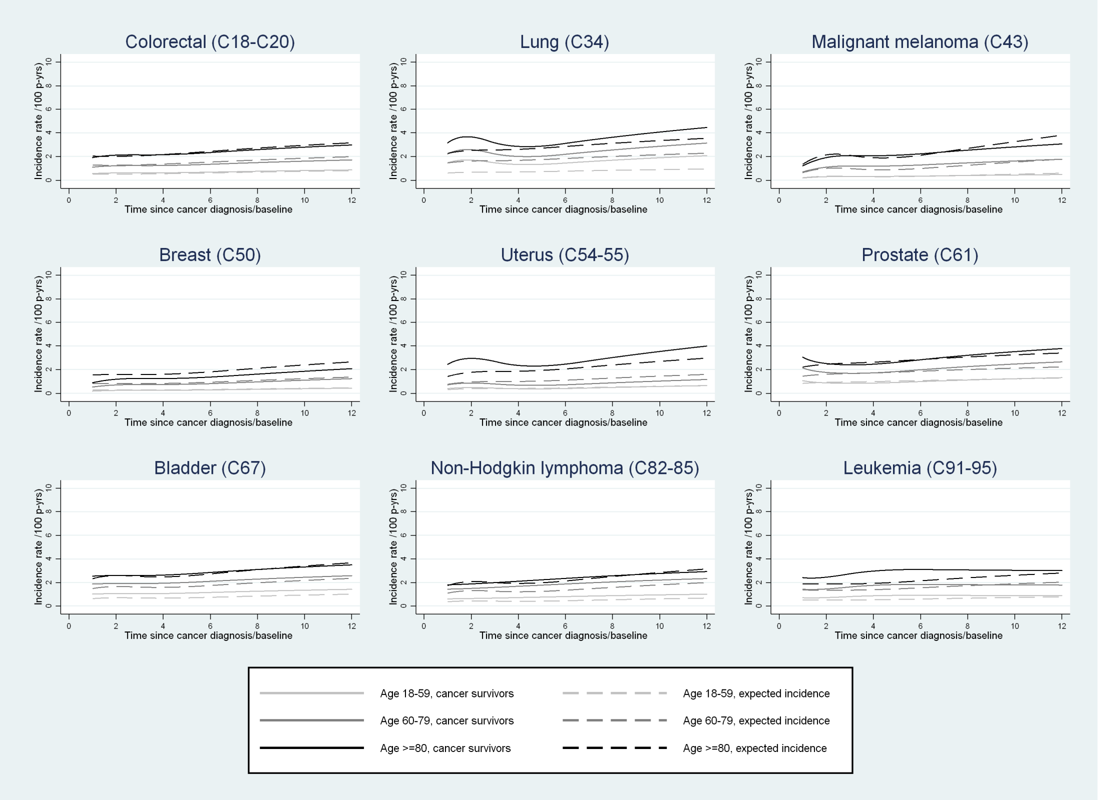

B) Arrhythmia

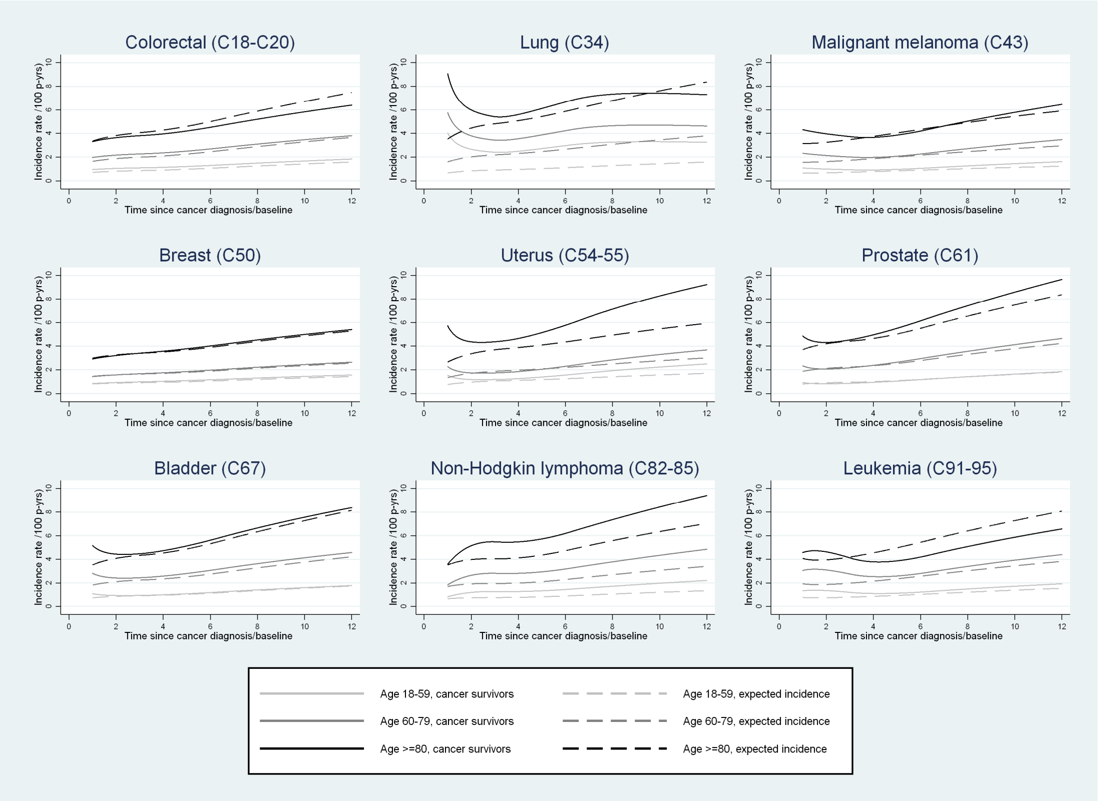

C) Heart failure

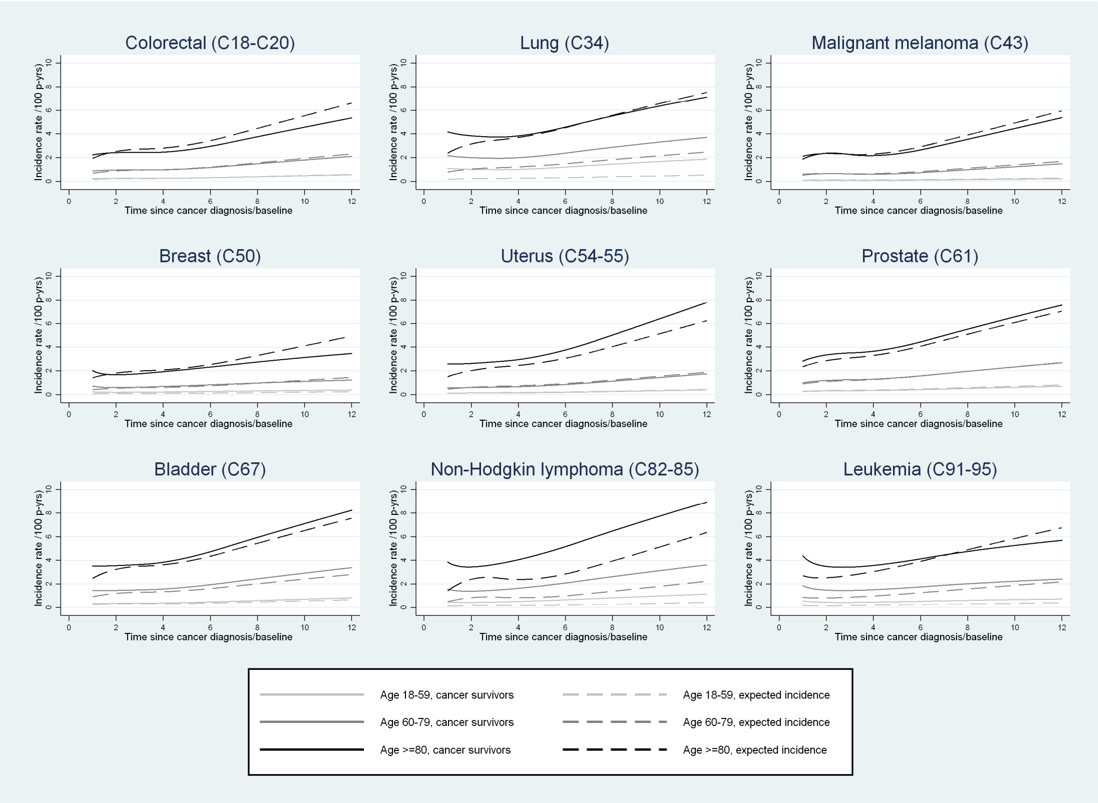

D) Stroke

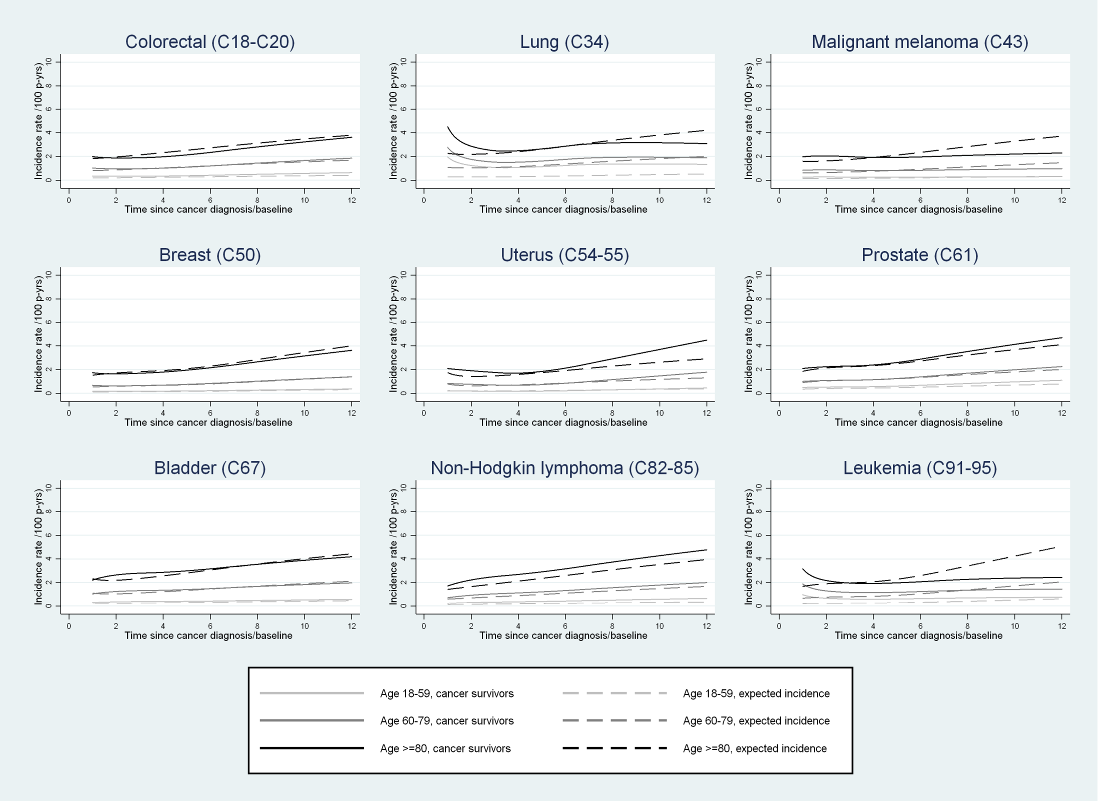

E) *Venous thromboembolism*

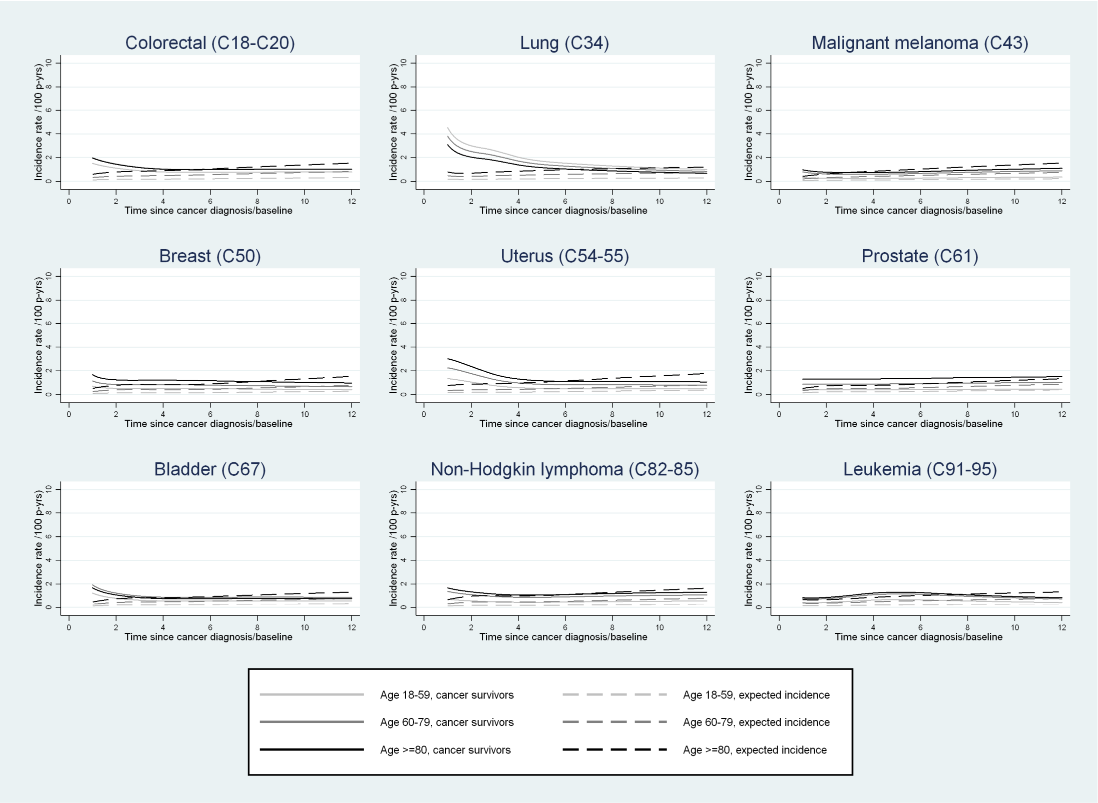

Note: Incidence in cancer survivors and expected incidence calculated by fitting flexible parametric survival models with exposure (cancer survivor vs control), covariates, and interaction between exposure and time since diagnosis, and predicting incidence for exposed/unexposed at the mean value of covariates in cancer survivors

Table B21: Cumulative incidence at 5 years of specific cardiovascular outcomes among cancers survivors and controls, in the presence of the competing risk of other-cause deaths

	Cumulative incidence of CVD outcome at 5 years from cancer diagnosis or equivalent date for controls					
	Cancer survivors	Controls	Cancer survivors	Controls	Cancer survivors	Controls
(Age in yrs)	*18-59*		*60-79*		*80+*	
**Arrhythmia**						
Colorectal (C18-C20)	3.4 (2.7, 4.3)	3.4 (3.1, 3.8)	7.5 (6.8, 8.2)	8.1 (7.7, 8.4)	12.7 (11.0, 14.4)	15.7 (14.9, 16.6)
Lung (C34)	5.8 (4.2, 7.8)	3.5 (2.9, 4.2)	8.0 (6.9, 9.2)	8.0 (7.5, 8.5)	11.3 (8.5, 14.5)	16.0 (14.4, 17.6)
Melanoma (C43)	3.3 (2.7, 4.1)	3.0 (2.7, 3.3)	8.4 (7.2, 9.7)	7.4 (6.9, 8.0)	15.8 (12.5, 19.4)	15.0 (13.5, 16.6)
Breast (C50)	3.6 (3.2, 4.0)	3.6 (3.4, 3.8)	6.5 (5.9, 7.1)	6.9 (6.6, 7.1)	13.7 (12.0, 15.5)	14.4 (13.6, 15.2)
Uterus (C54-55)	4.5 (3.1, 6.2)	3.8 (3.2, 4.5)	7.6 (6.2, 9.1)	7.1 (6.4, 7.7)	15.9 (10.8, 21.8)	14.7 (12.4, 17.1)
Prostate (C61)	3.5 (2.6, 4.7)	3.3 (2.9, 3.8)	8.6 (8.0, 9.2)	9.1 (8.8, 9.4)	15.8 (14.3, 17.4)	16.4 (15.7, 17.1)
Bladder (C67)	3.6 (2.4, 5.0)	3.5 (3.0, 4.1)	9.2 (8.3, 10.3)	8.5 (8.1, 9.0)	15.8 (13.6, 18.2)	16.2 (15.2, 17.3)
NHL (C82-85)	4.6 (3.4, 6.0)	2.9 (2.5, 3.4)	9.9 (8.4, 11.5)	8.2 (7.6, 8.8)	15.3 (11.7, 19.4)	15.3 (13.6, 17.2)
Leukaemia (C91-95)	3.8 (2.5, 5.5)	3.3 (2.7, 4.0)	9.9 (8.3, 11.6)	7.3 (6.7, 8.0)	13.9 (10.5, 17.8)	16.5 (14.8, 18.4)
**Coronary artery disease**						
Colorectal (C18-C20)	2.3 (1.7, 3.0)	1.9 (1.7, 2.2)	4.4 (3.9, 4.9)	5.6 (5.4, 5.9)	7.5 (6.2, 8.8)	8.4 (7.8, 9.0)
Lung (C34)	3.4 (2.2, 5.0)	2.0 (1.6, 2.5)	5.0 (4.2, 6.0)	5.7 (5.3, 6.2)	7.0 (4.8, 9.9)	9.2 (8.1, 10.5)
Melanoma (C43)	1.2 (0.8, 1.8)	1.0 (0.8, 1.2)	5.4 (4.5, 6.5)	4.5 (4.1, 5.0)	8.1 (5.7, 11.0)	9.2 (8.0, 10.4)
Breast (C50)	0.8 (0.7, 1.0)	1.0 (0.9, 1.1)	3.1 (2.7, 3.5)	3.9 (3.7, 4.1)	5.4 (4.4, 6.6)	7.6 (7.0, 8.2)
Uterus (C54-55)	1.4 (0.7, 2.5)	1.5 (1.2, 1.9)	3.4 (2.5, 4.4)	3.9 (3.4, 4.3)	10.9 (7.1, 15.7)	7.2 (5.7, 9.0)
Prostate (C61)	2.9 (2.1, 3.8)	3.5 (3.0, 3.9)	6.6 (6.1, 7.2)	6.6 (6.4, 6.8)	9.3 (8.2, 10.6)	10.1 (9.5, 10.7)
Bladder (C67)	3.4 (2.3, 4.8)	2.3 (1.9, 2.8)	7.5 (6.6, 8.5)	6.3 (5.9, 6.7)	8.6 (6.9, 10.4)	10.0 (9.1, 10.9)
NHL (C82-85)	1.9 (1.2, 2.9)	1.6 (1.3, 2.0)	6.3 (5.1, 7.6)	5.3 (4.8, 5.8)	7.6 (5.2, 10.7)	8.3 (7.0, 9.7)
Leukaemia (C91-95)	2.6 (1.5, 4.0)	1.8 (1.4, 2.4)	6.5 (5.2, 8.0)	6.0 (5.4, 6.7)	10.7 (7.6, 14.3)	8.7 (7.4, 10.1)
**Heart Failure /Cardiomyopathy**						
Colorectal (C18-C20)	0.7 (0.4, 1.2)	0.9 (0.7, 1.1)	3.9 (3.5, 4.4)	4.4 (4.2, 4.6)	9.7 (8.4, 11.1)	12.2 (11.5, 12.9)
Lung (C34)	2.2 (1.3, 3.5)	0.6 (0.4, 0.9)	4.9 (4.1, 5.8)	4.4 (4.1, 4.8)	8.8 (6.5, 11.6)	14.6 (13.2, 16.1)
Melanoma (C43)	0.4 (0.2, 0.8)	0.4 (0.3, 0.5)	2.9 (2.3, 3.7)	3.6 (3.3, 4.0)	12.9 (10.1, 15.9)	13.8 (12.5, 15.2)
Breast (C50)	0.9 (0.7, 1.1)	0.4 (0.3, 0.4)	2.9 (2.6, 3.3)	3.0 (2.8, 3.2)	10.7 (9.3, 12.2)	11.6 (10.9, 12.2)
Uterus (C54-55)	0.5 (0.2, 1.3)	0.7 (0.5, 1.0)	3.5 (2.7, 4.5)	2.9 (2.5, 3.3)	12.1 (8.1, 17.0)	10.9 (9.1, 12.9)
Prostate (C61)	1.1 (0.7, 1.8)	1.1 (0.9, 1.4)	5.2 (4.8, 5.6)	5.3 (5.1, 5.5)	13.8 (12.5, 15.2)	14.0 (13.4, 14.7)
Bladder (C67)	1.4 (0.8, 2.4)	1.1 (0.8, 1.4)	6.4 (5.7, 7.3)	5.2 (4.9, 5.6)	14.2 (12.2, 16.3)	14.5 (13.5, 15.4)
NHL (C82-85)	1.9 (1.2, 2.9)	0.4 (0.3, 0.6)	6.7 (5.6, 8.0)	4.1 (3.7, 4.6)	15.3 (12.0, 19.0)	11.4 (10.0, 12.9)
Leukaemia (C91-95)	1.9 (1.0, 3.1)	0.7 (0.4, 1.0)	6.4 (5.2, 7.8)	4.4 (3.9, 4.9)	15.7 (12.4, 19.4)	13.6 (12.1, 15.1)
**Stroke**						
Colorectal (C18-C20)	1.2 (0.8, 1.7)	0.8 (0.7, 1.0)	3.4 (3.0, 3.9)	4.0 (3.8, 4.2)	7.4 (6.3, 8.6)	8.4 (7.9, 9.0)
Lung (C34)	2.5 (1.5, 3.9)	0.9 (0.6, 1.2)	3.9 (3.2, 4.7)	4.1 (3.8, 4.5)	5.4 (3.7, 7.4)	8.5 (7.5, 9.6)
Melanoma (C43)	0.8 (0.5, 1.3)	0.5 (0.4, 0.7)	3.9 (3.1, 4.7)	3.2 (2.9, 3.5)	9.1 (6.9, 11.7)	9.3 (8.2, 10.5)
Breast (C50)	0.6 (0.5, 0.8)	0.6 (0.5, 0.6)	2.7 (2.4, 3.1)	2.9 (2.7, 3.0)	8.2 (7.0, 9.5)	8.9 (8.3, 9.5)
Uterus (C54-55)	0.5 (0.2, 1.1)	0.8 (0.6, 1.1)	3.1 (2.4, 4.1)	2.8 (2.5, 3.2)	7.9 (4.9, 11.8)	7.7 (6.2, 9.4)
Prostate (C61)	1.9 (1.3, 2.7)	1.4 (1.1, 1.7)	4.1 (3.8, 4.5)	4.3 (4.1, 4.4)	8.1 (7.1, 9.2)	9.0 (8.5, 9.5)
Bladder (C67)	1.0 (0.4, 1.8)	0.9 (0.7, 1.3)	4.4 (3.8, 5.1)	4.2 (3.9, 4.5)	9.8 (8.2, 11.6)	9.0 (8.3, 9.8)
NHL (C82-85)	1.3 (0.7, 2.1)	0.6 (0.4, 0.8)	3.9 (3.1, 4.9)	3.6 (3.2, 4.0)	8.4 (5.9, 11.5)	7.6 (6.5, 8.9)
Leukaemia (C91-95)	1.7 (0.9, 2.9)	0.8 (0.5, 1.1)	4.6 (3.6, 5.8)	3.2 (2.8, 3.7)	7.9 (5.6, 10.7)	8.7 (7.5, 10.0)
**Venous Thromboembolism**						
Colorectal (C18-C20)	3.3 (2.6, 4.1)	0.6 (0.5, 0.8)	4.5 (4.0, 5.0)	1.8 (1.6, 1.9)	4.3 (3.5, 5.3)	3.2 (2.8, 3.5)
Lung (C34)	6.4 (4.7, 8.3)	0.6 (0.4, 1.0)	5.0 (4.2, 5.8)	2.0 (1.8, 2.2)	3.2 (2.0, 4.8)	2.6 (2.1, 3.3)
Melanoma (C43)	0.8 (0.5, 1.2)	0.4 (0.3, 0.6)	2.8 (2.1, 3.5)	1.4 (1.2, 1.7)	2.4 (1.4, 3.9)	2.7 (2.2, 3.4)
Breast (C50)	1.9 (1.7, 2.2)	0.5 (0.5, 0.6)	3.8 (3.4, 4.2)	1.7 (1.6, 1.8)	4.5 (3.6, 5.4)	3.2 (2.9, 3.6)
Uterus (C54-55)	3.0 (2.0, 4.3)	0.7 (0.4, 1.0)	5.1 (4.0, 6.2)	1.4 (1.1, 1.6)	6.0 (3.5, 9.3)	3.4 (2.5, 4.6)
Prostate (C61)	1.7 (1.1, 2.5)	1.0 (0.7, 1.2)	3.2 (2.9, 3.5)	1.9 (1.8, 2.1)	3.9 (3.2, 4.7)	2.6 (2.3, 2.8)
Bladder (C67)	2.7 (1.8, 4.0)	0.8 (0.5, 1.1)	4.1 (3.5, 4.8)	1.8 (1.6, 2.0)	2.9 (2.1, 3.9)	2.9 (2.5, 3.3)
NHL (C82-85)	2.1 (1.4, 3.1)	0.6 (0.4, 0.8)	3.4 (2.6, 4.3)	2.0 (1.7, 2.3)	4.5 (2.8, 6.7)	3.4 (2.6, 4.3)
Leukaemia (C91-95)	2.1 (1.2, 3.5)	0.5 (0.3, 0.9)	3.6 (2.7, 4.7)	1.9 (1.6, 2.2)	3.0 (1.7, 4.9)	3.2 (2.5, 4.0)

Note: cumulative incidence calculated in the presence of the competing risk of other-cause death; entry into the analysis was at the index date (first anniversary of cancer diagnosis of the cancer survivor in the matched set) so events in the first year after cancer diagnosis are not included.

Figure B5: Associations between site-specific cancer survivorship and specific CVD outcomes, stratified by receipt of chemotherapy/radiotherapy

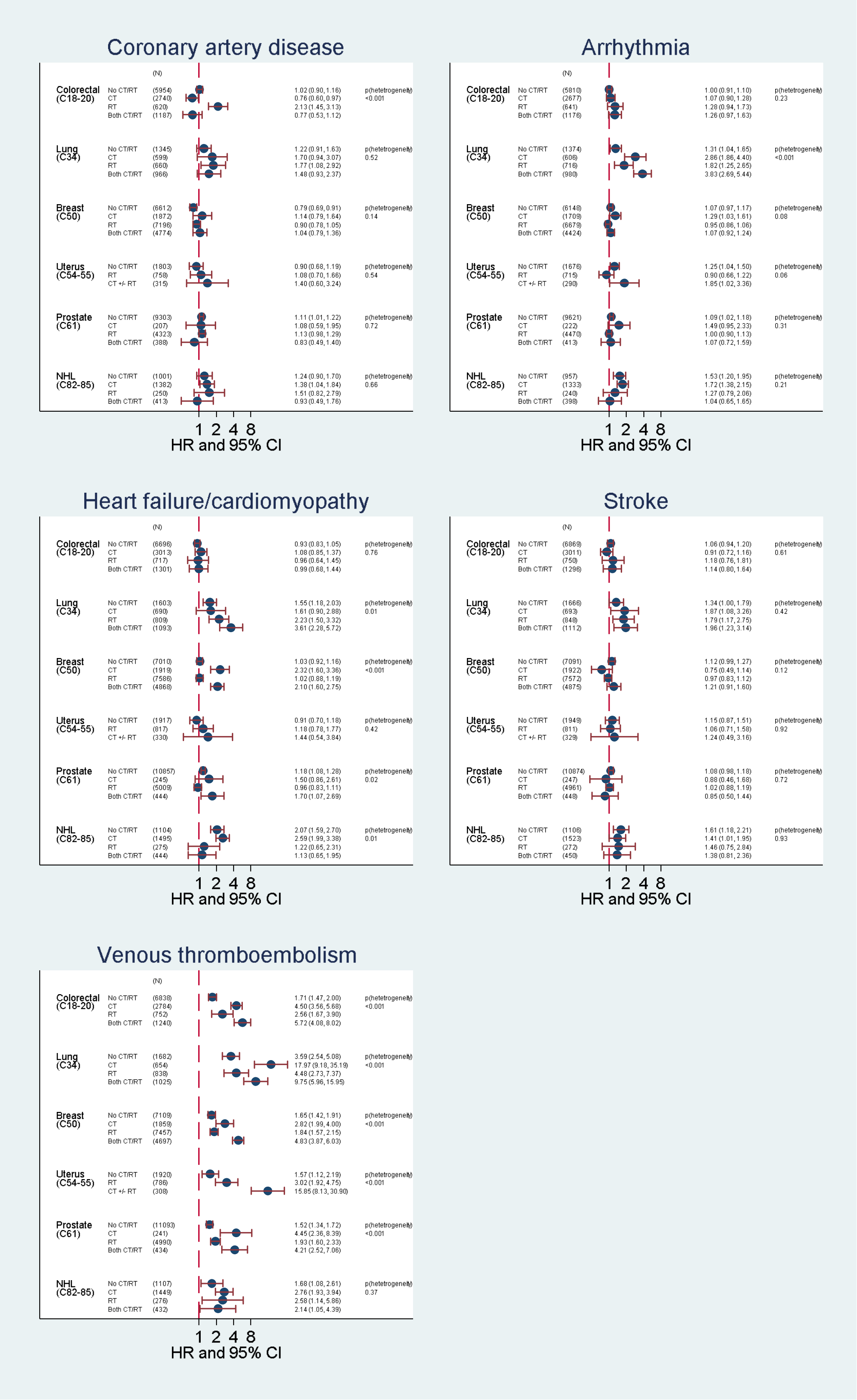

Notes: NHL = non-Hodgkin lymphoma. CT = chemotherapy; RT = radiotherapy. For cancer of uterus, the chemotherapy and "both" groups were combined due to small numbers.

Figure B6: Estimated relative risks of cardiovascular disease in cancer survivors compared to controls from sensitivity analyses

A-B) Coronary artery disease and arrhythmia

Figure B6A to B: Adjusted sensitivity models

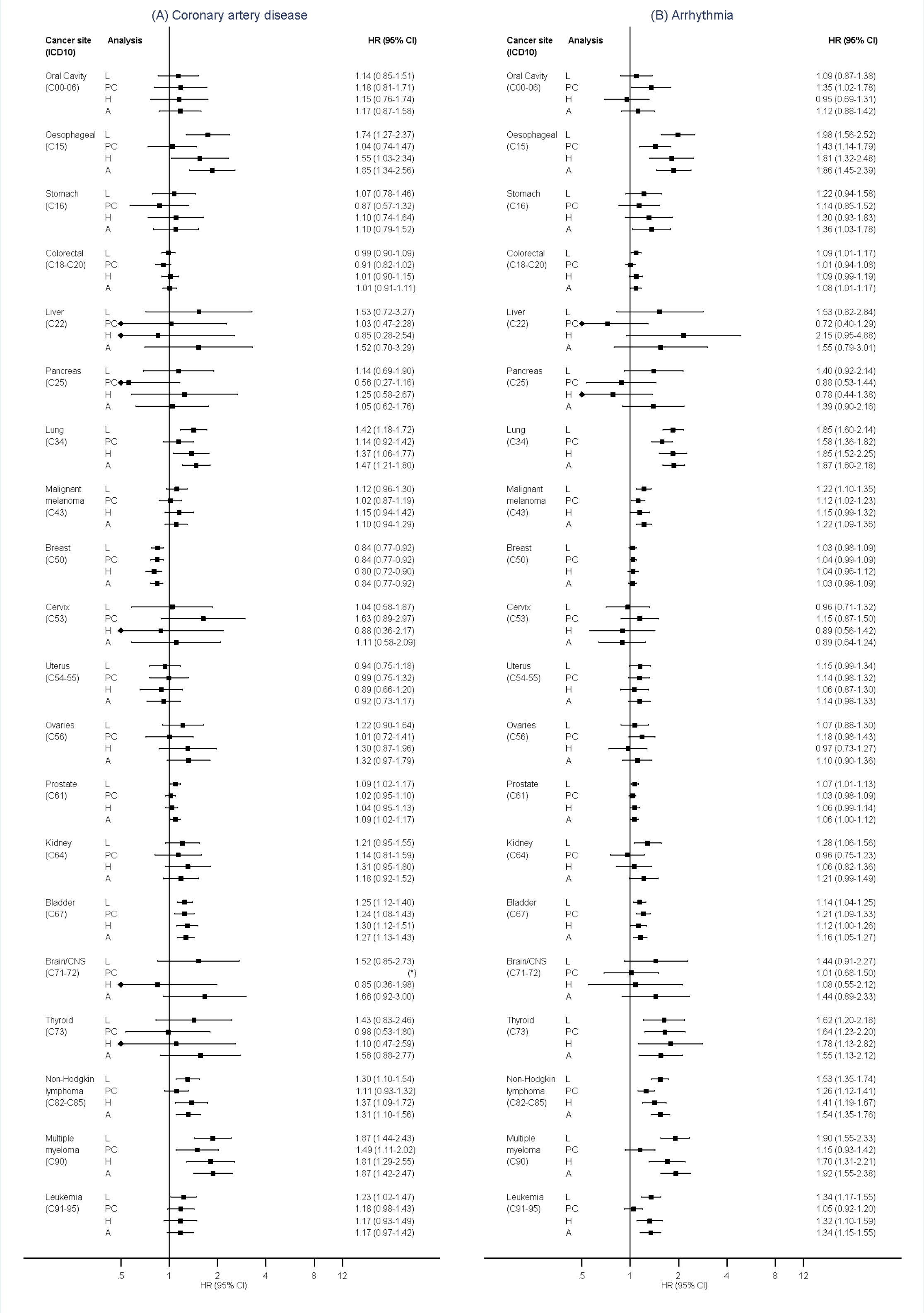

(*) too few events for estimation HR = hazard ratio, CI = confidence interval
L = Primary analysis cohort (linked data), PC = Primary care only cohort
H = More specific hypertension definition, A = Categorical alcohol use definition

C-D) Heart failure/cardiomyopathy and stroke

Figure B6C to D: Adjusted sensitivity models

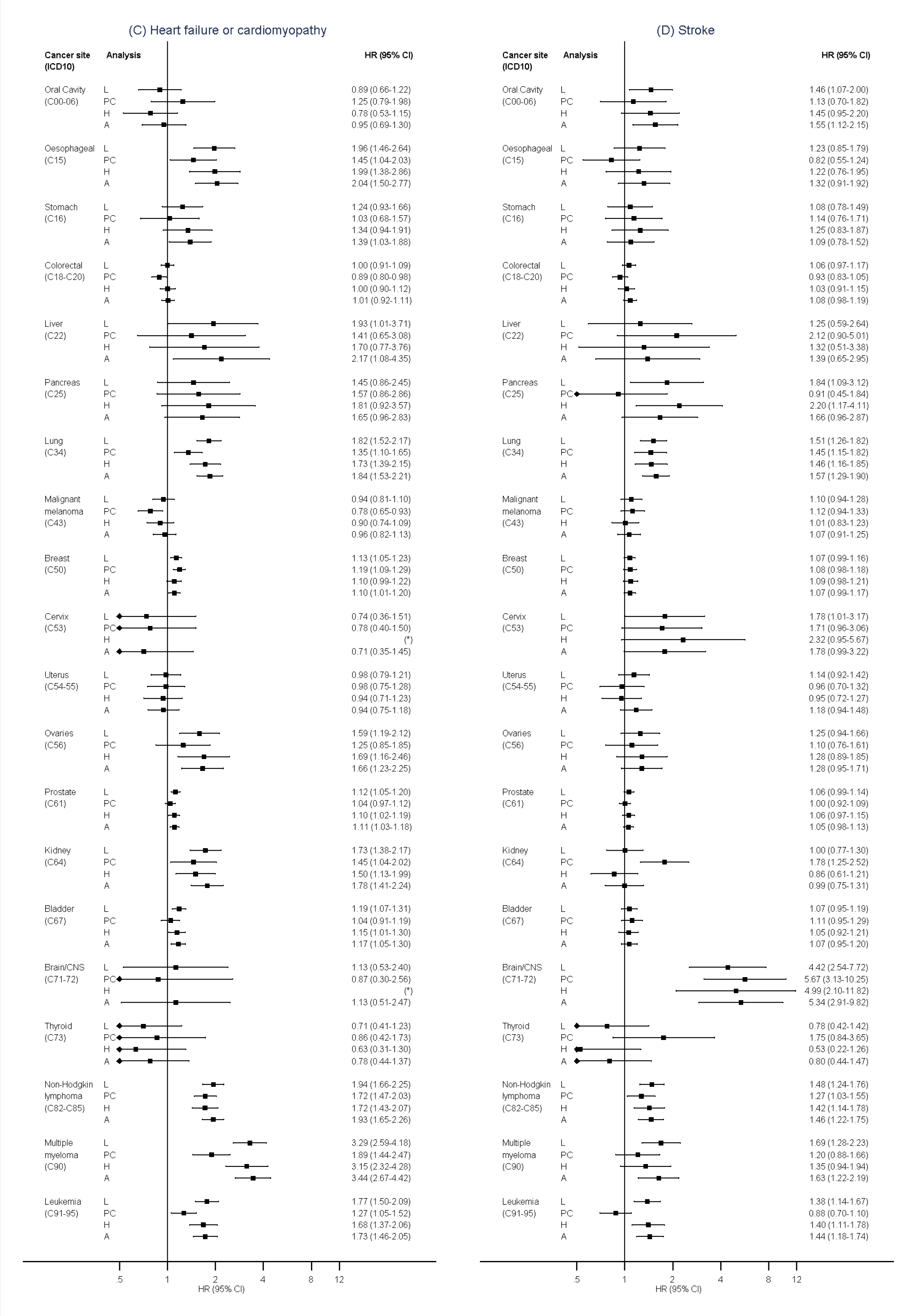

(*) too few events for estimation HR = hazard ratio, CI = confidence interval
L = Primary analysis cohort (linked data), PC = Primary care only cohort
H = More specific hypertension definition, A = Categorical alcohol use definition

E-F) Peripheral vascular disease and venous thromboembolism

Figure B6E to F: Adjusted sensitivity models

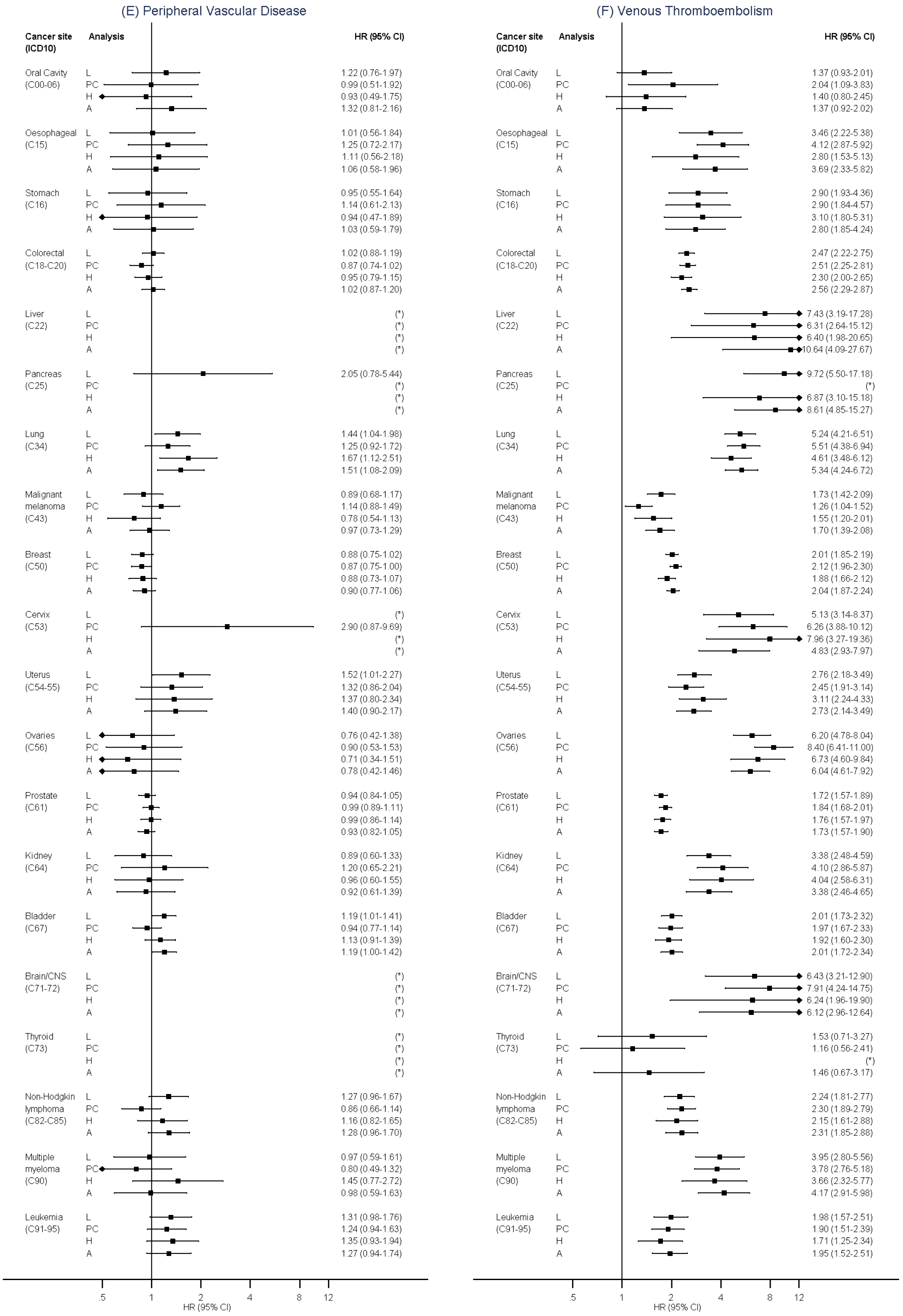

(*) too few events for estimation HR = hazard ratio, CI = confidence interval
L = Primary analysis cohort (linked data), PC = Primary care only cohort
H = More specific hypertension definition, A = Categorical alcohol use definition

G-H) Pericarditis and valvular heart disease

Figure B6G to H: Adjusted sensitivity models

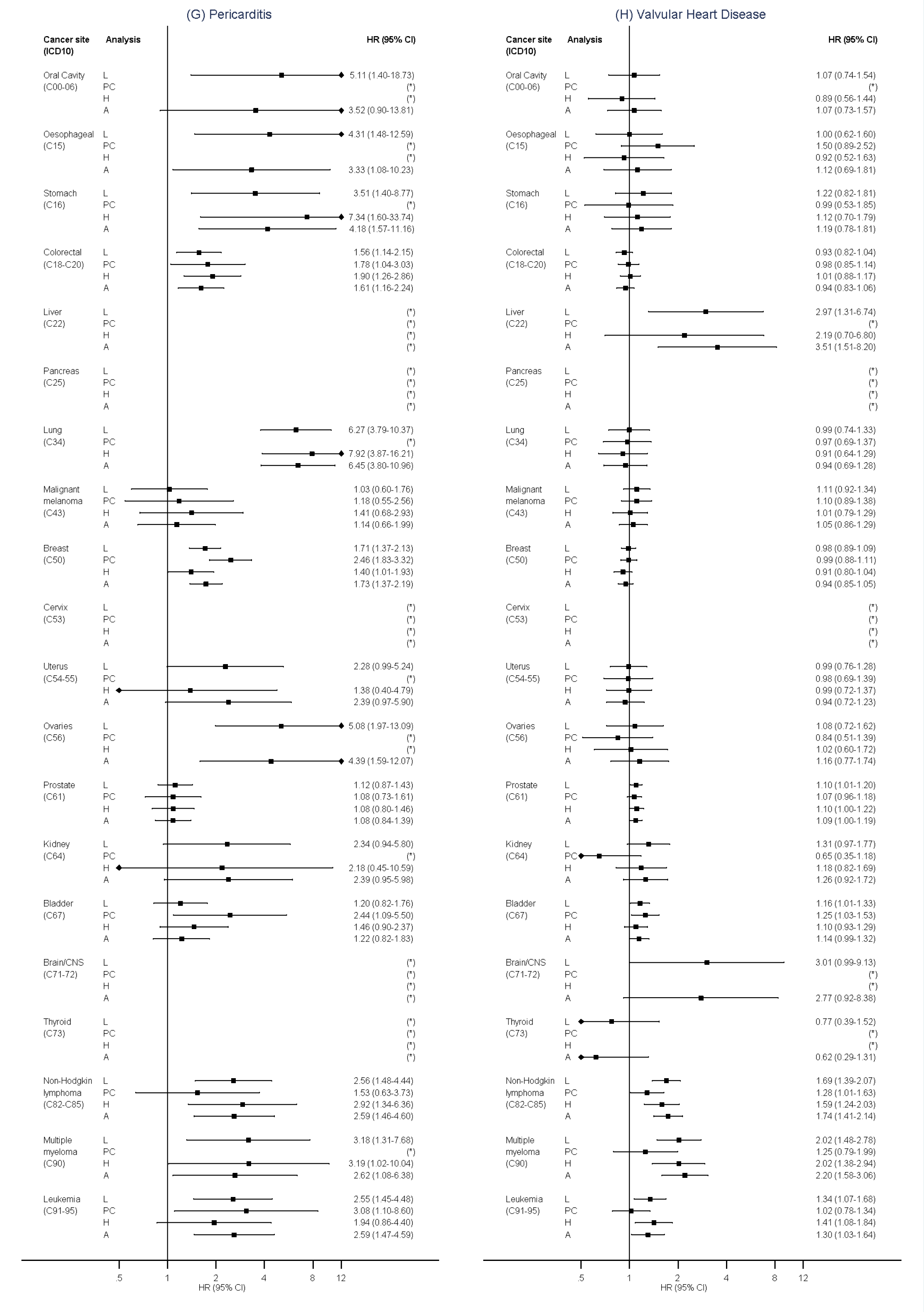

(*) too few events for estimation HR = hazard ratio, CI = confidence interval
L = Primary analysis cohort (linked data), PC = Primary care only cohort
H = More specific hypertension definition, A = Categorical alcohol use definition

Part C – Systematic review of previous studies comparing specific CVD outcomes in site-specific cancer survivors vs non-cancer or general population controls

Databases searched: Pubmed and OVID Medline

Search strategies

A full title search (search 1) was combined with a broader title/abstract search of key journals (search 2), as below.

	**Search 1**	**Search 2**
String search for specific and composite cardiovascular diseases or comorbidity related terms	In title	In title, abstract
AND String search for cancer related terms	In title	In title, abstract
AND Mesh and string searches for epidemiology studies, reviews and guidelines	In title	In title, abstract
AND high impact oncology, cardiology or general medical journal	N/A	Journal title
English language abstracts in the last 10 years (2008-2018)	Filter	Filter

Inclusion criteria: Articles were included in the full text screen if they provided relative estimates comparing risk of cardiovascular disease or mortality in adult cancer survivors with cancer-free controls.

Exclusion criteria: Articles were excluded if they did not report estimates for site-specific cancers and specific cardiovascular diseases included in our study; included young adults without stratification on age group.

Extraction of study characteristics: The following were extracted from each included study where information was available: data source / setting, cancer site(s), control group, age profile, specific cardiovascular outcomes investigated, adjustment for covariates, handing of prior cardiovascular disease, lag from cancer diagnosis to follow-up and average follow-up.

Extraction of study results: Relative risk estimates were extracted directly.

Processing of study results: Descriptive characteristics of the included studies are presented in table form. Results of all studies are summarised graphically.

Figure C1: Review of previous studies: flow chart of the systematic review search process

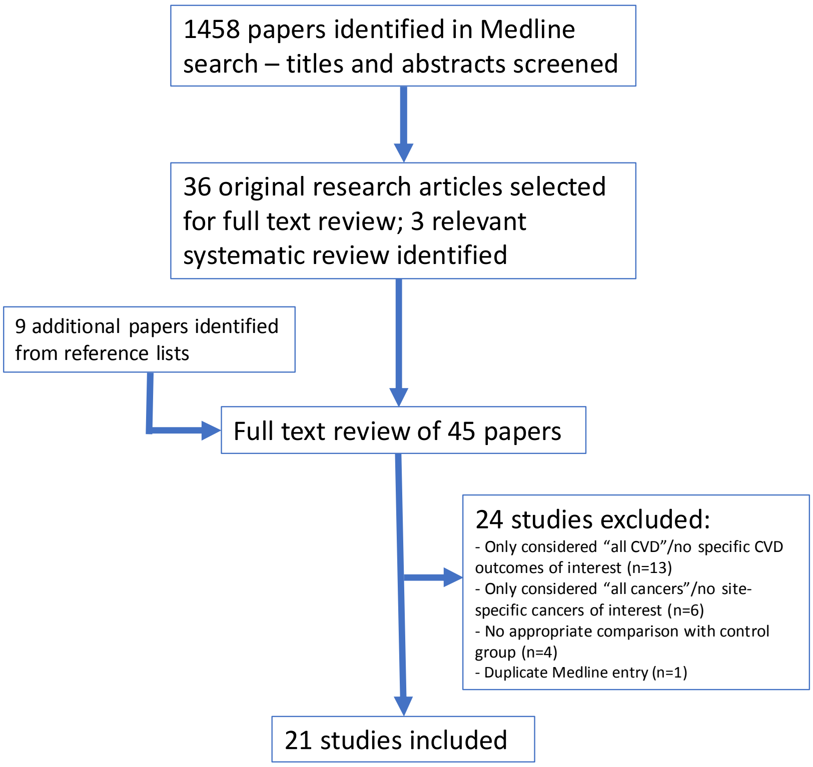

Table C1: Review of previous studies - study characteristics

Study (author, year)	Data source/Setting	Cancer site(s)	Control group	Age profile	Specific CVD outcomes investigated*	Adjustment for covariates	Handling of prior CVD	Lag from cancer diagnosis to start follow-up?	Study size	Av follow-up
**Abdel-Qadir, 2019**[1]	Linked claims and cancer registration data in Ontario; Canada, 2005-15	Breast (early stage)	Age-matched cancer free controls (3:1)	Median 61y	Heart failure, ischaemic heart disease, cerebrovascular disease, arrhythmia	Age (matched), calendar year, rural residence, median neighborhood income, history of specific CVDs, diabetes, hypertension, chronic obstructive pulmonary disease, chronic kidney disease,	Adjusted for in modelling	No	78,318 exposed; 234,954 controls	5.7y (exposed)
**Ameijide, 2019**[2]	Cancer registry linked to death registry data from Northern Spain; 1985-2004	Breast	General Catalonia population (indirect comparison with Catalonia cause-specific mortality rates)	Restricted to ages 15-84; mean /median not reported	Cause specific mortality including CVD deaths (composite); 9 individual CVD mortality outcomes including MI, cerebrovascular, heart failure	SMRs calculated based on age and calendar year specific death rates using age and calendar-year specific death rates	No restrictions mentioned	No	10,195 exposed	12.4y
**Armenian, 2016**[3]	Kaiser Permanente Southern California members; US; 2000-2007	14 specific sites	Age, sex and region-matched cancer-free controls	Median 60y (range 40-96)	CVD (composite); plus exploratory analysis of cardiomyopathy/heart failure, ischaemic heart disease, stroke	age, sex, race/ethnicity, smoking, overweight/obesity; time-updated diabetes, hypertension, dyslipidaemia	Those with any prior CVD excluded	2y	36,232 exposed; 73,545 controls	4.4y (exposed); 4.5y (controls)
**Boekel, 2016**[4]	Netherlands Cancer registry linked to CVD and death registries; Netherlands 1989-2005	Breast (stage I-III surgically treated only)	General Dutch female population (indirect comparison)	Restricted to age <75; Median age category 49-59y	CVD (composite); MI, valvular dysfunction, pericarditis, cardiomyopathy, congestive heart failure, arrythmia, cerebrovascular disease	SMRs calculated accounting for age, calendar period, follow-up interval	No restrictions mentioned	5y	70,230 exposed	9y (range 0-21)

**Boerman, 2014**[5]	Data from 10 general practices; north Netherlands; 1970-2007	Breast (those treated with curative intent only)	3 general practice and age-matched cancer-free controls per exposed patient	Restricted to age <80y; Median 56y (radio-therapy group), 47y (chemo-therapy group), 54y (controls)	congestive heart failure, vascular cardiac diseases (inluding cerebrovascular)	age at diagnosis, prior CVD, CVD risk factors	No restrictions; prior CVD included in adjustments	No	561 exposed; 1,635 controls	9y (range 5-57)
**Brand, 2017**[6]	Stockholm breast cancer registry linked to other national registry datasets; Sweden 2001-2008	Breast	Age-matched general population controls	Restricted to ages 25-75; mean 57y	Venous thromboembolism	matched on age; adjusted for time since index only	No restrictions mentioned	No	8,338 exposed; 39,013 controls	7.2y (exposed); 5.9y (controls)
**Bright, 2017**[7]	Teenage and Young Adult Cancer Survivor Study linked to Hospital Episodes Statistics, England, 1971-2006	All cancers combined, central nervous system cancers; leukaemia	General UK population (indirect comparison)	Restricted to 15-39y; median age group 30-34y	Any cerebrovascular event, cerebral haemorrhage, cerebral infarction	SMRs (for hospitalisation) calculated accounting for age, sex, calendar period	No restrictions mentioned	5y	178,962 exposed	11.3y
**Chang, 2018**[8]	National Health Insurance Service cohort, South Korea, 2002-2013	Breast (radiotherapy-treated only)	Age, smoking and comorbidity matched controls without cancer	Restricted to age >=40y	Acute coronary events (in competing risks analysis)	Charlson index, smoking, hypertension, BMI, cholesterol, exercise, residential area, income, disability	No restrictions mentioned	Follow-up started at end of radio-therapy	1,015 exposed; 8,120 controls	6.1y
**Cronin-Fenton, 2010**[9]	Linked national cancer, hospitalisation and civil registries;	All cancers combined and	Birth year, county and sex-matched	Median age group 60-69	Venous thromboembolism	Matched on age, sex, county	No restrictions mentioned	No	57,591 exposed;	1.23y (exposed);

	Denmark, 1997-2006	20 individual sites	controls without cancer (5:1)						287,476 controls	3.46y (controls)
**Henson, 2016**[10]	Teenage and Young Adult Cancer Survivor Study linked to Hospital Episodes Statistics, England, 1971-2006	All cancers combined, and 18 site-specific cancers	General UK population (indirect comparison)	Restricted to 15-39y; median age group 30-34y	All cardiac disease, ischaemic heart disease, valvular disease, cardiomyopathy/heart failure	SMRs (for hospitalisation) calculated accounting for age, sex, calendar period	No restrictions mentioned	5y	200,945 exposed	14.3y
**Jordan, 2014**[11]	Women in the BOWI multisite cohort study, recruited from Cancer Research Network managed care systems; USA, 1990-1994	Breast	Age and health system- matched cancer-free controls	Restricted to age >=65y; median age group 75-79y	Myocardial infarction, congestive heart failure, peripheral vascular disease, cerebrovascular disease	Age, health system, existence of any prevalent comorbidity		5y	1,361 exposed; 1,361 controls	3.3y (exposed); 3.7y (controls)
**Kenzik, 2018**[12]	SEER-Medicare national cancer registry linked to Medicare claims; US, 2000-11	Colorectal cancer	5% non-cancer sample of Medicare data	Restricted to ≥65y. Median age 78y (range 66-106)	Congestive heart failure, CVD	Age, sex, race, comorbidity	Those with prior heart failure or CVD excluded		72,408 exposed; 72,408 controls	5y
**Kero, 2014**[13]	Finnish cancer registry linked to hospital registry; Finland, 1975-2004	All combined, leukaemia, HL NHL, CNS, renal, bone, soft tissue, thyroid, melanoma, colon, breast, testicular	Sibling controls	Restricted to age <35y (20 to 35 age group reported here)	Cardiomyopathy/cardiac insufficiency, atherosclerosis/brain vascular thrombosis, myocardial ischaemia/cardiac ischaemia, cardiac arrhythmia	Calendar time, birth decade, gender	Not mentioned	5y	9,401 cancer survivors (at ages 20-34y); 43,392 siblings	Not reported

**O'Farrell, 2015**[14]	Swedish national prescription and linked data; Sweden, 2006-12	Prostate	Male age- and county-matched general population controls (5:1)	Mean 75y	CVD (composite), ischaemic heart disease, arrhythmia, heart failure, stroke	Adjusted for time-updated proxies for disease progression (transurethral resection, palliative radiotherapy, nephrostomy)	Sensitivity analysis excluding if prior CVD at baseline	Follow -up began at start of androgen deprivation therapy	41,362 exposed; 187,785 controls	4.0y (exposed); 4.4y (controls)
**Pajamaki, 2018**[15]	2 Finnish University hospitals, Finland; 1981-2002	Differentiated thyroid carcinoma (DTC)	Age-, gender- and place of residence-matched controls	Mean 49y	CVD (composite), hypertension, arrhythmia, atrial fibrillation, vascular disease, coronary artery disease, cerebrovascular disease, heart failure, valvular diseases and cardiomyopathies, pulmonary artery diseases	Matched on age, sex, geographical location; adjusted for baseline CVD	Included in adjustments	No	901 exposed; 4,485 controls	18.8y (exposed); 19.0y (controls)
**Prasad, 2012**[16]	Finnish cancer registry, Finland, 1966-99	All cancer, 14 specific cancers (only lymphoma, CNS tumour stratified by paediatric/adult)	General Finnish population (indirect comparison)	Restricted to age<35y	CVD mortality, ischaemia mortality, cerebrovascular mortality	SMRs calculated accounting for age, sex, calendar period	Not mentioned	5y	9,245 exposed	15.9y (exposed)
**Riihimäki, 2012**[17]	Swedish Family-Cancer Database, Sweden, 1987-2006	Breast	All women in the database without breast cancer		Cerebrovascular deaths, myocardial infarction deaths, heart failure deaths, arterial disease deaths, pulmonary circulation deaths	Socioeconomic status, geographic region	Not mentioned	No	122,000 exposed; 3.55 million controls	Not reported
**Rugbjerg, 2014**[18]	Danish Cancer Registry linked to hospital data; Denmark, 1943-2009	Cervix, testis, breast, melanoma, brain, Hodgkin lymphoma, non-Hodgkin lymphoma,	Sex and year of birth-matched general population controls (5:1) obtained and used to calculate reference	Restricted to 15-39y; median age group 30-34y	35 specific CVD outcomes covering the circulatory diseases ICD-10 chapter	Standardised morbidity ratios accounted for age, sex, calendar period	Those with any prior CVD excluded	1y	43,153 exposed; 255,513 controls	

		ovary, thyroid, leukaemia	hospitalisation rates							
**Salz, 2017**[19]	Linked Danish population-based registries, Denmark 2000-10	Aggressive Non-Hodgkin lymphoma	Age- and sex-matched general population controls (3:1)	>=15	Heart failure	Matched on age and sex	Those with prior heart failure excluded	0.75y	2,508 exposed; 7,399 controls	2.5y (exposed); 4.1y (controls)
**Shin, 2018**[20]	Korean National Health Insurance Database; South Korea, 2004-11	Gastric cancer (requiring surgery)	Propensity score-matched non-cancer controls (1:1)	Mean age 57.9y	Coronary heart disease, ischaemic stroke	Propensity score included age, sex, residence, income, disability, hypertension, diabetes, dyslipidemia.	Those with prior ischaemic stroke excluded	Not mentioned, but those with <2y follow-up excluded	98,936 exposed; 98,936 controls	5.4y (exposed); 5.5y (controls)
**Soisson, 2018**[21]	Utah Population Database linked to SEER Cancer Registry; USA, 1997-2012	Endometrial	Age-matched women from the general population	>=18	47 specific CVD outcomes at various levels of granularity	Age, body mass index, Charlson comorbidity index, race	Not mentioned	1y	2,648 exposed; 10,503 controls	Median follow-up category 5-10y

Figure C2: Risk of CVD outcomes in cancer survivors vs controls in previous studies and present study

A) Arrhythmia

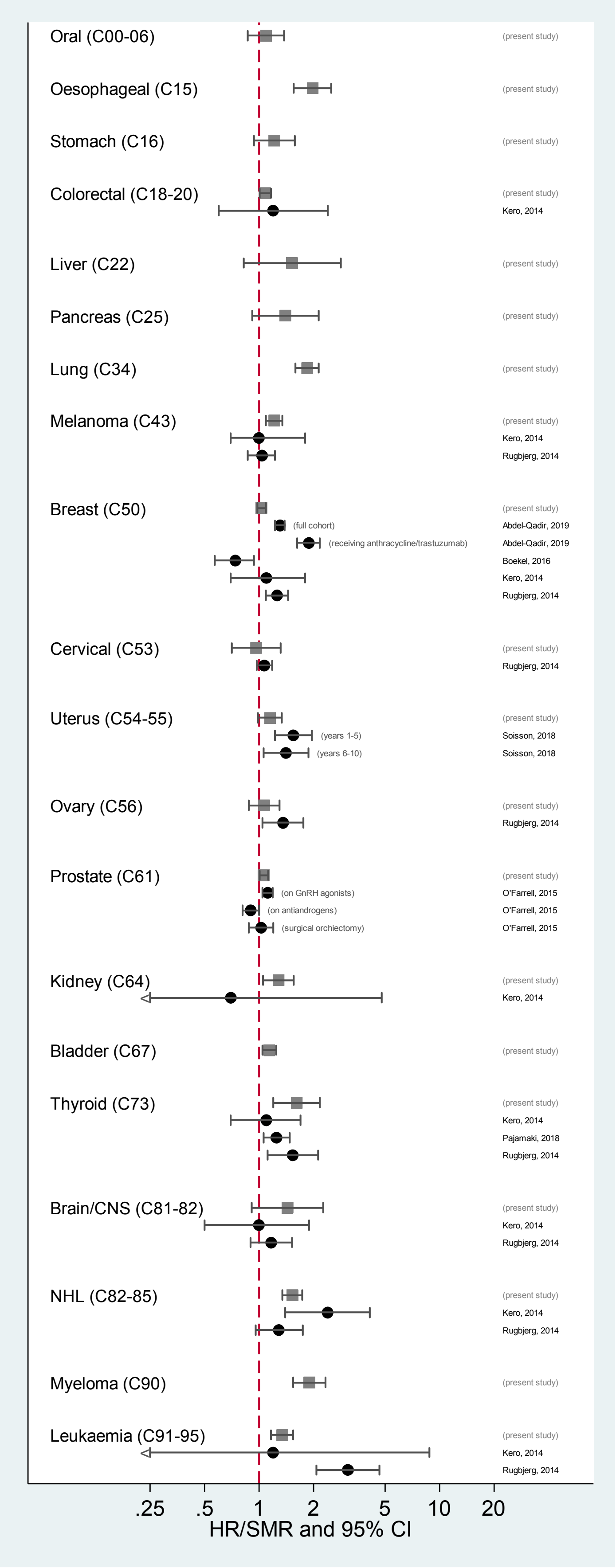

B) Coronary artery disease

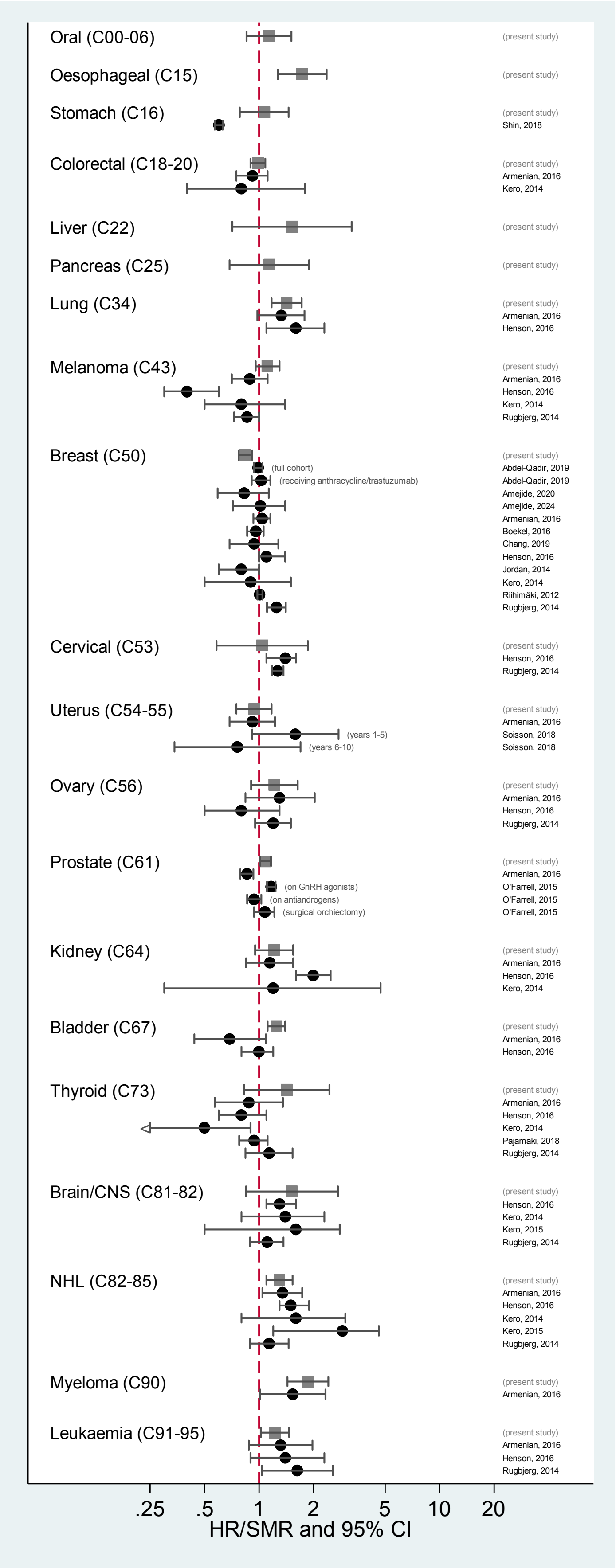

C) Heart failure/cardiomyopathy

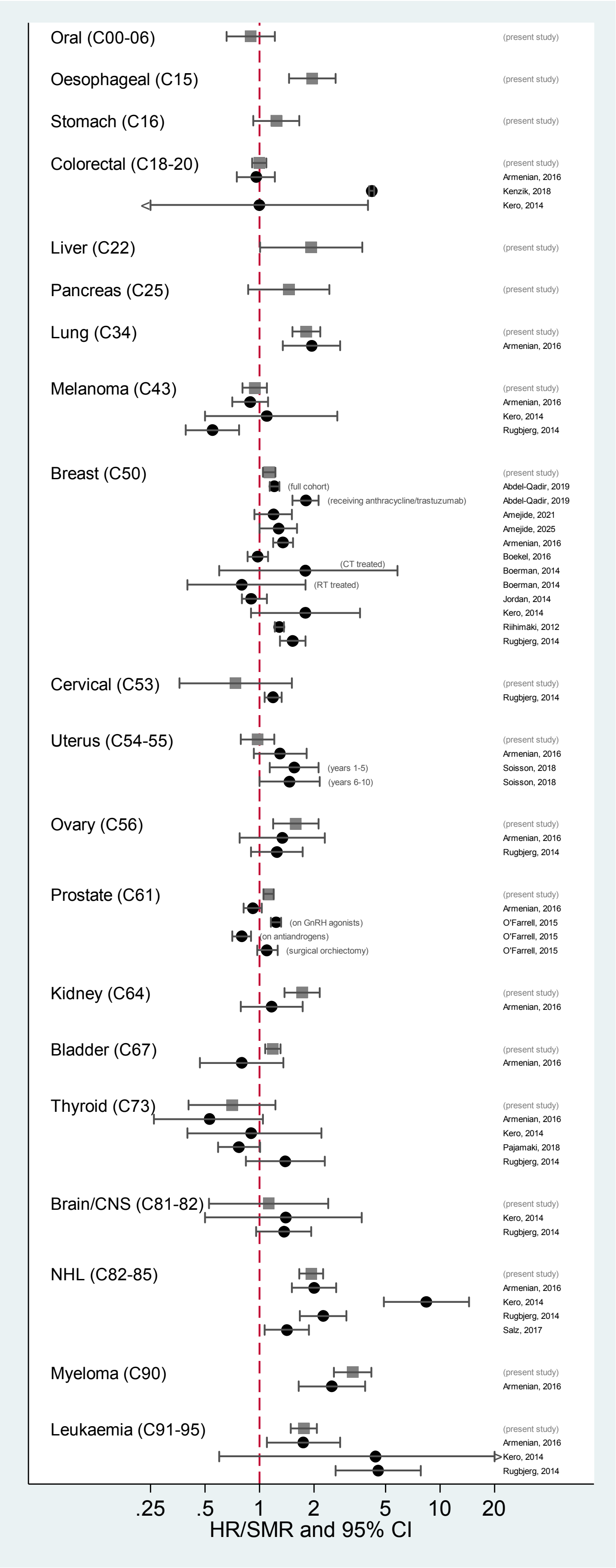

D) Pericarditis

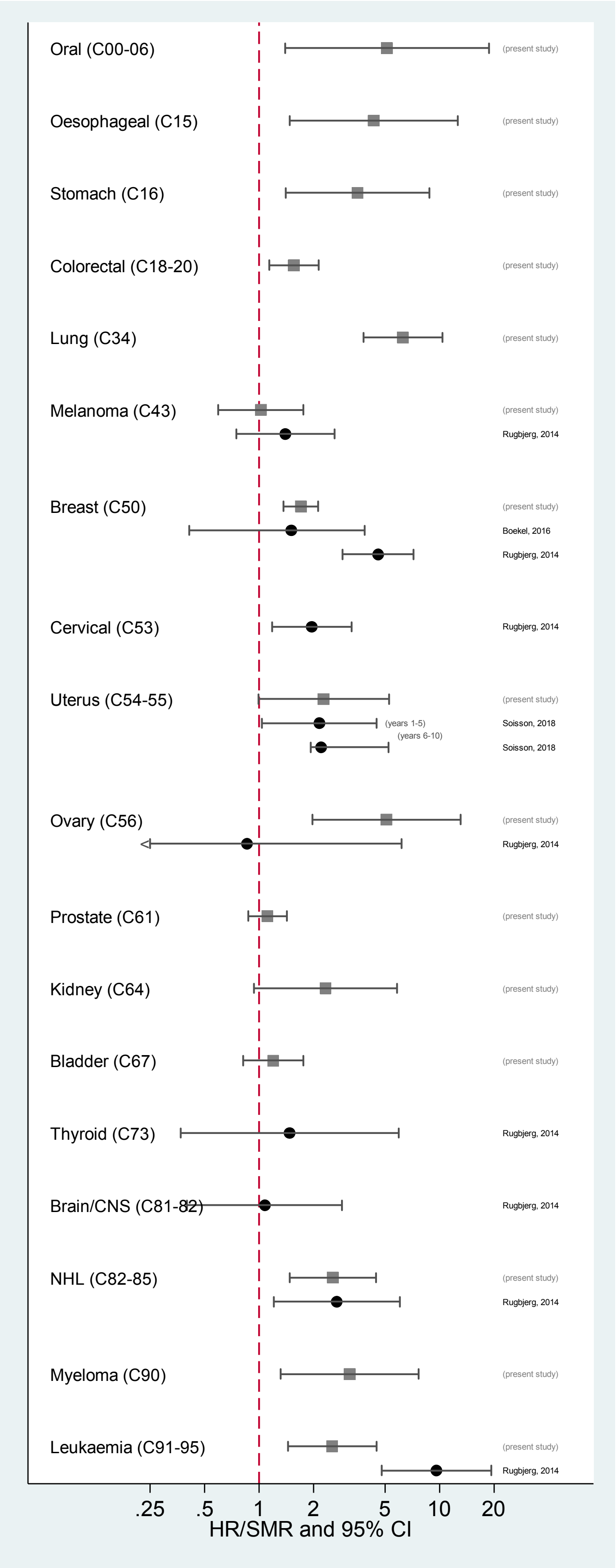

E) Peripheral vascular disease

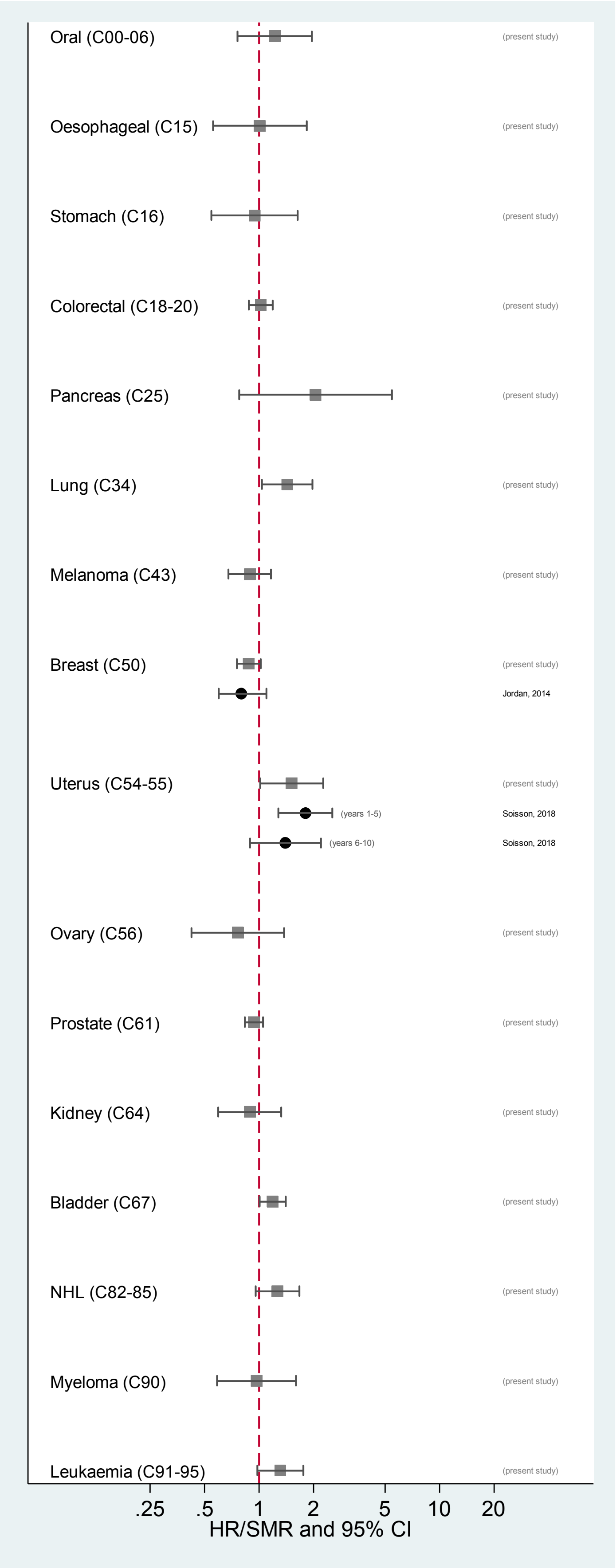

F) Stroke/cerebrovascular disease

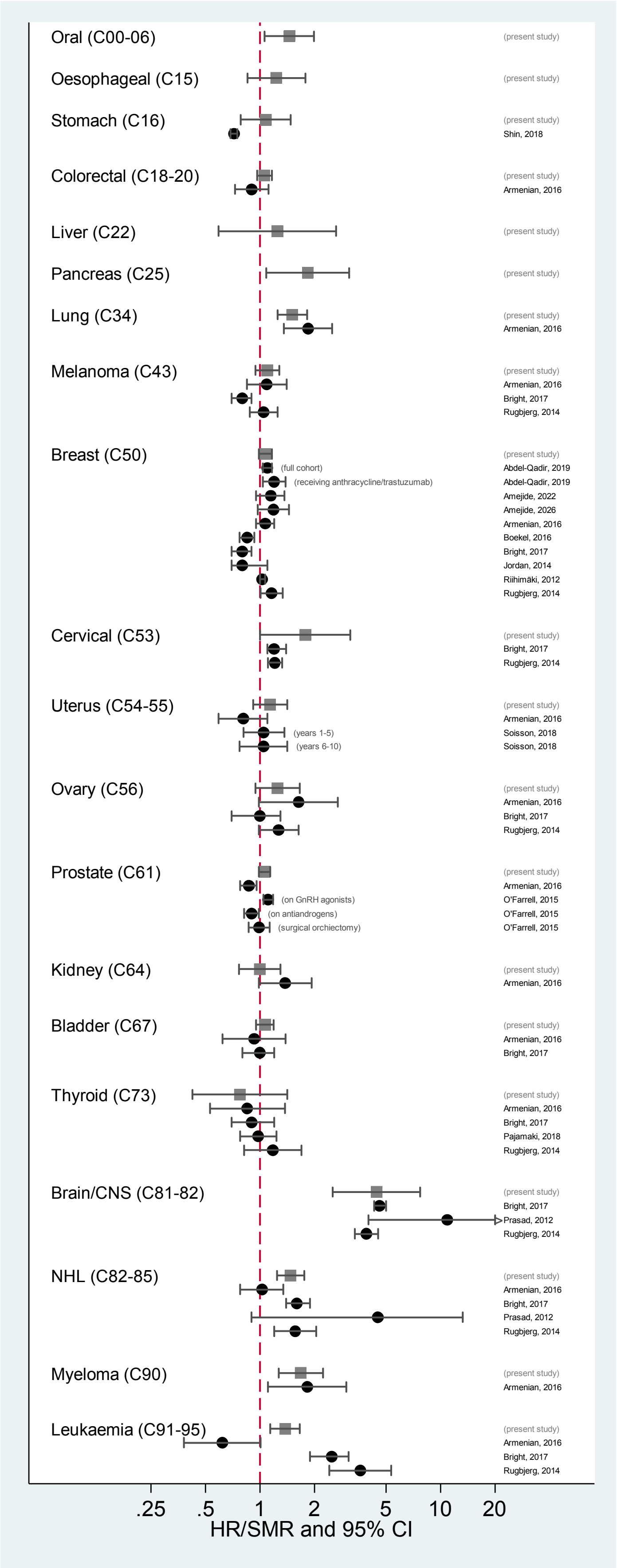

G) Ventricular heart disease

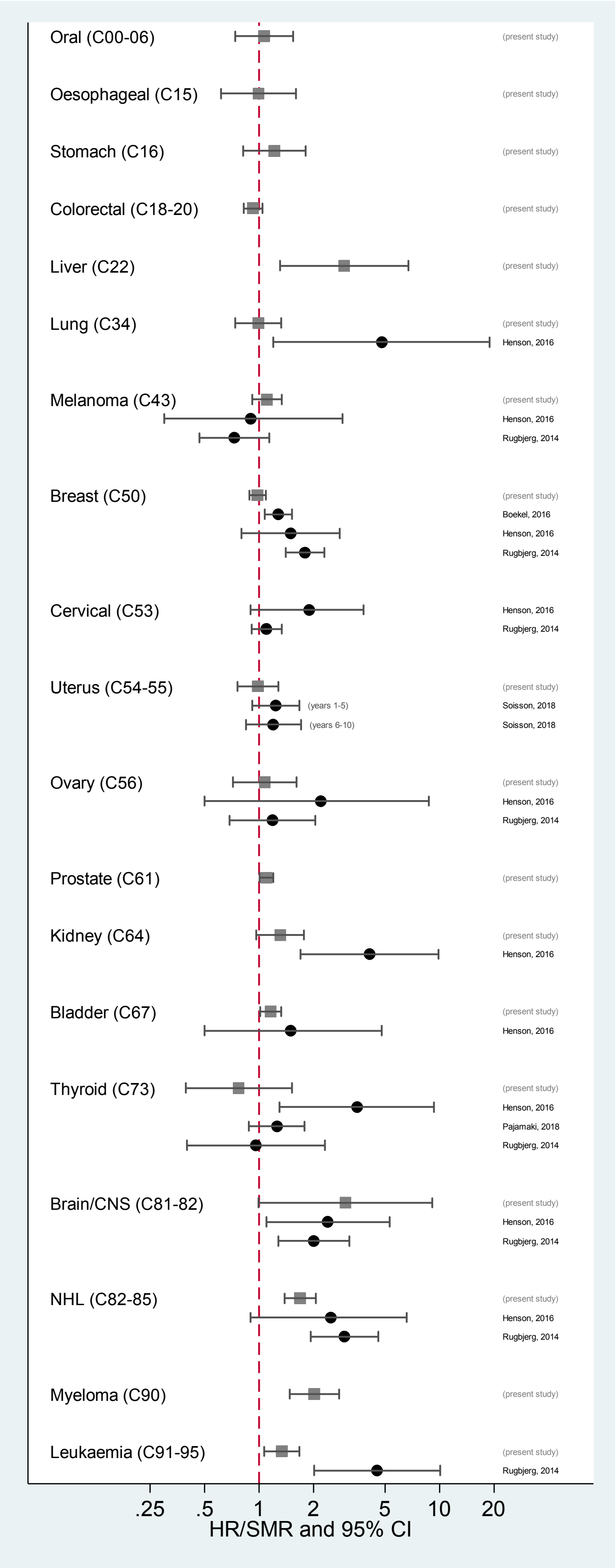

H) Venous thromboembolism

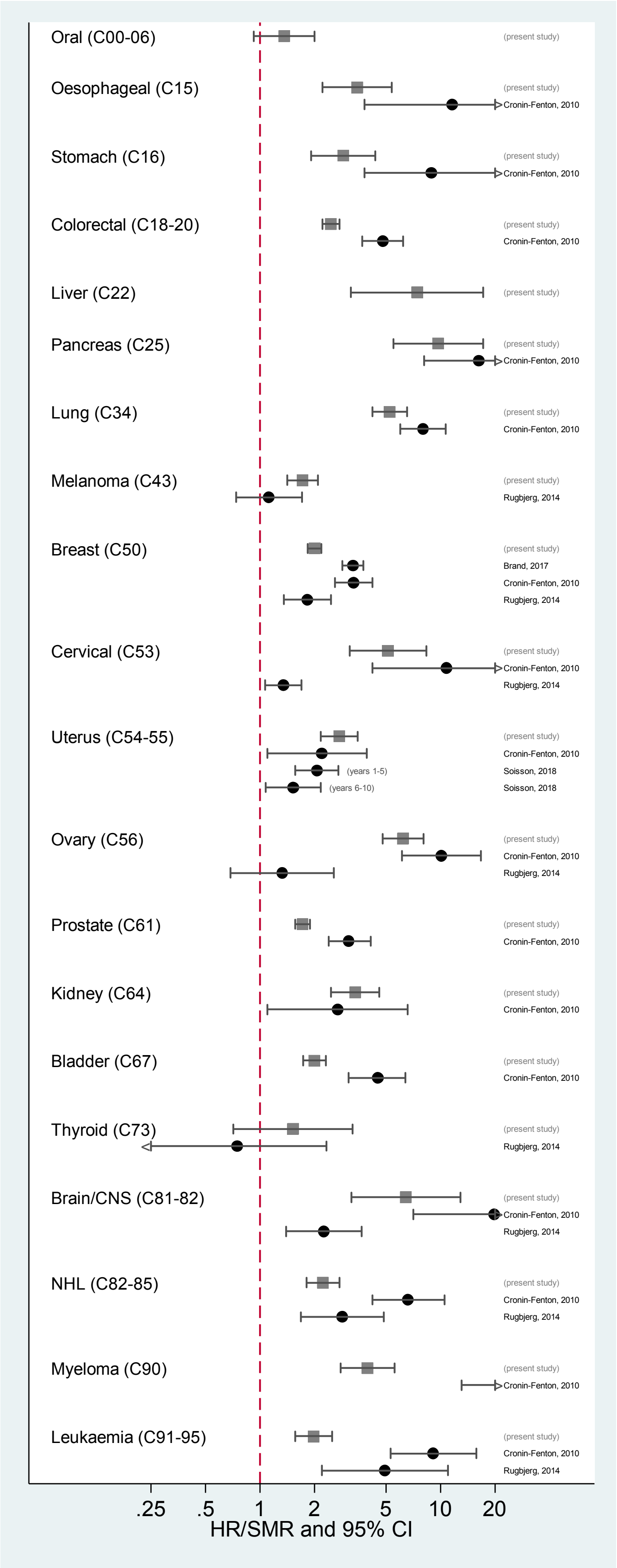

Reference list for review of previous studies

1 Abdel-Qadir H, Thavendiranathan P, Austin PC, *et al.* The Risk of Heart Failure and Other Cardiovascular Hospitalizations After Early Stage Breast Cancer: A Matched Cohort Study. *J Natl Cancer Inst* 2019; published online Jan 31. DOI:10.1093/jnci/djy218.

2 Ameijide A, Clèries R, Carulla M, *et al.* Cause-specific mortality after a breast cancer diagnosis: a cohort study of 10,195 women in Girona and Tarragona. *Clin Transl Oncol* 2019; published online Jan 3. DOI:10.1007/s12094-018-02015-5.

3 Armenian SH, Xu L, Ky B, *et al.* Cardiovascular Disease Among Survivors of Adult-Onset Cancer: A Community-Based Retrospective Cohort Study. *J Clin Oncol* 2016; **34**: 1122–30.

4 Boekel NB, Schaapveld M, Gietema JA, *et al.* Cardiovascular Disease Risk in a Large, Population-Based Cohort of Breast Cancer Survivors. *Int J Radiat Oncol Biol Phys* 2016; **94**: 1061–72.

5 Boerman LM, Berendsen AJ, van der Meer P, Maduro JH, Berger MY, de Bock GH. Long-term follow-up for cardiovascular disease after chemotherapy and/or radiotherapy for breast cancer in an unselected population. *Support Care Cancer* 2014; **22**: 1949–58.

6 Brand JS, Hedayati E, Bhoo-Pathy N, *et al.* Time-dependent risk and predictors of venous thromboembolism in breast cancer patients: A population-based cohort study. *Cancer* 2017; **123**: 468–75.

7 Bright CJ, Hawkins MM, Guha J, *et al.* Risk of Cerebrovascular Events in 178 962 Five-Year Survivors of Cancer Diagnosed at 15 to 39 Years of Age. *Circulation* 2017; **135**: 1194–210.

8 Chang JS, Shin J, Park E-C, Kim YB. Risk of cardiac disease after adjuvant radiation therapy among breast cancer survivors. *Breast* 2018; **43**: 48–54.

9 Cronin-Fenton DP, Søndergaard F, Pedersen LA, *et al.* Hospitalisation for venous thromboembolism in cancer patients and the general population: a population-based cohort study in Denmark, 1997–2006. *Br J Cancer* 2010; **103**: 947–53.

10 Henson KE, Reulen RC, Winter DL, *et al.* Cardiac Mortality Among 200 000 Five-Year Survivors of Cancer Diagnosed at 15 to 39 Years of Age: The Teenage and Young Adult Cancer Survivor Study. *Circulation* 2016; **134**: 1519–31.

11 Jordan JH, Thwin SS, Lash TL, *et al.* Incident comorbidities and all-cause mortality among 5-year survivors of Stage I and II breast cancer diagnosed at age 65 or older: a prospective-matched cohort study. *Breast Cancer Res Treat* 2014; **146**: 401–9.

12 Kenzik KM, Balentine C, Richman J, Kilgore M, Bhatia S, Williams GR. New-Onset Cardiovascular Morbidity in Older Adults With Stage I to III Colorectal Cancer. *J Clin Oncol* 2018; **36**: 609–16.

13 Kero AE, Järvelä LS, Arola M, *et al.* Cardiovascular morbidity in long-term survivors of early-onset cancer: A population-based study. *Int J Cancer* 2014; **134**: 664–73.

14 O'Farrell S, Garmo H, Holmberg L, Adolfsson J, Stattin P, Van Hemelrijck M. Risk and timing of cardiovascular disease after androgen-deprivation therapy in men with prostate cancer. *J Clin Oncol* 2015; **33**: 1243–51.

15 Pajamaki N, Metso S, Hakala T, *et al.* Long-term cardiovascular morbidity and mortality in patients treated for differentiated thyroid cancer. *Clin Endocrinol (Oxf)* 2018; **88**: 303–10.

16 Prasad PK, Signorello LB, Friedman DL, Boice JDJ, Pukkala E. Long-term non-cancer mortality in pediatric and young adult cancer survivors in Finland. *Pediatr Blood Cancer* 2012; **58**: 421–7.

17 Riihimaki M, Thomsen H, Brandt A, Sundquist J, Hemminki K. Death causes in breast cancer patients. *Ann Oncol* 2012; **23**: 604–10.

18 Rugbjerg K, Mellemkjaer L, Boice JD, Køber L, Ewertz M, Olsen JH. Cardiovascular disease in survivors of adolescent and young adult cancer: a Danish cohort study, 1943-2009. *J Natl Cancer Inst* 2014; **106**: dju110.

19 Salz T, Zabor EC, de Nully Brown P, *et al.* Preexisting Cardiovascular Risk and Subsequent Heart Failure Among Non-Hodgkin Lymphoma Survivors. *J Clin Oncol* 2017; **35**: 3837–43.

20 Shin DW, Suh B, Park Y, *et al.* Risk of Coronary Heart Disease and Ischemic Stroke Incidence in Gastric Cancer Survivors: A Nationwide Study in Korea. *Ann Surg Oncol* 2018; **25**: 3248–56.

21 Soisson S, Ganz PA, Gaffney D, *et al.* Long-term Cardiovascular Outcomes Among Endometrial Cancer Survivors in a Large, Population-Based Cohort Study. *JNCI J Natl Cancer Inst* 2018; published online May 8. DOI:10.1093/jnci/djy070.

Part D – original study protocol

(as approved by the Independent Scientific Advisory Committee for MHRA Database Research (ISAC) on 6th February 2017; deviations from protocol with justification are listed at the end

**Applicants must complete all sections listed below**<br><br>**Sections which do not apply should be completed as '*Not Applicable*'**
**A. Study Title§**<br>*§Please note: This information will be published on CPRD's website as part of its transparency policy*<br><br>**The risk of cardiovascular diseases among survivors of site-specific adult cancers**
**B. Lay Summary (Max. 200 words)§**<br>*§Please note: This information will be published on CPRD's website as part of its transparency policy*<br><br>As medical care has improved, more people are surviving cancer, and the number of survivors is growing. Cancer and its treatment may have an effect on the health of survivors throughout their life. There is thought to be a link between having a history of cancer and an increased chance of developing diseases that affect the heart and blood vessels (known as cardiovascular diseases, or CVD). Some studies have looked at how specific cancer treatments affect the chances of developing CVD, but there is a need for a comprehensive study covering a range of cancer types and cardiovascular outcomes. This study aims to find out how much a history of cancer affect the chances of developing CVD, for different types of cancer and cardiovascular disease. This will be investigated by comparing cancer survivors to similar individuals, but with no history of cancer, using medical records from primary care linked to national cancer and other databases. Our study will help to identify cancers that lead to a particularly high chance of getting certain cardiovascular diseases, which may help health services target interventions to prevent CVD, and will help identify priority areas for more detailed research on how specific cancers can lead to CVD.
**C. Technical Summary (Max. 200 words)§**<br>*§Please note: This information will be published on CPRD's website as part of its transparency policy*<br><br>As medical care has improved, an increasing number of individuals are surviving cancer, and the population of survivors is growing. Cancer and its treatment may impact on the health of survivors throughout life, and there is evidence for an association between having a history of cancer and an increased risk of cardiovascular diseases (CVD). Some studies have looked at how specific cancer treatments affect the chances of developing CVD, but there is a need for a comprehensive study covering a range of cancer types and cardiovascular outcomes. This study will use survival analysis methods within a matched cohort study design; cancer survivors will be compared to controls with no history of cancer, and associations between site-specific cancer history and risks of a range of cardiovascular diseases will be estimated. This will help identify cancers that lead to a particularly high risk of certain cardiovascular diseases, and help identify priority areas for future research in cardio-oncology.

Applicants must complete all sections listed below

Sections which do not apply should be completed as '*Not Applicable*'

D. Objectives, Specific Aims and Rationale

Overall aim: To investigate the associations between site-specific cancers and a range of CVD outcomes.

The aims are:

1. Calculate the incidence rates of different CVD outcomes among cancer survivors, in the years following diagnosis.
2. Investigate the association between site-specific cancer history and different CVD outcomes, and the extent to which these are driven by shared risk factors and other potential confounders:
    a. Estimate hazard ratios for associations between site-specific cancers and future risk of different CVD outcomes, with and without adjustment for shared risk factors and other potential confounders
    b. Investigate whether a history of cancer affects risks of CVD outcomes differently for different patient groups, such as age and sex groups, and those with pre-existing CVD.
3. Calculate cumulative incidences of specific CVD outcomes following site-specific cancer survival, accounting for competing events, and evaluate mortality burden over time due to CVD compared to cancer and other competing causes.

Rationale: There are now large numbers of cancer survivors in the UK and other parts of the world. This project will quantify the impact of site-specific cancer history on risk of a variety of CVD outcomes, including for rarer and less studied cancer sites. This will lay important foundations for further studies in the field of cardio-oncology, and give insight into where resources may best be directed to help cancer survivors reduce their CVD risk.

E. Study Background

Five-year cancer survival in England and Wales increased from an estimated 29.8% to 50.9% between 1971-2006, resulting in an increased number of cancer survivors within the population[1, 2]. Cancer, and cancer treatment, may have a broad range of impacts on the long-term health of these individuals and, in order to provide them with optimum healthcare, it is important to understand how a history of cancer, both the disease and the treatment, can affect health in later life.

There is evidence for an elevated risk of CVD in patients who have survived both childhood and adult cancers[3, 4], and one study suggests that CVD is now the leading cause of death among patients who have had breast cancer, with the cancer itself now being treated successfully in many cases[5]. A number of plausible mechanisms for the cardiovascular effects of different cancer treatments have also been identified[6]. To date, studies examining the relationship between cancer and CVD have tended to focus on cancers with large numbers of survivors, such as breast cancer, and there is relatively little information

Applicants must complete all sections listed below

Sections which do not apply should be completed as '*Not Applicable*'

available about the effects of rarer cancers, or those in whom there are fewer long-term survivors[3]. Many studies have also focussed specifically on the CVD effects of specific cancer treatments[6-9].

There have been a few broader studies of CVD after cancer, using datasets outside the UK. Rjugberg et al[10] followed a cohort of adolescent and young adult cancer survivors (aged 15-39 years at diagnosis between 1943 and 2009) from Danish health record data to examine their risk of CVD compared to matched controls. They found the risk of CVD remained increased throughout life following cancer survival. Particularly high risk of venous and lymphatic diseases was found, especially following breast and cervical cancer, and malignant melanoma. They also found substantially elevated risk of valvular heart disease, following Hodgkin's lymphoma; cerebral haemorrhage and cardiomyopathy, following leukaemia; and cardiomyopathy following non-Hodgkin Lymphoma. This study provided a broad overview of CVD in childhood and young adult cancer survivors, as well as information about some relationships that had not before been studied (for example, long term outcomes of cervical cancer). However, the most common cancer types differ between adults and children or adolescents, and there is evidence to suggest differences in the biology underlying certain tumours in these two groups[11, 12]. The impact of childhood and adult cancers on cardiovascular health may also differ. Another study, from the US, examined the risk of CVD in cancer survivors diagnosed at the age of 40 years or later, using health records from Southern California[13]. The authors found CVD incidence rates were increased in the whole cancer survivor population, compared to matched controls, but that incidence was unchanged in some cancers, or even lowered (prostate). Armenian et al limited their study to "clinically overt" diseases, and so do not examine risk for a number of types of CVD, such as valvular heart disease or arrhythmia.

This study aims to investigate whether survivors of site-specific adult cancers are at increased risk of specific cardiovascular outcomes compared to the general population. An association between cancer history and CVD risk may be due to a number of mechanisms, for example:

i) Cancer-associated biological changes directly increasing the risk of CVD (e.g. inflammation; changes in coagulation factors etc.)
ii) Cancer treatment increasing the risk of CVD (e.g. anthracyclines causing cardiac toxicitiy)
iii) Cancer history affecting psychological or behavioural factors that in turn affect risk of CVD (e.g. increased depression/anxiety, changes in attitudes to health care among cancer survivors)
iv) Increased health care monitoring/contact of cancer survivors leading to earlier detection of CVD
v) Common risk factors for cancer and CVD (e.g. smoking, high BMI etc)

This study will aim to describe overall associations, and where possible to disentangle the drivers of any differences in risk between cancer survivors and controls, though we acknowledge that we will not have access to all the information needed to perfectly disentangle mechanisms.

We will include a broad range of cancer types, including less studied cancers, and we will investigate a wide range of CVD outcomes. This will provide new insights into the associations between cancer and

Applicants must complete all sections listed below

Sections which do not apply should be completed as '*Not Applicable*'

future cardiovascular risk, which will be useful in planning health provisions for cancer survivors, and will identify priority areas for further research.

F. Study Type

This is a descriptive/hypothesis generating study.

G. Study Design

The study will use a matched cohort design (see sections K and L for details).

H. Feasibility counts

We have conducted initial feasibility counts using January 2014 CPRD data, which included 153552 cancer survivors overall. The number included by cancer site, and the anticipated number of events based on feasibility counts is shown in the table in section I (sample size considerations).

I. Sample size considerations

The table below shows the number included by cancer site and the anticipated numbers of key events based on feasibility counts. Since the study is hypothesis-generating, formal power calculations will not apply, but to give an idea of precision, the table also includes the expected hazard ratio we would expect to be able to conclusively detect (with 95% CI excluding the null) for each cancer/outcome pairing, given the anticipated numbers of events. For common cancers and CVD outcomes; we will be able to detect relatively small effects; for some of the less common cancer/CVD combinations (in grey), results are less likely to be conclusive unless the hazard ratios are large, but we have retained these because even statistically imprecise effect estimates will be informative in showing overall patterns and helping to generate hypotheses, and these can also feed into future meta-analyses. Imprecisely estimated associations will be presented with appropriate caution. The counts presented below used data from the January 2014 build, so it should be noted that we will have up to 3 years of extra data and more practices available in the final study, improving the power.

Applicants must complete all sections listed below

Sections which do not apply should be completed as '*Not Applicable*'

Site	Alive at 1y[a] (% of total diagnosed)	Mean years follow-up (sd)[a]	Expected events in cancer survivors plus controls (Minimum detectable HR for cancer survivor vs control [CPRD study])[b]				
			MI	Angina	Heart Failure	Stroke	VTE
Breast	41487 (84.5)	5.2 (4.3)	4245 (1.12)	5184 (1.11)	3648 (1.13)	2465 (1.16)	2199 (1.17)
Prostate	28510 (82.0)	4.1 (3.4)	7156 (1.09)	4936 (1.11)	6115 (1.10)	1749 (1.20)	1238 (1.24)
Colorectal	19214 (67.2)	4.0 (3.8)	3543 (1.13)	2773 (1.15)	2984 (1.15)	1044 (1.26)	816 (1.30)
Blood	13414 (70.1)	4.5 (3.9)	1906 (1.19)	1725 (1.20)	1565 (1.21)	805 (1.30)	629 (1.35)
Melanoma	10015 (84.5)	5.0 (4.3)	1271 (1.23)	1249 (1.24)	1033 (1.26)	633 (1.35)	511 (1.39)
Bladder	8312 (72.7)	4.5 (4.1)	1955 (1.19)	1413 (1.22)	1657 (1.20)	526 (1.39)	392 (1.46)
Lung	7625 (26.3)	2.0 (2.7)	723 (1.32)	668 (1.34)	612 (1.36)	248 (1.61)	194 (1.72)
Ovarian	3666 (63.7)	3.8 (4.1)	256 (1.60)	341 (1.50)	225 (1.65)	170 (1.78)	151 (1.84)
Uterus	2922 (79.8)	4.6 (4.2)	308 (1.53)	384 (1.47)	274 (1.57)	157 (1.82)	140 (1.89)
Oesophagus	2861 (36.3)	2.1 (2.8)	289 (1.56)	266 (1.59)	237 (1.63)	*102 (2.11)*	*77 (2.36)*
Myeloma	2712 (64.9)	3.2 (3.0)	365 (1.48)	327 (1.52)	306 (1.54)	*124 (1.96)*	*98 (2.14)*
Kidney	1751 (62.5)	4.3 (4.1)	263 (1.59)	245 (1.62)	216 (1.67)	*104 (2.09)*	*80 (2.32)*
Cervix	1721 (73.7)	4.6 (4.4)	*83 (2.28)*	*117 (2.00)*	*72 (2.43)*	*93 (2.18)*	*83 (2.28)*
Stomach	1698 (33.6)	2.7 (3.4)	249 (1.61)	189 (1.73)	213 (1.67)	*70 (2.46)*	*54 (2.78)*
Brain/CNS	1602 (35.2)	3.3 (3.7)	*57 (2.71)*	*78 (2.34)*	*39 (3.33)*	*76 (2.37)*	*59 (2.66)*
Testicular	1427 (87.1)	5.8 (4.7)	*88 (2.23)*	*104 (2.09)*	*45 (3.07)*	*117 (2.00)*	*83 (2.28)*
Oral	1329 (69.8)	4.1 (3.9)	*4.1 (3.9)*	175 (1.77)	172 (1.77)	138 (1.90)	*75 (2.38)*
Thyroid	1106 (78.3)	5.2 (4.6)	*73 (2.41)*	*98 (2.14)*	*58 (2.68)*	*70 (2.46)*	*59 (2.66)*
Larynx	1069 (76.0)	4.6 (4.0)	216 (1.67)	186 (1.74)	178 (1.76)	*70 (2.46)*	*52 (2.84)*
Pancreas	974 (17.4)	1.7 (2.6)	*82 (2.29)*	*72 (2.43)*	*68 (2.49)*	*29 (4.04)*	*23 (4.79)*
Liver	711 (26.3)	1.9 (2.5)	*56 (2.73)*	*55 (2.76)*	*46 (3.03)*	*23 (4.79)*	*18 (5.88)*

[a]From the January 2014 CPRD build.
[b]Expected numbers of cardiovascular events are based on amount of person-time follow-up assuming 5 controls per cancer survivor, and published cardiovascular disease incidence rates, taking account the age and sex structure where stratified rates were available.
Minimum detectable HRs are based on projected events and assume 80% power, alpha=0.05
Grey & italic indicates cells where the minimum detectable HR is >2
MI = myocardial infarction, VTE = venous thromboembolism.

Applicants must complete all sections listed below<br><br>Sections which do not apply should be completed as ‘*Not Applicable*’
**J. Data Linkage Required (if applicable):**[§]<br>[§]*Please note that the data linkage/s requested in research protocols will be published by the CPRD as part of its transparency policy*<br><br>Data linkage will be requested for:<br>National cancer registry: This will provide improved identification of cancer cases and more detailed information on them, such as stage, grade and treatment.<br><br>Hospital episode statistics (HES): As many cardiovascular events and cancer diagnoses will be recorded in hospitals, this will provide more complete capture of these events.<br><br>Office for National Statistics (ONS): This will provide more accurate information on cardiovascular deaths that may not be recorded elsewhere, and will provide the cause of death breakdown needed for Aim 3 (evaluating cumulative incidence of CVD death vs cancer death vs other deaths, among cancer survivors).<br><br>Index of multiple deprivation (IMD) data: we would like these data to adjust for potential confounding by socioeconomic status. We have requested practice level IMD so that we can adjust for this in analyses using CPRD GOLD data only, which includes the full cohort; we have also requested patient-level IMD so that in secondary/sensitivity analyses among linked patients only, we can adjust more closely for IMD.<br><br>Primary analyses will be carried out using only CPRD GOLD primary care data, to maximise sample size/power. Including linked data will improve ascertainment for cancer/CVD events, and provide detail on cancer stage, grade and treatment, but will restrict the sample to patients in linked practices, and the follow up time to within coverage dates Therefore, sensitivity analyses will be carried out to identify if this restriction/improved ascertainment alters estimates. In addition, some secondary analyses will be carried out in which linkage is required. See also section N.<br><br>In analyses where linkages are used, these will be restricted to those eligible for the relevant linkages, and will take account of the relevant linkage coverage dates.
**K. Study population**<br><br>The study population will consist of all individuals with no CVD history and an incident adult cancer in CPRD (defined as a first ever record of cancer at age 18 years or more, recorded at least 12 months after

Applicants must complete all sections listed below<br><br>Sections which do not apply should be completed as '*Not Applicable*'
start of research-standard CPRD follow-up) who are alive and under follow-up 12 months after their diagnosis, and their matched controls (described in section L).
**L. Selection of comparison group(s) or controls**<br><br>Cancer survivors (i.e. the "exposed" group) will be matched to up to 5 controls who are alive and under follow-up with no history of cancer 12 months after the cancer diagnosis of the exposed patients (hereafter referred to as "index date"). Controls are required to have 24 months' follow-up in CPRD prior to the index date. The 24-month criterion is for similarity to exposed patients, who are required to have had at least 12 months in CPRD before and 12 months after the cancer diagnosis. Controls will be matched to exposed patients by age (as near and possible and within ±3 years), sex, and GP practice.
**M. Exposures, Health Outcomes§ and Covariates**<br>*§Please note: Summary information on health outcomes (as included on the ISAC application form above)will be published on CPRD's website as part of its transparency policy*<br><br>**Exposure**<br><br>Exposure will be survival of cancer. Cancer survivors will be grouped into cohorts dependent on the cancer-site. Cancer types will be identified using up to date Read code lists, following the methods used by Bhaskaran et al[14]. The search strategy for finding the relevant Read codes, used in that paper, is reproduced in the appendix. The cancers included were chosen based on including all those with at least 1000 1-year survivors in our feasibility counts (see section I), with the addition of liver and pancreatic cancers, which fell below 1000 anticipated 1-year survivors due to poor short term survival, but were considered to be important enough to include in the light of their relatively high incidence (it should be noted that in the updated data which will include up to 3 years' more data, there are likely to be more 1-year survivors for both of these cancers).<br><br>Thus, the complete list of cancers to be considered as exposures is:<br><br>• Bladder<br>• Breast<br>• Cervical<br>• Central nervous system<br>• Colorectal<br>• Gastric<br>• Kidney<br>• Laryngeal<br>• Leukaemia<br>• Liver

- Lung
- Melanoma
- Myeloma
- Non-Hodgkin Lymphoma
- Oesophagus
- Oral
- Ovary
- Pancreas
- Prostate
- Testicular
- Thyroid
- Uterus

Outcomes

The outcomes will be incident diagnoses of the following cardiovascular endpoints, which are shown with their ICD codes (each modelled separately) – Read code lists for CVD outcomes are presented in the appendix and were derived from previous code lists developed as part of the CALIBER project[15], with update by KB and clinical review by LS.

- Vascular disease and complications
  - Angina (I20)
  - Myocardial infarction (I21)
  - Revascularisation procedures (OCPS-4: K40-46)
  - Sudden cardiac arrest (I46)
  - Peripheral vascular disease (I73) (PVD)
  - Stroke (haemorrhagic and ischaemic) (I60-I64)
- Heart failure and related
  - Cardiomyopathy (I42-I43, I25.5)
  - Heart failure (I50)
  - Left ventricular dysfunction
- Arrhythmia (I48-I49)
- Venous thromboembolism
  - Deep venous thromboembolism (I80.1-I80.3) (DVT)
  - Pulmonary Embolism (I26) (PE)
- Pericarditis (130-I32)
- Valvular heart disease (I01-I08, I34-I37)

Applicants must complete all sections listed below<br><br>Sections which do not apply should be completed as '*Not Applicable*'

Many stroke codes in CPRD do not distinguish between haemorrhagic and ischaemic stroke, and thus these will be analysed as one outcome, despite their different pathogenesis. The Oxford Vascular Study found haemorrhagic stroke made up approximately 16% of incident stroke diagnoses in 2002-4[16].

In addition to cardiovascular outcomes, death due to CVD, cancer, and other causes will be recorded for the analyses in aim 3.

Covariates

Covariates to be included to address confounding will be informed by the development of directed acyclic graphs (DAGs), drawn for each site-specific cancer/CVD outcome relationship. DAGs represent hypothesised causal relationships between variables (informed by *a priori* knowledge and the available data), and can be used to crystallise the assumptions being made when choosing covariates for adjustment. Some covariates, such as BMI, will not be fixed over time. However, adjustment will only be made for the baseline measure, as only this measure can causally influence both the exposure and outcome (BMI measures occurring after the exposure/baseline, cannot cause it, and thus cannot confound the relationship between exposure and outcome).

As per section L, exposed and unexposed patients will be matched on age, sex and GP practice. Based on preliminary DAGs, other covariates (all measured at the time of cancer diagnosis) to be included in our modelling are:

- BMI (calculated from height and weight records where available, or as entered directly)
- Smoking
- Alcohol consumption
- Diabetes status
- Contraceptive pill/HRT use (in women), within 2 years prior to diagnosis
- CVD diagnosis prior to cancer diagnosis
- Statin use within two years prior to cancer diagnosis
- Blood-pressure lowering drug use within two years prior to cancer diagnosis
- Individual-level index of multiple deprivation (in analyses using linked data only)

N. Data/ Statistical Analysis

Follow-up will begin at the index date for both cancer survivors and matched controls, which is the one-year anniversary of diagnosis for cancer survivors, and the same date for matched controls.

Primary analyses: CPRD data only

Applicants must complete all sections listed below

Sections which do not apply should be completed as '*Not Applicable*'

Aim 1 – calculate CVD incidence rates for cancer survivors and matched controls:

We will calculate crude age- and sex-stratified incidence rates for each outcome among cancer survivor and control individuals, by cancer site, and time since index date (annually up to 5 years since index date, then 5-yearly).

Aim 2 – investigate unadjusted and adjusted associations between cancer and CVD outcomes:

a) We will fit Cox proportional hazard models to obtain hazard ratios for each outcome comparing cancer survivor and control patients, initially accounting only for matching factors, and then adjusting for covariates (see section K) to investigate the extent to which associations are driven by shared risk factors and other confounders. Separate models will be fitted for each cancer site, and we will account for the matching by stratifying on matched set, which allows for a different baseline hazard function for each matched set, but with common covariate effects[17]. We will check for proportional hazards by fitting interactions with time since index date, and if necessary we will present time-stratified effect estimates.
b) We will also investigate whether the estimated effect of cancer history on CVD differs between population groups by fitting interactions with: age group, sex (for cancers affecting both genders), smoking status, body mass index category, pre-existing CVD (at index date), and deprivation category.

Aim 3 – calculate cumulative incidences accounting for competing risks

For the analyses above, the competing risk of death without the outcome of interest will be censored, (which estimates the cause-specific hazard, giving a causal interpretation assuming appropriate adjustment for confounding)[18]. We will also estimate cumulative incidences of each outcome in the presence of competing risks, to evaluate the true public health burden due to cardiovascular disease in the cancer survivor population.

Secondary analyses: CPRD and linked data

Analyses for aims 1-3 will be repeated including linked data, which will improve identification of outcome and exposure events, but limit sample size and follow up time. The results from these will be compared to those in the primary analyses to identify any major changes in estimates.

In addition to this, the analysis in aim 2 will be extended to investigate differences in the association between cancer history and CVD risk in cancer stage and grade groups (available in linked cancer registry data). To do this, the exposure variable (cancer history) will be changed to a categorical stage/grade variable, including a category for 'no cancer'. For breast cancer, differences in risk by hormone receptor/HER2 status will be similarly investigated. The extent to which cancer treatment mediates the effect of cancer history on CVD risk will also be investigated using treatment information recorded in the

Applicants must complete all sections listed below<br>Sections which do not apply should be completed as '*Not Applicable*'
cancer registry linked data[19, 20]; this analysis will be limited to cancers with sufficient variation in treatment modalities.<br><br>Finally, to compare mortality burden from CVD over time with mortality from other causes, we will use cause of death from ONS mortality data to estimate the cumulative incidences of death due to CVD, cancer, and other causes, in each case treating the others as competing risks.<br><br>**Note on multiple comparisons**<br><br>We are aware that this protocol looks at a number of cancer sites and several CVD outcomes. We are classifying this study as hypothesis generating and not hypothesis testing, so formal corrections for multiple testing (which in any case can involve problematic assumptions) are not considered appropriate. Results will be presented as hypothesis generating in publications and the possibility of spurious results due to multiple comparisons will be discussed; associations for which there is less than overwhelming evidence will be described with appropriate caution; replication of any positive findings in other datasets will be encouraged.
**O. Plan for addressing confounding**<br><br>Confounding by age, sex and GP practice will be addressed by matching of exposed and unexposed patients on these factors. Age will also be included as a covariate if controls cannot be matched to the same birth year. In addition, variables assumed to be confounders from our DAGs will be included in survival models as covariates; a provisional list is provided in section M.
**P. Plans for addressing missing data**<br><br>For the exposure and outcome variables and some covariates in this study, the presence or absence of a code will be used to assign patients to one of two groups. The absence of a code is assumed to mean the condition is not present, so missing data (i.e. incorrectly absent codes) will not be identifiable, and thus cannot be addressed as part of the study.<br><br>For some covariates in the primary care data, notably smoking status, alcohol use, and BMI, there will be some explicitly missing data. The recording of these variables is unlikely to be conditionally independent of the variable values themselves in the primary care setting, so would not satisfy the assumption of missing at random (MAR) for multiple imputation. The assumption made for complete case analysis – that the probability of a variable value being missing is independent of the outcome given the variable value, and other covariates in the model being fitted – is more likely to be met[21]. Therefore, complete case analysis (with respect to variables not set by the presence or absence of a code) will be conducted. If there are large amounts of missing data (>30%) then we will also conduct sensitivity analysis under a range of non-random missingness mechanisms[22].

Applicants must complete all sections listed below

Sections which do not apply should be completed as '*Not Applicable*'

In the linked cancer registry data, there may be some missing data in cancer stage, grade and treatment variables. Missingness in these variables will be described, and analyses of effect modification/mediation by these variables will be restricted to matched sets in which these data are complete for the cancer exposed patient.

Q. Patient or user group involvement (if applicable)

No patient or user group involvement will occur

R. Plans for disseminating and communicating study results, including the presence or absence of any restrictions on the extent and timing of publication

Results will be disseminated by publication via conference presentations, and in peer reviewed scientific journals.

S. Limitations of the study design, data sources, and analytic methods

- **Unmeasured confounding**
  Some factors that are likely to influence cardiovascular disease and cancer development will not be measured in CPRD. For example, genetic factors, diet and physical activity. For some of these, proxy measures may partially account for the confounding (for example, BMI as a proxy for diet and physical activity). This is, however, not possible for variables such as genetics, and so some residual confounding will remain. This is a limitation of using primary care databases, and will be discussed in outputs.
- **Sample size**
  The number of patients with some combinations of site-specific cancer history and specific cardiovascular disease will be small, for example, if there a few survivors and the CVD is rarer. This will reduce precision for some effect estimates. However, imprecise estimates are still useful for showing general patterns, and also for use in future meta analyses. Imprecise estimates will be presented with appropriate caution.
- **Cancer treatment data**
  Data on the specific details of cancer, used in the secondary analyses, and its treatment are limited. While linkage to the cancer registry improves the amount of detail available, only basic treatment details are available, and these will not allow us to differentiate completely between the biological effects of cancer history on CVD risk and those mediated by cancer treatment.

Applicants must complete all sections listed below

Sections which do not apply should be completed as '*Not Applicable*'

T. References

1. Quaresma M, Coleman MP, Rachet B. 40-year trends in an index of survival for all cancers combined and survival adjusted for age and sex for each cancer in England and Wales, 1971-2011: a population-based study. Lancet (London, England). 2015 Mar 28;385(9974):1206-18
2. Initiative NCS. Living with and beyond cancer: taking action to improve outcomes (an update to the 2010 National Cancer Survivorship Initiative Vision). In: Department of Health MCS, NHS Improvement, editor. 2013.
3. Nathan PC, Amir E, Abdel-Qadir H. Cardiac Outcomes in Survivors of Pediatric and Adult Cancers. Canadian Journal of Cardiology. 2016;32(7):871-80
4. Carver JR, Shapiro CL, Ng A, Jacobs L, Schwartz C, Virgo KS, et al. American Society of Clinical Oncology clinical evidence review on the ongoing care of adult cancer survivors: cardiac and pulmonary late effects. J Clin Oncol. 2007 Sep 1;25(25):3991-4008
5. Patnaik JL, Byers T, DiGuiseppi C, Dabelea D, Denberg TD. Cardiovascular disease competes with breast cancer as the leading cause of death for older females diagnosed with breast cancer: a retrospective cohort study. Breast Cancer Research. 2011;13(3):R64
6. Moslehi JJ. Cardiovascular Toxic Effects of Targeted Cancer Therapies. New England Journal of Medicine. 2016;375(15):1457-67
7. Shelburne N, Adhikari B, Brell J, Davis M, Desvigne-Nickens P, Freedman A, et al. Cancer treatment-related cardiotoxicity: current state of knowledge and future research priorities. J Natl Cancer Inst. 2014 Sep;106(9)
8. Truong J, Yan AT, Cramarossa G, Chan KK. Chemotherapy-induced cardiotoxicity: detection, prevention, and management. The Canadian journal of cardiology. 2014 Aug;30(8):869-78
9. Yeh ET, Bickford CL. Cardiovascular complications of cancer therapy: incidence, pathogenesis, diagnosis, and management. J Am Coll Cardiol. 2009 Jun 16;53(24):2231-47
10. Rugbjerg K, Mellemkjaer L, Boice JD, Kober L, Ewertz M, Olsen JH. Cardiovascular disease in survivors of adolescent and young adult cancer: a Danish cohort study, 1943-2009. J Natl Cancer Inst. 2014 Jun;106(6):dju110
11. Parsons DW, Li M, Zhang X, Jones S, Leary RJ, Lin JC, et al. The genetic landscape of the childhood cancer medulloblastoma. Science (New York, NY). 2011 Jan 28;331(6016):435-9
12. Siegel RL, Miller KD, Jemal A. Cancer statistics, 2015. CA: A Cancer Journal for Clinicians. 2015;65(1):5-29
13. Armenian SH, Xu L, Ky B, Sun C, Farol LT, Pal SK, et al. Cardiovascular Disease Among Survivors of Adult-Onset Cancer: A Community-Based Retrospective Cohort Study. Journal of Clinical Oncology. 2016;34(10):1122-30
14. Bhaskaran K, Douglas I, Forbes H, dos-Santos-Silva I, Leon DA, Smeeth L. Body-mass index and risk of 22 specific cancers: a population-based cohort study of 5.24 million UK adults. Lancet (London, England). 2014 Aug 30;384(9945):755-65
15. Denaxas SC, George J, Herrett E, Shah AD, Kalra D, Hingorani AD, et al. Data resource profile: cardiovascular disease research using linked bespoke studies and electronic health records (CALIBER). Int J Epidemiol. 2012 Dec;41(6):1625-38
16. Rothwell PM, Coull AJ, Giles MF, Howard SC, Silver LE, Bull LM, et al. Change in stroke incidence, mortality, case-fatality, severity, and risk factors in Oxfordshire, UK from 1981 to 2004 (Oxford Vascular Study). The Lancet. 2004 6/12/;363(9425):1925-33
17. Collett D. Modelling Survival Data in Medical Research, Third Edition: CRC Press; 2015.

Applicants must complete all sections listed below

Sections which do not apply should be completed as '*Not Applicable*'

18. Geskus RB. Cause-Specific Cumulative Incidence Estimation and the Fine and Gray Model Under Both Left Truncation and Right Censoring. Biometrics. 2011;67(1):39-49
19. VanderWeele TJ. Causal mediation analysis with survival data. Epidemiology (Cambridge, Mass). 2011;22(4):582-5
20. VanderWeele TJ. Unmeasured confounding and hazard scales: sensitivity analysis for total, direct, and indirect effects. European Journal of Epidemiology. 2013 2013//;28(2):113-7
21. White IR, Carlin JB. Bias and efficiency of multiple imputation compared with complete-case analysis for missing covariate values. Stat Med. 2010 Dec 10;29(28):2920-31
22. Carpenter J, Kenward M. Multiple Imputation and its Application: Wiley; 2012.

Deviations from approved protocol with justification

Use of linked data

Contrary to expectations, we had similar power for our linked and primary care only analyses. This was mainly because of improved ascertainment of outcomes with the use of linked data. Ascertainment of cancer survivorship is also improved with the use of linked data. We therefore used linked data in our primary analysis and CPRD GOLD data as a sensitivity analyses.

ONS mortality data were used to define CVD outcomes in addition to CPRD GOLD and HES APC data.

Study population and variable definitions

We did not exclude individuals with a history of CVD prior to their first cancer diagnosis from the main study population. Instead, we excluded cancer survivors and controls with the CVD of interest prior to diagnosis from the relevant analyses. We included other CVD prior to baseline as a covariate and in interaction analyses.

We modified the CVD categories as follows in line with changes to previous studies following internal and reviewer comments. We split the original vascular disease and complications group into the following three groups: coronary artery disease, peripheral vascular disease and stroke (haemorrhagic and ischaemic). We classified left ventricular dysfunction as heart failure instead of a separate category.

As described in the protocol, we further developed our directed acyclic graphs (DAGs) prior to analysis. We identified additional covariates and added them to our analysis unless they were rare (to avoid problems with sparse data). We did not include use of the contraceptive pill as pre-specified as it has a complex association with CVD.

Statistical analysis

We have reported overall crude incidence in cancer survivors and control groups with our relative risk estimates. However, our analyses of crude incidence stratified separately by age, gender and time were difficult to summarise and interpret. We have therefore replaced these with graphs of predicted incidence over time stratified by age and estimated absolute incidence and excess incidence at 5 years.

We added calendar time and hypertension to the pre-specified interactions analyses and used Likelihood ratio tests to support assessments of effect estimation.

We restricted additional variables using cancer registry data to assessments of treatment effects as missingness of stage and grade was too high.
